# Supplementary material for: PDE7A inhibition suppresses triple-negative breast cancer by attenuating de novo pyrimidine biosynthesis
Source: Cell Rep Med. 2025 Sep 16;6(9):102356. doi: 10.1016/j.xcrm.2025.102356 (PMC12490241; doi:10.1016/j.xcrm.2025.102356)
Supplement: Document S1. Figures S1–S18 [file mmc1.pdf]

Cell Reports Medicine, Volume *6*

Supplemental information

PDE7A inhibition suppresses triple-negative breast cancer by attenuating *de novo* pyrimidine biosynthesis

Parmanand Malvi, Suresh Bugide, Roshan Dutta, Kiran Kumar Reddi, Yvonne J.K. Edwards, Kamaljeet Singh, Romi Gupta, and Narendra Wajapeyee

SUPPLEMENTARY FIGURES and LEGENDS

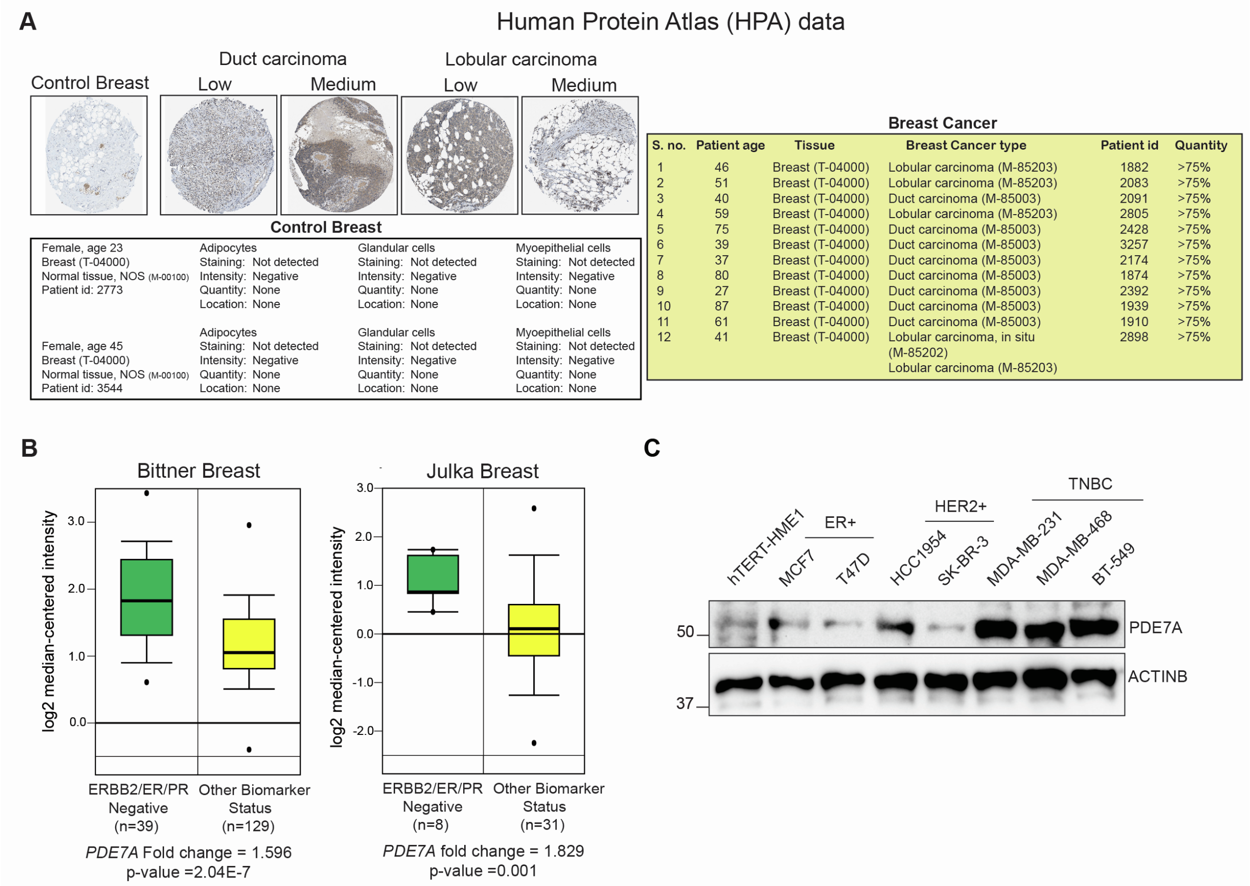

Figure S1. PDE7A is overexpressed in TNBC. Related to Figure 1. A. Relative expression of PDE7A protein in patient-derived breast cancer samples. Representative image of PDE7A protein expression in normal breast tissue and breast cancer samples from the Human Protein Tissue Atlas. **B**. *PDE7A* mRNA expression levels in TNBC samples compared with other breast cancer subtypes (ERBB2+, ER+ and ERBB2/ER+) in the indicated datasets. Fold changes and p-values for the indicated databases under indicated conditions are shown. **C.** Immunoblot analysis of PDE7A expression in non-transformed human mammary epithelial cells (hTERT-HME1) and in the breast cancer cell lines of the indicated subtypes. ACTINB was used as a loading control.

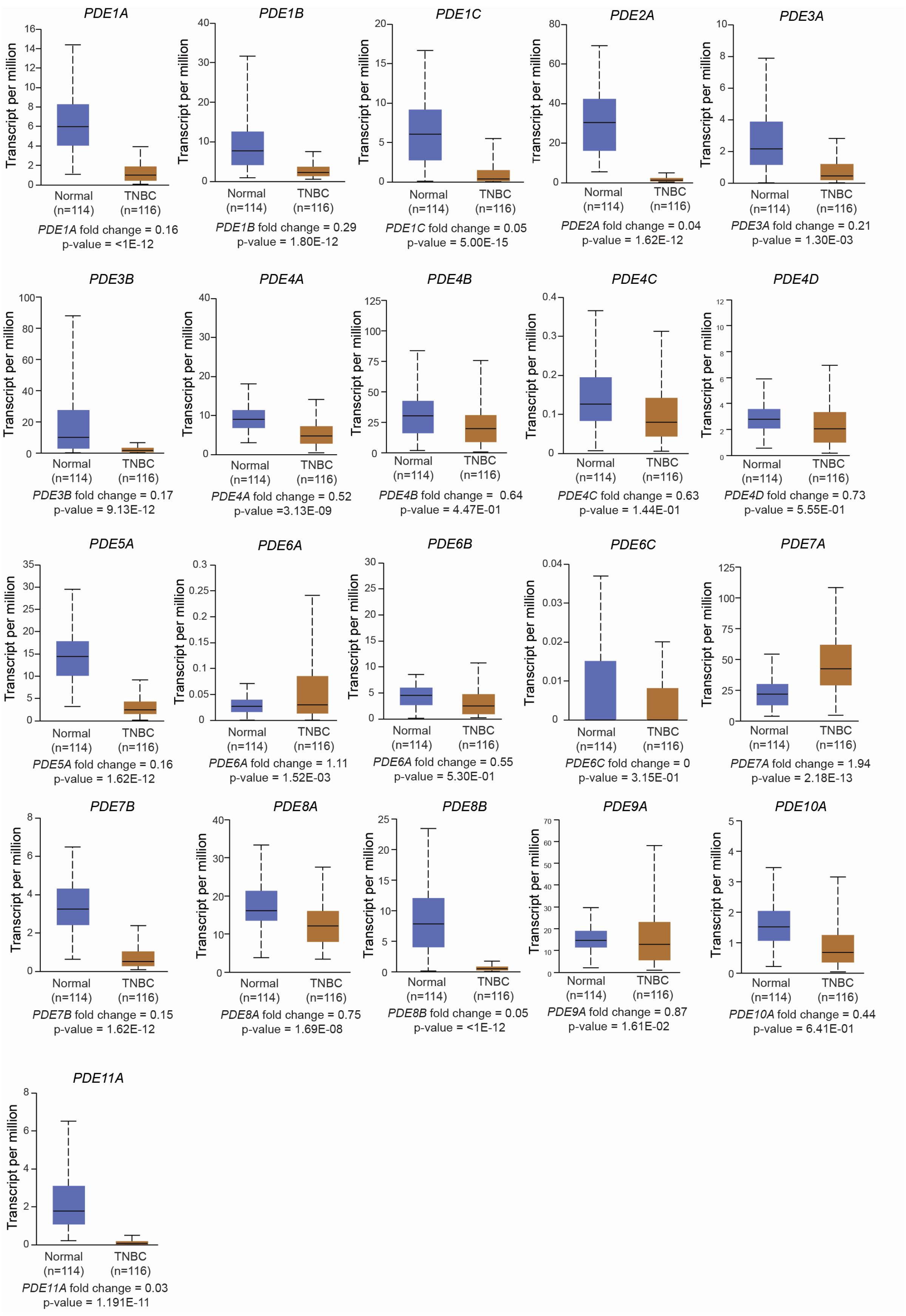
Transcript per million
Normal (n=114)
TNBC (n=116)
PDE1A
PDE1A fold change = 0.16
p-value = <1E-12
PDE1B
PDE1B fold change = 0.29
p-value = 1.80E-12
PDE1C
PDE1C fold change = 0.05
p-value = 5.00E-15
PDE2A
PDE2A fold change = 0.04
p-value = 1.62E-12
PDE3A
PDE3A fold change = 0.21
p-value = 1.30E-03
PDE3B
PDE3B fold change = 0.17
p-value = 9.13E-12
PDE4A
PDE4A fold change = 0.52
p-value =3.13E-09
PDE4B
PDE4B fold change = 0.64
p-value = 4.47E-01
PDE4C
PDE4C fold change = 0.63
p-value = 1.44E-01
PDE4D
PDE4D fold change = 0.73
p-value = 5.55E-01
PDE5A
PDE5A fold change = 0.16
p-value = 1.62E-12
PDE6A
PDE6A fold change = 1.11
p-value = 1.52E-03
PDE6B
PDE6A fold change = 0.55
p-value = 5.30E-01
PDE6C
PDE6C fold change = 0
p-value = 3.15E-01
PDE7A
PDE7A fold change = 1.94
p-value = 2.18E-13
PDE7B
PDE7B fold change = 0.15
p-value = 1.62E-12
PDE8A
PDE8A fold change = 0.75
p-value = 1.69E-08
PDE8B
PDE8B fold change = 0.05
p-value = <1E-12
PDE9A
PDE9A fold change = 0.87
p-value = 1.61E-02
PDE10A
PDE10A fold change = 0.44
p-value = 6.41E-01
PDE11A
PDE11A fold change = 0.03
p-value = 1.191E-11

Figure S2. Expression of phosphodiesterases (PDEs) in TNBC. Related to Figure 1. mRNA expression for all phosphodiesterases (PDEs) in the TNBC samples compared to normal breast tissue samples in the breast cancer TCGA dataset analyzed using UALCAN. Fold changes and p-values for individual PDE mRNAs compared to normal breast tissue samples are shown.

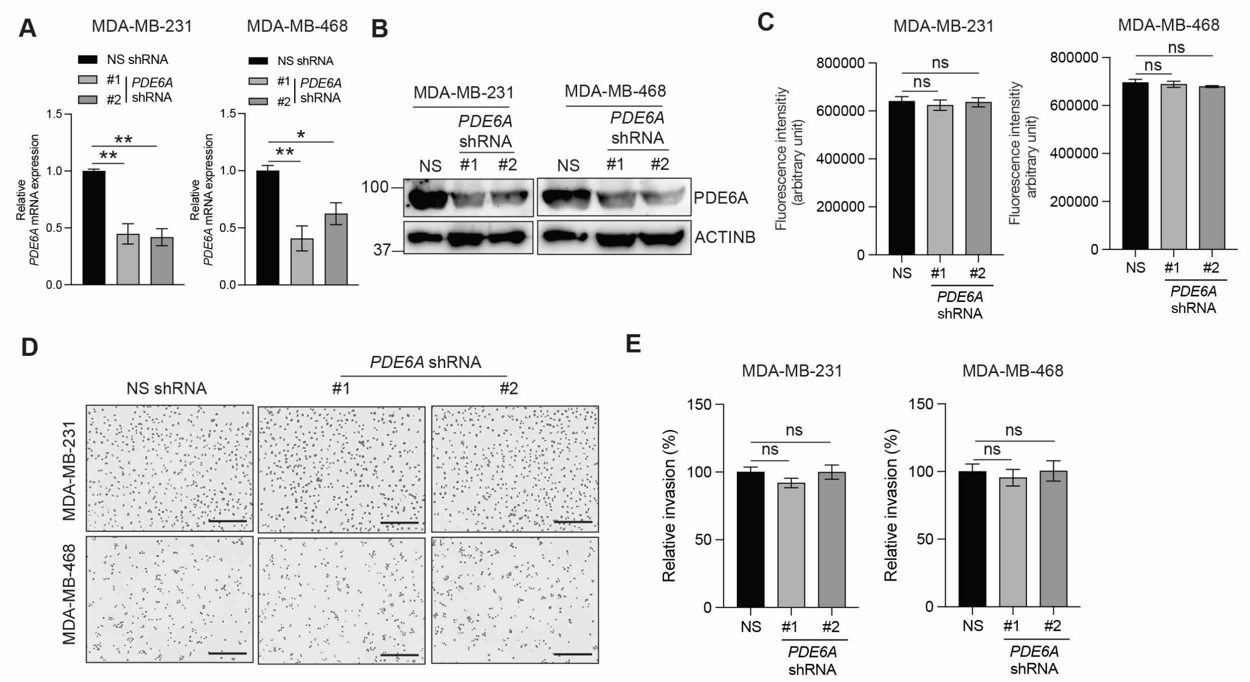

Figure S3. PDE6A knockdown does not suppress TNBC growth and invasion ability. Related to Figure 1. A. Indicated TNBC cell lines expressing either non-specific (NS) shRNA or *PDE6A* shRNAs were analyzed for the expression of *PDE6A* mRNA expression by RT-qPCR (n = 3 biological replicates/group). *PDE6A* mRNA expression is plotted under indicated conditions. *ACTINB* was used as a normalization control. **B.** Indicated TNBC cell lines expressing either NS shRNA or *PDE6A* shRNAs were analyzed for the expression of PDE6A protein by immunoblotting. PDE6A protein expression is shown under indicated conditions. ACTINB was used as a loading control. **C.** Indicated TNBC cell lines expressing NS shRNA or *PDE6A* shRNAs were analyzed by quantitative soft-agar assay using the CytoSelect 96-well quantitative soft agar assay. Fluorescence intensities (arbitrary unit) under the indicated conditions are shown (n = 3 biological replicates/group). **D.** Indicated TNBC cell lines expressing NS shRNA or *PDE6A* *s*hRNAs were analyzed for invasive capacity using a Matrigel-based invasion assay. Representative images are shown. Scale bar, 200 µm. **E.** Relative invasion (%) relative to the NS shRNA expressing cells is plotted for the experiment shown in panel D (n = 3 biological replicates/group). All quantitative data represent the mean ± SEM. ns= not significant p-value, *p < 0.05, **p < 0.01.

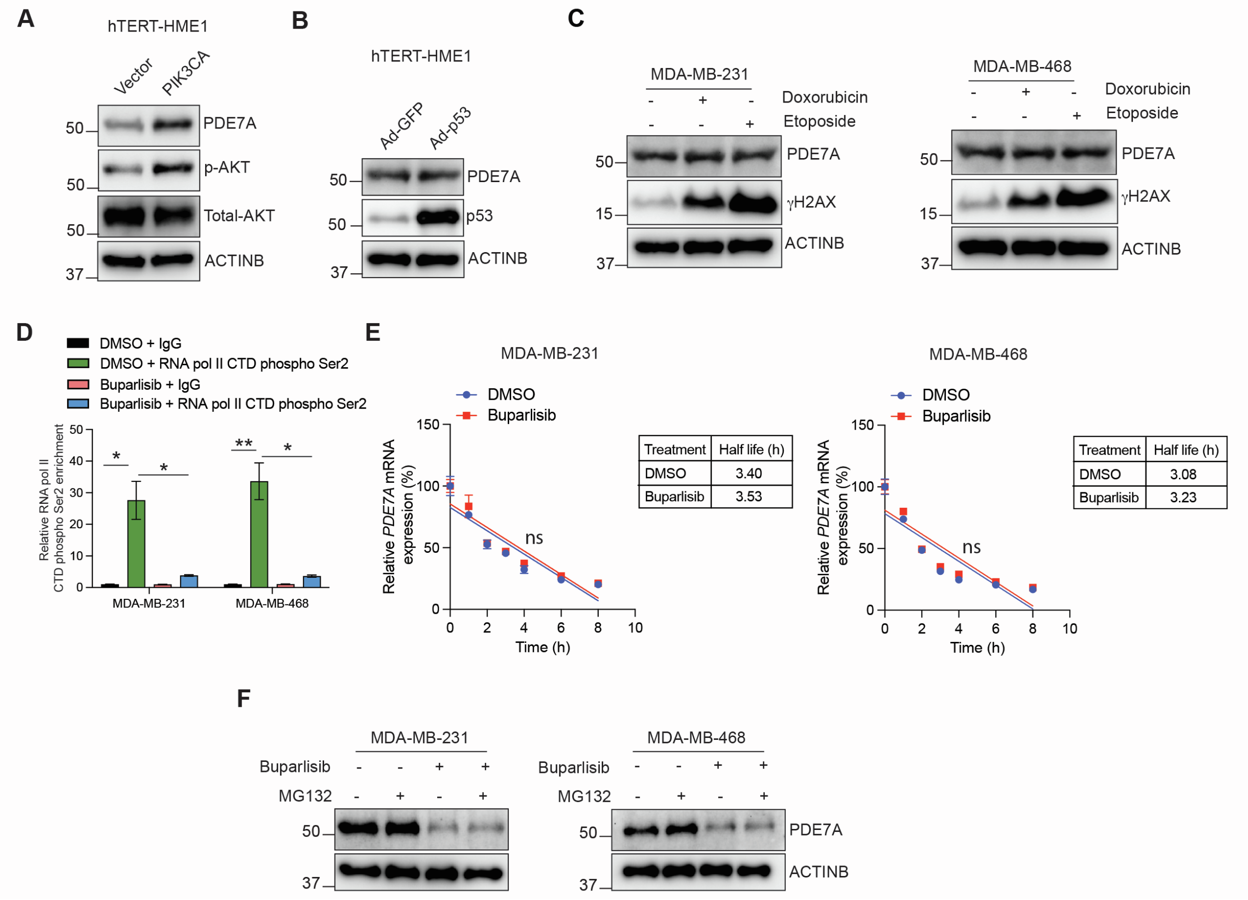

Figure S4. PI3K kinase stimulates PDE7A expression independent of mRNA stability and protein stability in TNBC cells. Related to Figure 2. A. Immunoblot analysis of the expression of the indicated proteins in PIK3CA-expressing hTERT-HME1 cells relative to vector-expressing cells. ACTINB was used as a loading control. **B.** Immunoblot analysis of the expression of the indicated proteins in adenoviral p53 (Ad-p53)-expressing hTERT-HME1 cells relative to vector (Ad-GFP)-expressing cells. ACTINB was used as a loading control. **C.** The indicated TNBC cell lines were treated with DMSO, doxorubicin (1 µM) or etoposide (25 µM) for 24 h. PDE7A protein expression was measured using immunoblot analysis under the indicated conditions. ACTINB was used as a loading control. **D.** Indicated TNBC cell lines were treated with DMSO or PI3K inhibitor buparlisib (200 nM) for 48 h and enrichment of RNA pol II CTD phospho Ser2 was measured relative to control anti-IgG antibody using chromatin immunoprecipitation (ChIP) assay. Enrichment under indicated conditions for RNA pol II CTD phospho Ser2 on PDE7A gene body relative to DMSO treated indicated cells is shown (n = 3 biological replicates/group). **E.** Indicated TNBC cell lines were treated with either DMSO or the PI3K inhibitor buparlisib (200 nM) for 48 h, followed by treatment with actinomycin D (5 µg/mL) for the indicated time points, and analyzed for *PDE7A* mRNA expression by RT-qPCR (n = 3 biological replicates/group). Relative mRNA expression levels for each condition and the half-life is shown. The half-life of *PDE7A* mRNA was calculated using simple linear regression in GraphPad Prism software (version 10.0, for Macintosh) and is shown. **F.** The indicated TNBC cell lines were treated with either DMSO or the

PI3K inhibitor buparlisib (200 nM) for 48 h, followed by treatment with the proteasome inhibitor MG132 (5 μM) for additional 16 h, and were analyzed for PDE7A protein expression by immunoblot analysis. ACTINB was used as a loading control. ACTINB was used as a loading control. All quantitative data represent the mean ± SEM. ns= not significant, *p<0.05, **p<0.01.

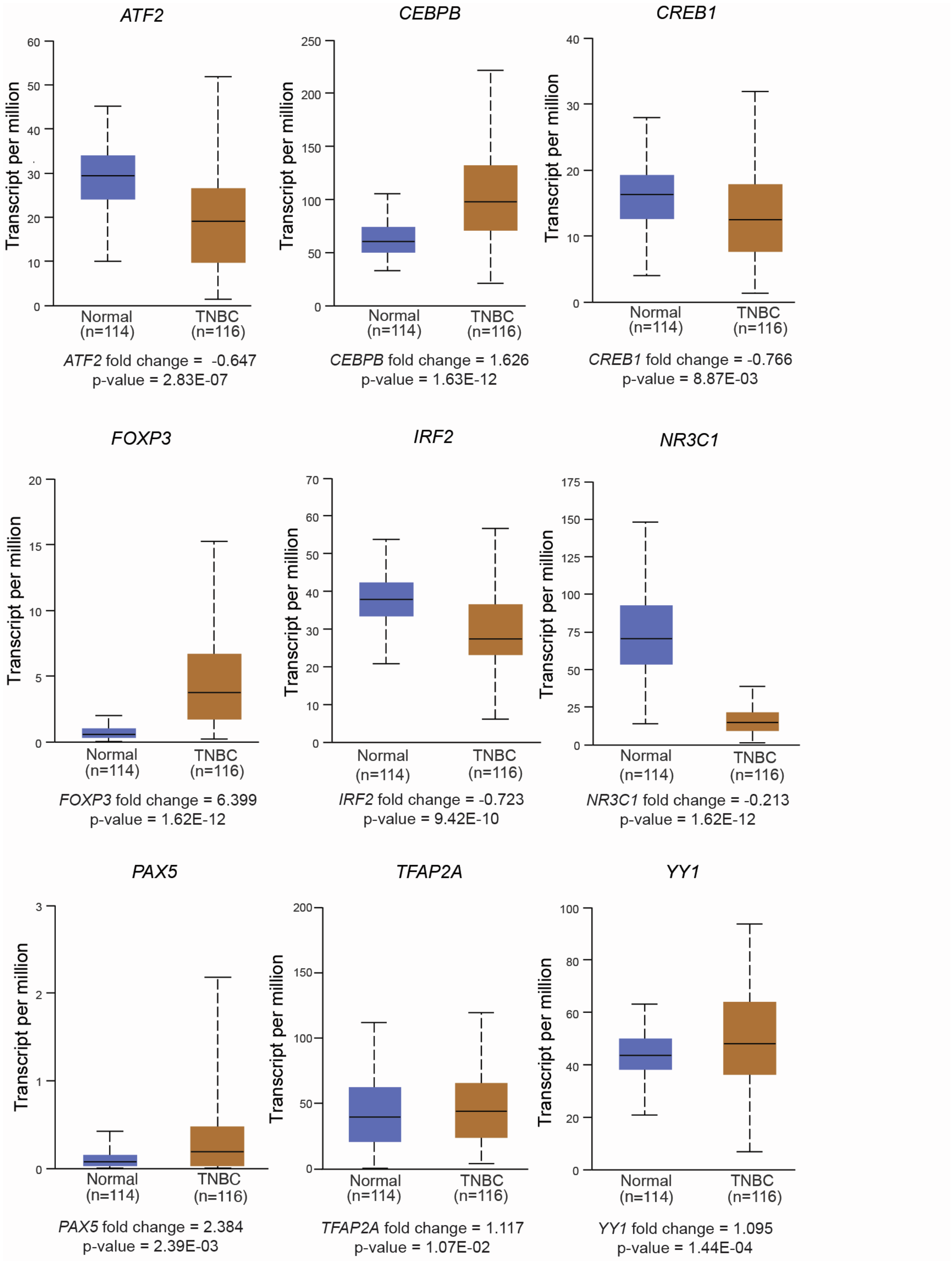

Figure S5. mRNA expression of indicated transcription factors in TNBC. Related to Figure 2. Using UALCAN, the mRNA expression of the indicated transcription factors in TNBC was examined by comparing normal breast tissue samples with those from TNBC in the TCGA dataset. Fold changes and p-values for indicated transcription factors are shown.

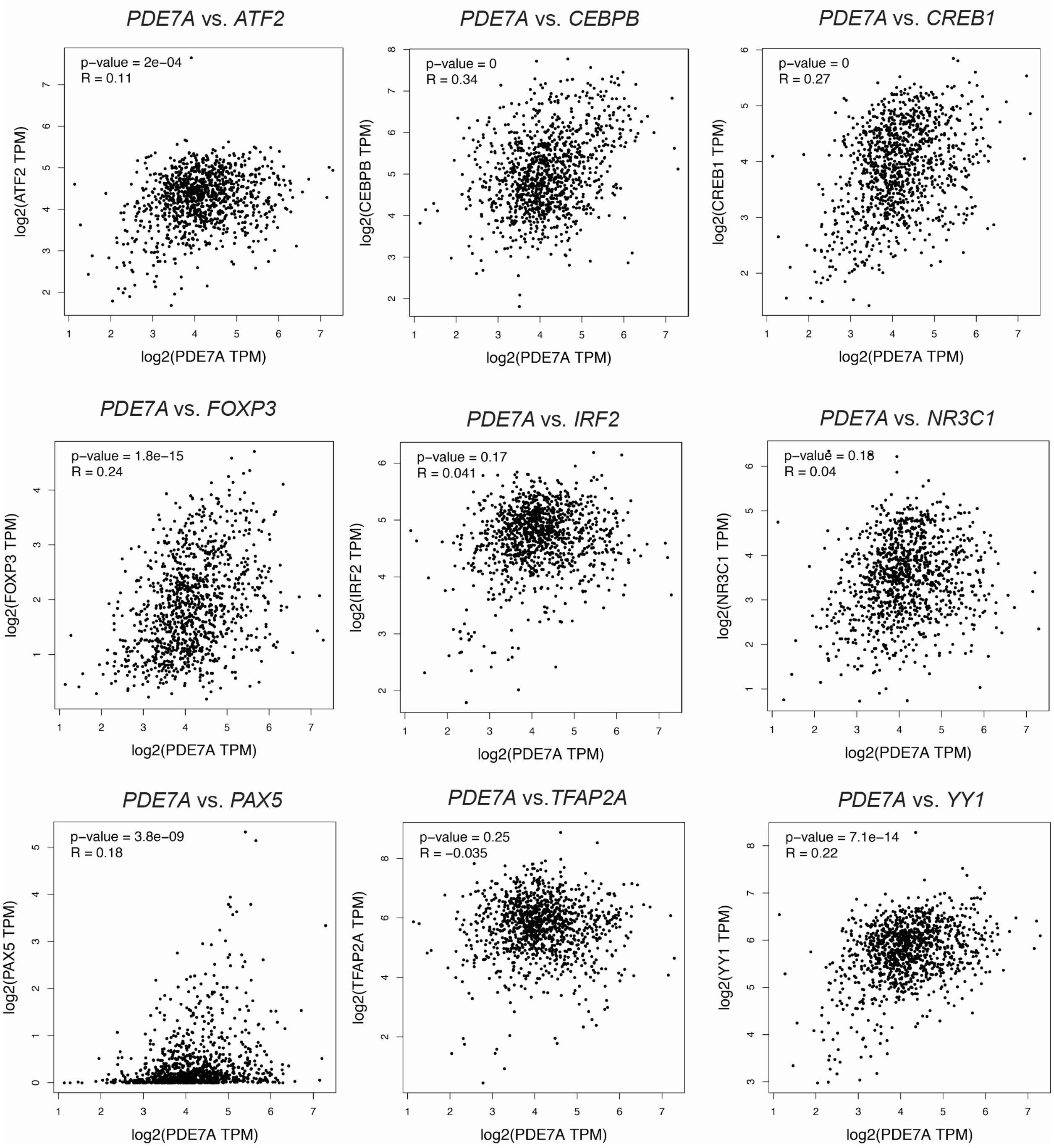

Figure S6. Correlation analysis for indicated transcription factors with a potential role in stimulating *PDE7A* transcription in TNBC. Related to Figure 2. A correlation analysis between the mRNA levels of the indicated transcription factors and *PDE7A* mRNA was performed using GEPIA on the TCGA dataset. Pearson correlation coefficient (R) and p-values for each correlation analysis are shown.

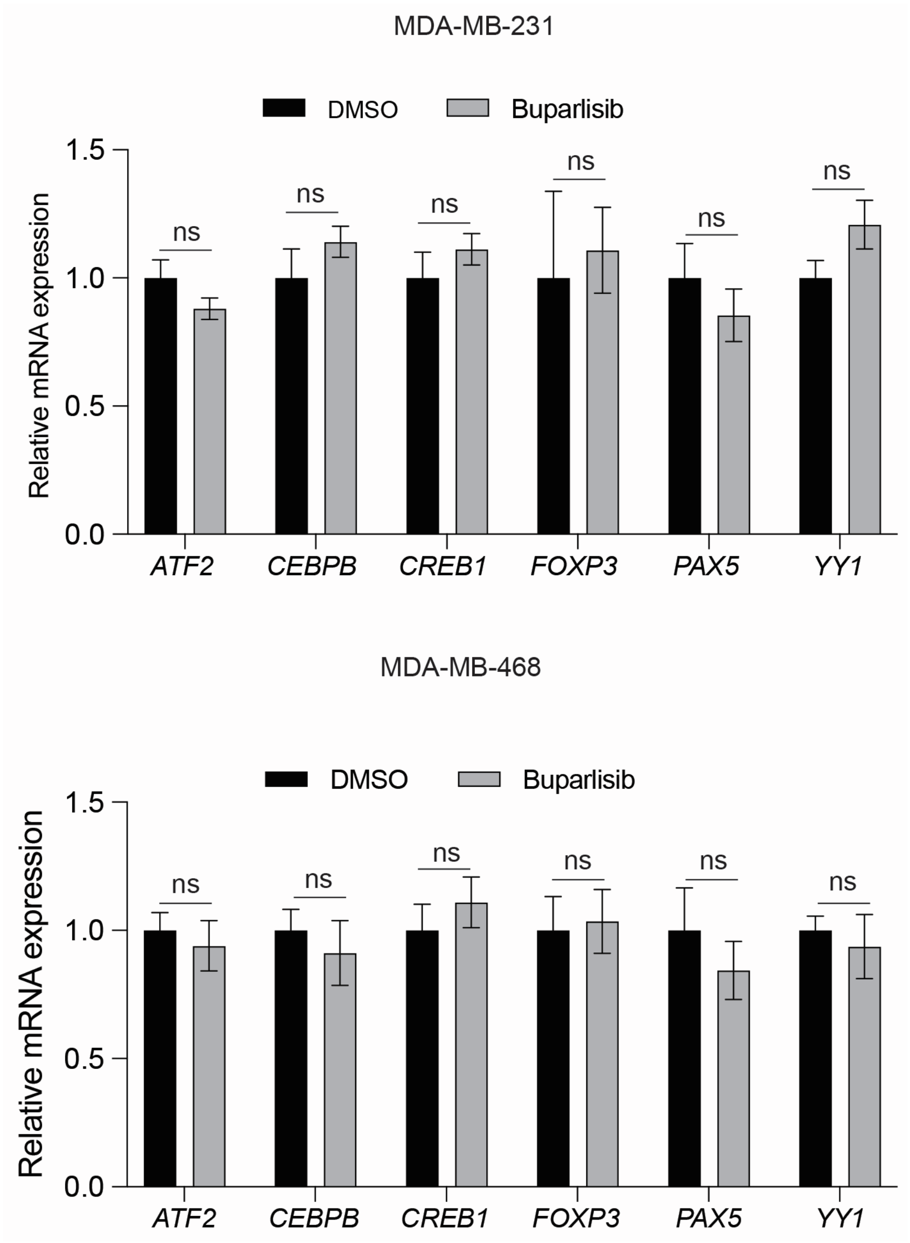

Figure S7. Measurement of indicated transcription factors following PI3K inhibitor buparlisib-treatment. Related to Figure 2. The indicated TNBC cell lines were treated with DMSO or buparlisib (200 nM) for 48 h and analyzed for the expression of indicated mRNA by RT-qPCR. Relative mRNA expression in buparlisib-treated cells relative to DMSO-treated cells is plotted (n = 3 biological replicates/group). *ACTINB* was used as a normalization control. All quantitative data represent the mean ± SEM. ns= not significant p-values.

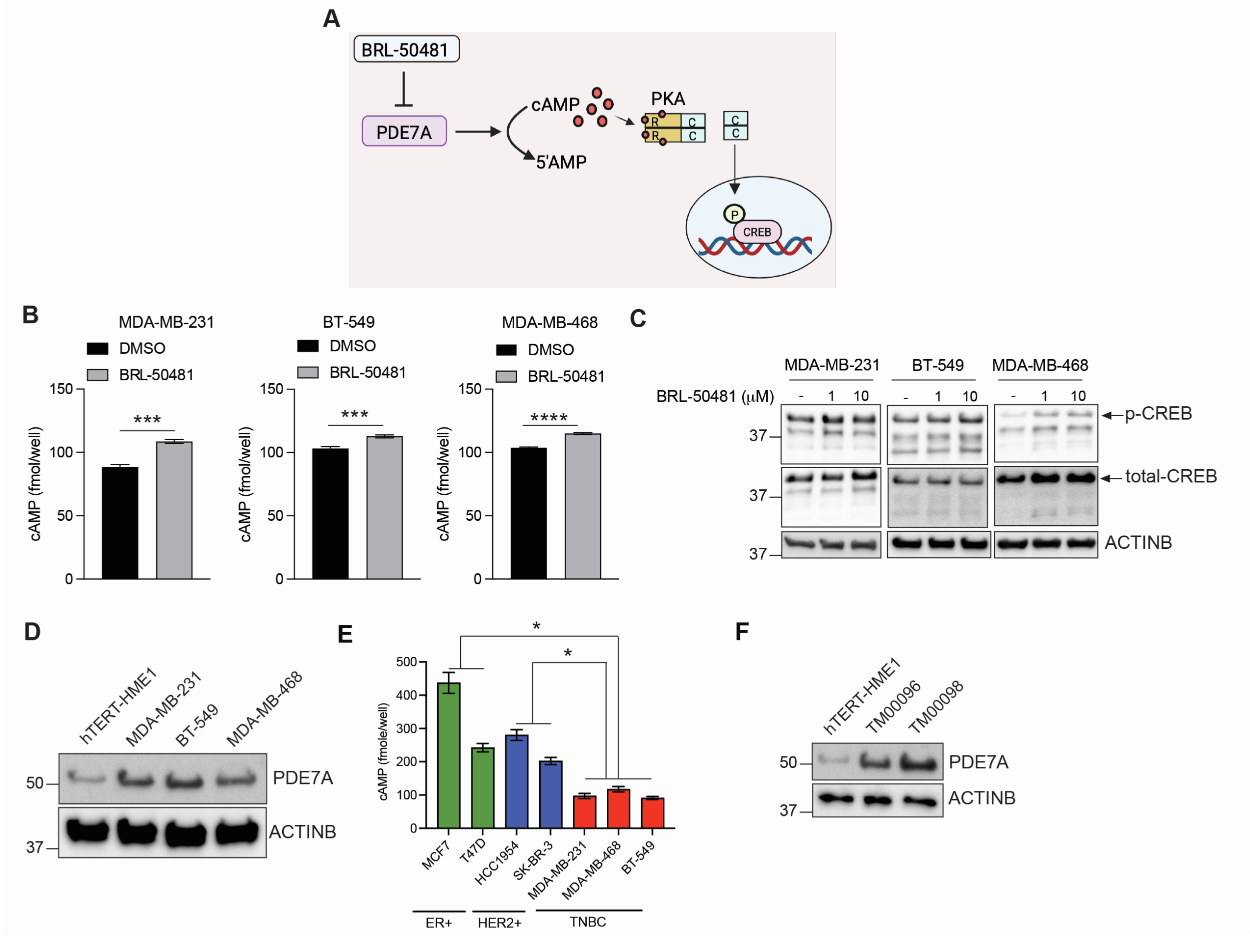

Figure S8. PDE7A is overexpressed in TNBC cells and regulates cAMP and CREB. Related to Figure 3. A. Schematic showing phosphodiesterase 7A (PDE7A) regulation of phosphorylated cyclic adenosine monophosphate (cAMP)-response element–binding protein (p-CREB) and the mechanism through which PDE7A inhibition activates CREB. **B.** The indicated TNBC cell lines were treated with DMSO or BRL-50481 (10 μM) for 72 h, and cAMP levels were measured (n = 3 biological replicates/group). **C.** The indicated TNBC cell lines were treated with DMSO or BRL-50481 at the indicated concentrations for 72 h, and p-CREB and total CREB levels were measured. ACTINB was measured as a loading control. **D.** PDE7A protein expression in the indicated TNBC cell lines compared to the non-transformed human mammary epithelial cells (hTERT-HME1). ACTINB was measured as a loading control. **E.** cAMP levels in the indicated cells lines. cAMP levels were measured in the indicated breast cancer cell lines of indicated breast cancer subtypes (n = 3 biological replicates/group). **F.** PDE7A protein expression in indicated TNBC PDXs compared to the hTERT-HME1 cells. ACTINB was measured as a loading control. All quantitative data represent the mean ± SEM. *p<0.05, ***p<0.001, ****p<0.0001.

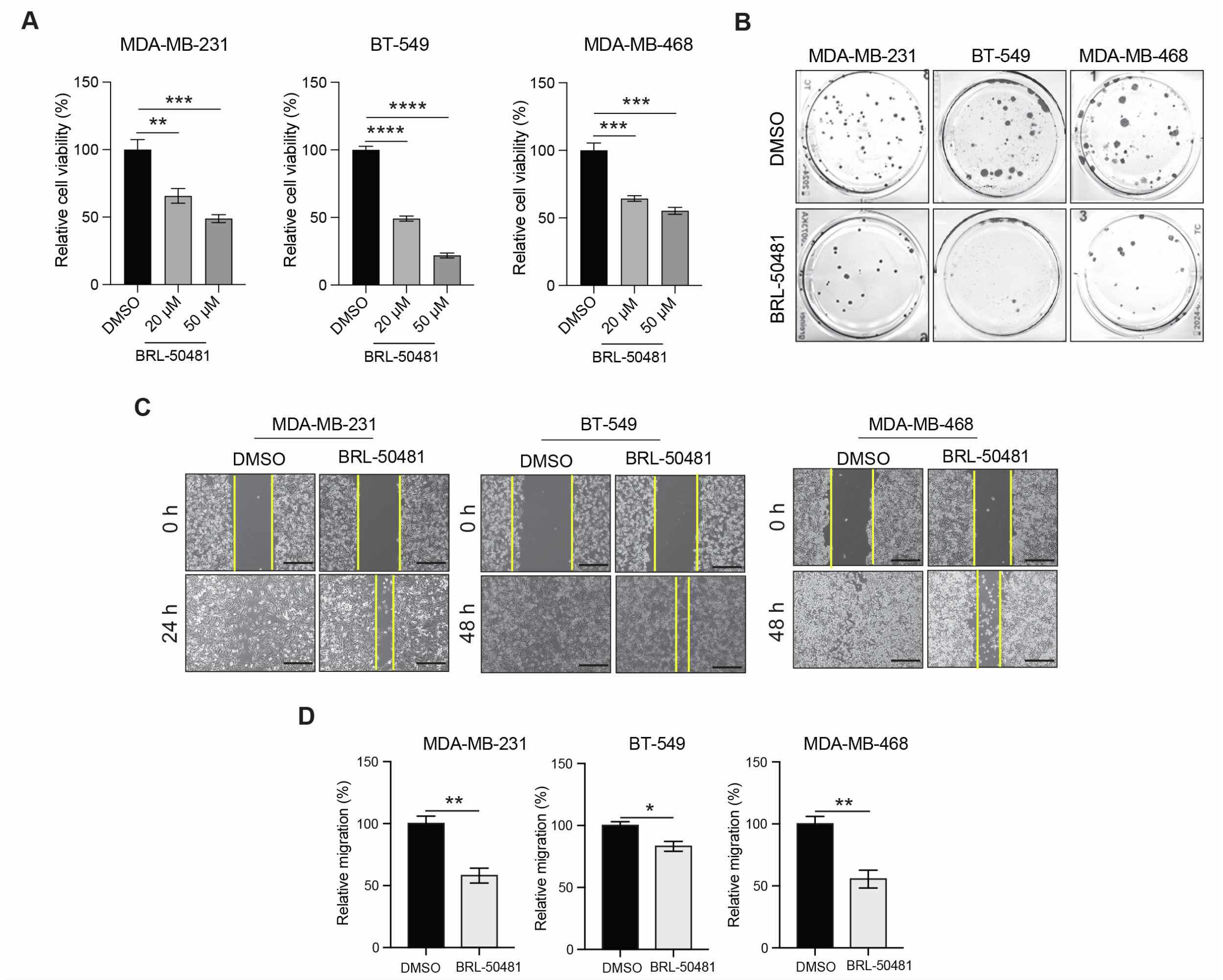

Figure S9. Pharmacological inhibition of PDE7A suppresses growth and migration of TNBC cells. Related to Figure 3. A. The indicated TNBC cell lines were treated with DMSO or BRL-50481 (20 or 50 μM) for 72 h. Cell viability was analyzed using an MTT assay and is presented relative to DMSO-treated cells (n = 4 biological replicates/group). **B.** Representative images of clonogenic assays to measure cell survival for the indicated TNBC cell lines under DMSO or BRL-50481 (50 μM) treatment conditions. **C.** The indicated TNBC cell lines were treated with DMSO or BRL-50481 (50 μM) and analyzed for migration using a wound-healing assay. Representative images are shown. Scale bar, 200 μm. **D.** Relative migration (%) is plotted relative to the DMSO-treated condition for the experiment shown in panel C (n = 3 biological replicates/group). All quantitative data represent the mean ± SEM. **p*<0.05, ***p*<0.01, ****p*<0.001, *****p*<0.0001.

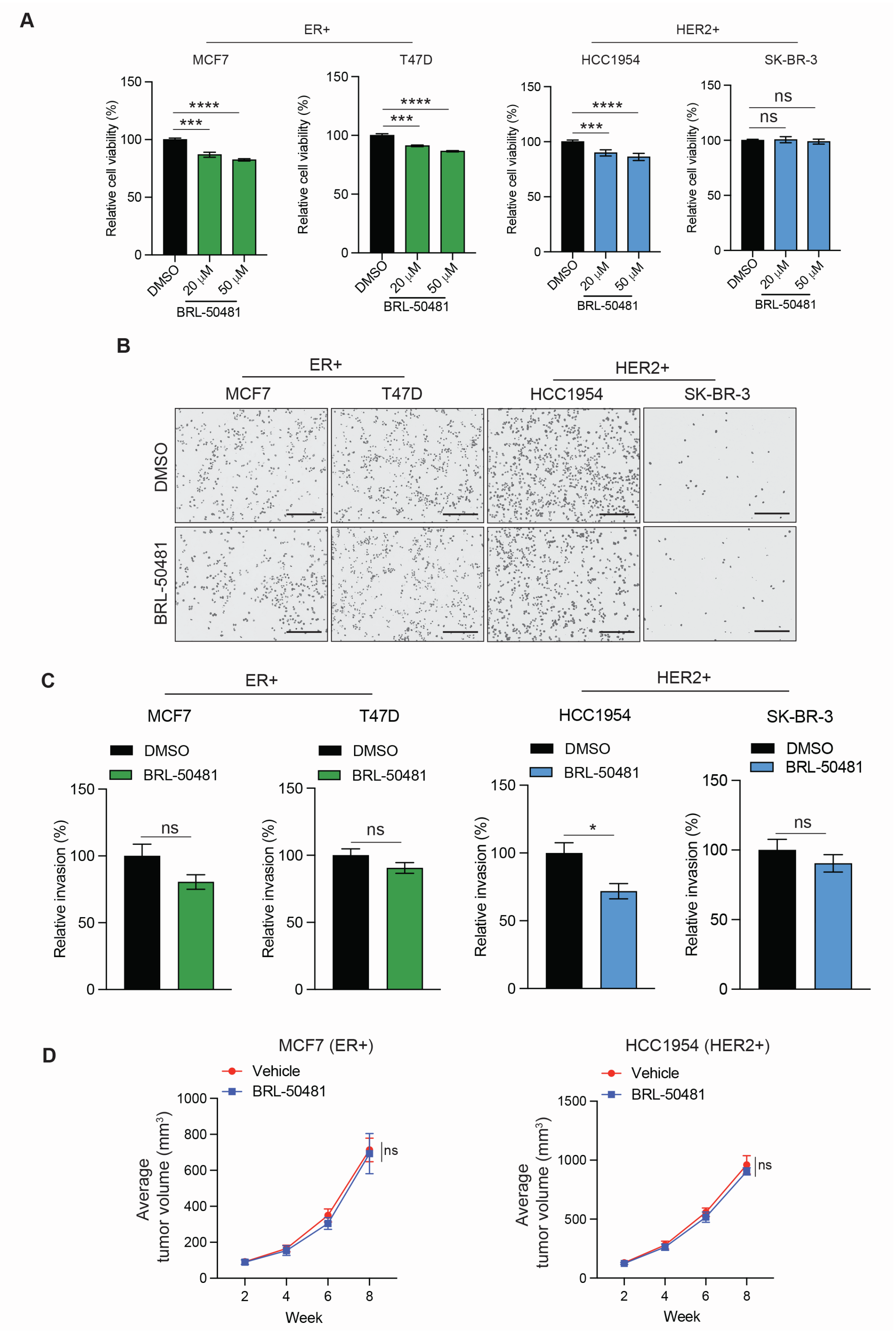
A
ER+
HER2+
MCF7
T47D
HCC1954
SK-BR-3
Relative cell viability (%)
DMSO
20 μM
50 μM
BRL-50481
****
***
ns
B
ER+
HER2+
MCF7
T47D
HCC1954
SK-BR-3
DMSO
BRL-50481
C
ER+
HER2+
MCF7
T47D
HCC1954
SK-BR-3
DMSO
BRL-50481
Relative invasion (%)
ns
*
D
MCF7 (ER+)
HCC1954 (HER2+)
Vehicle
BRL-50481
Average tumor volume (mm³)
Week
ns

Figure S10. PDE7A inhibition does not impact non-TNBC growth and invasion in cell culture and tumor growth in mice. Related to Figure 3. A. The indicated breast cancer cell lines from the indicated subtypes were treated with DMSO or BRL-50481 (20 or 50 μM) for 72 h. Cell survival was measured using the MTT assay and is presented relative to that of DMSO-treated cells (n = 5 biological replicates/group). **B.** The indicated breast cancer cell lines from the indicated subtypes were treated with DMSO or BRL-50481 (50 μM) for 20 h, and analyzed for invasive capacity using a Matrigel-based invasion assay. Representative images are shown. Scale bar: 200 μm. **C.** Relative invasion (%) compared to the DMSO-treated cells is plotted for the experiment shown in panel B (n = 3 biological replicates/group). **D.** MCF7 and HCC1954 cells were injected subcutaneously into the flanks of female NSG mice (n = 5/group, each cell line). Mice were treated every alternate day with the vehicle or BRL-50481 (25 mg/kg) intraperitoneally and tumor growth was measured. Average tumor volumes at the indicated time points under the indicated conditions are plotted. All quantitative data represent the mean ± SEM. ns= not significant. All quantitative data represent the mean ± SEM. ns= not significant, *p<0.05, ***p<0.001 and ****p<0.0001.

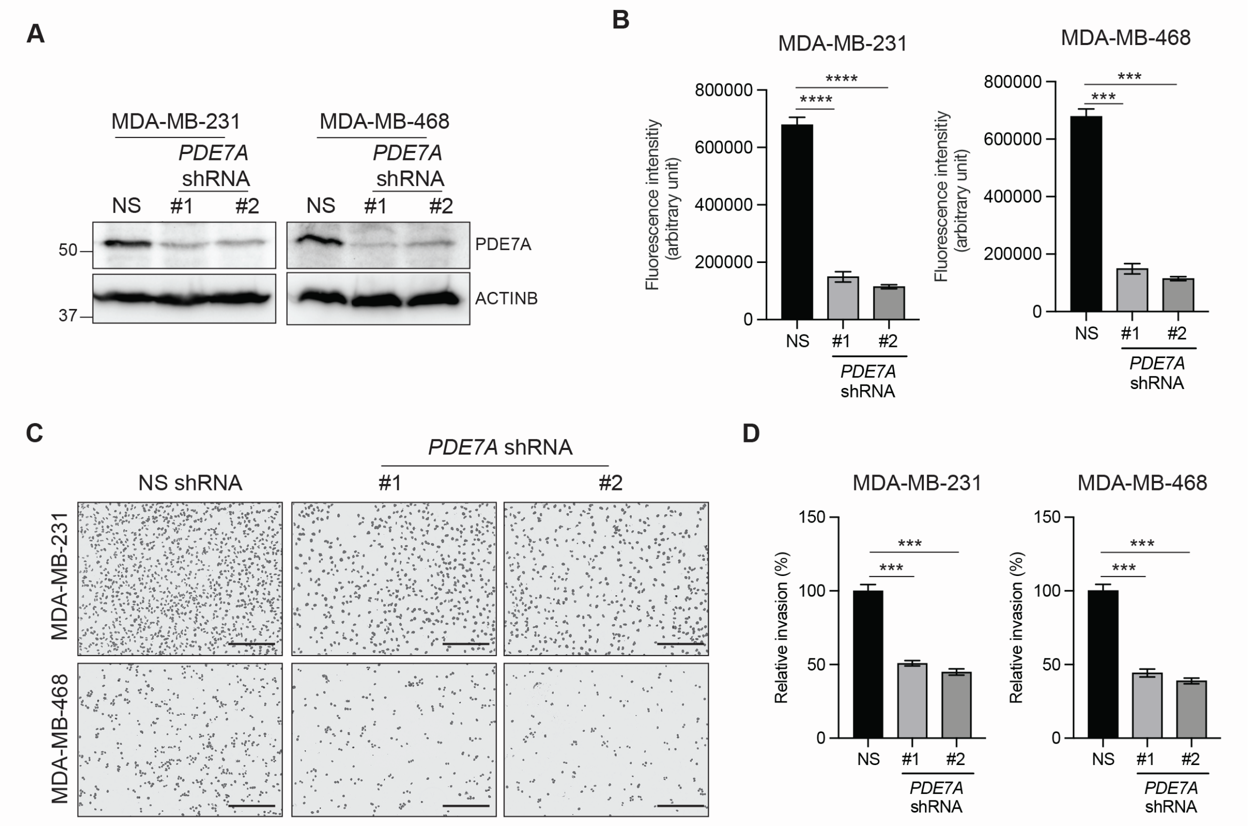

Figure S11. PDE7A knockdown inhibits TNBC growth and invasion. Related to Figure 3. A. The indicated TNBC cell lines expressing either non-specific (NS) shRNA or *PDE7A* shRNAs were analyzed for PDE7A protein expression by immunoblotting. ACTINB was used as a loading control. **B.** The indicated TNBC cell lines expressing either NS shRNA or *PDE7A* shRNAs were analyzed using the CytoSelect 96-well quantitative soft agar assay kit. Fluorescence intensities (arbitrary unit) under the indicated conditions are shown (n = 3 biological replicates/group). **C.** The indicated TNBC cell lines expressing either NS shRNA or *PDE7A* shRNAs were analyzed for invasive capacity using a Matrigel-based invasion assay. Representative images are shown. Scale bar, 200 µm. **D.** Relative invasion (%) relative to the NS shRNA-expressing cells is plotted for the experiment shown in panel C (n = 3 biological replicates/group). All quantitative data represent the mean ± SEM. ***$p < 0.001$, ****$p < 0.0001$.

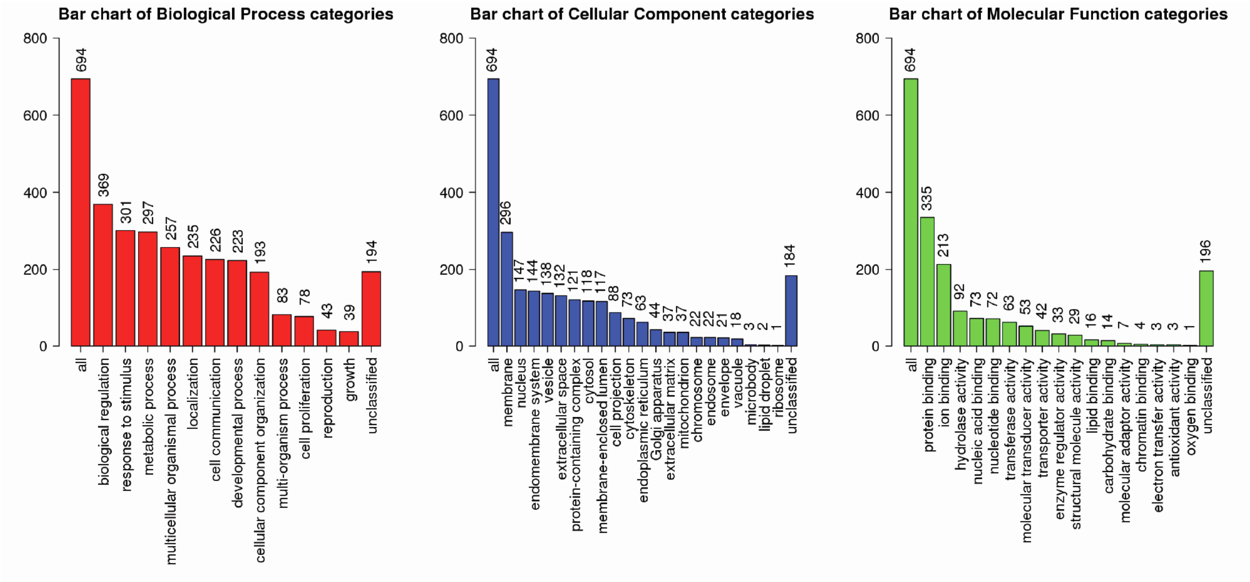

Figure S12. PDE7A inhibition suppresses TNBC cell growth. Related to Figure 4. Biological pathways that were altered in MDA-MB-231 cells treated with the phosphodiesterase 7A (PDE7A) inhibitor BRL-50481 (50 μM) for 72 h compared with DMSO-treated cells based on the mRNA expression profiles identified from RNA-sequencing results.

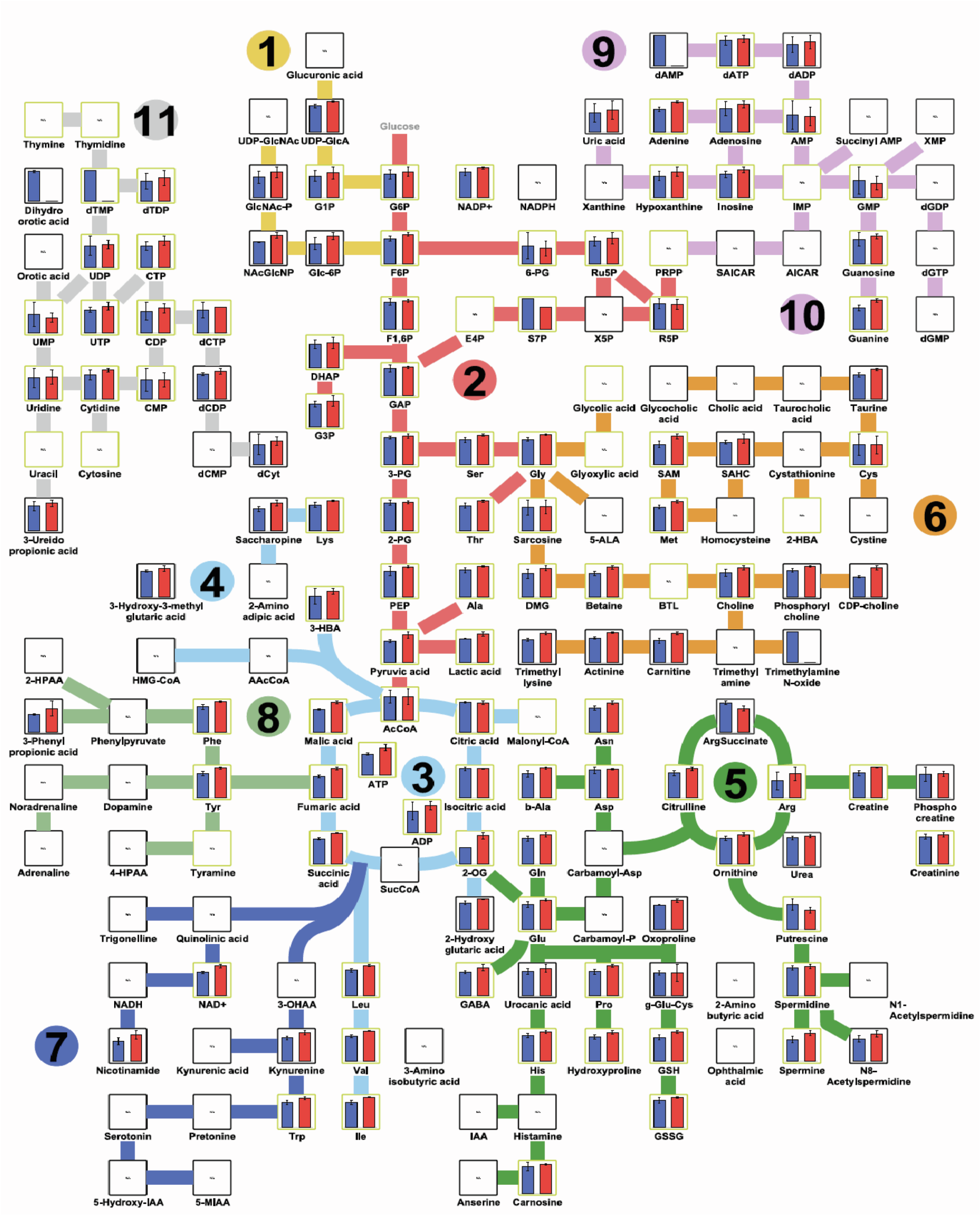

Figure S13. Pharmacological inhibition of PDE7A alters several metabolic pathways and metabolites. Related to Figure 4. Metabolic pathways and metabolites altered in MDA-MB-231 cells treated with the phosphodiesterase 7A (PDE7A) inhibitor BRL-50481 (50 µM) for 72 h

compared with control DMSO-treated cells based on the metabolite profiles from the metabolomics analysis.

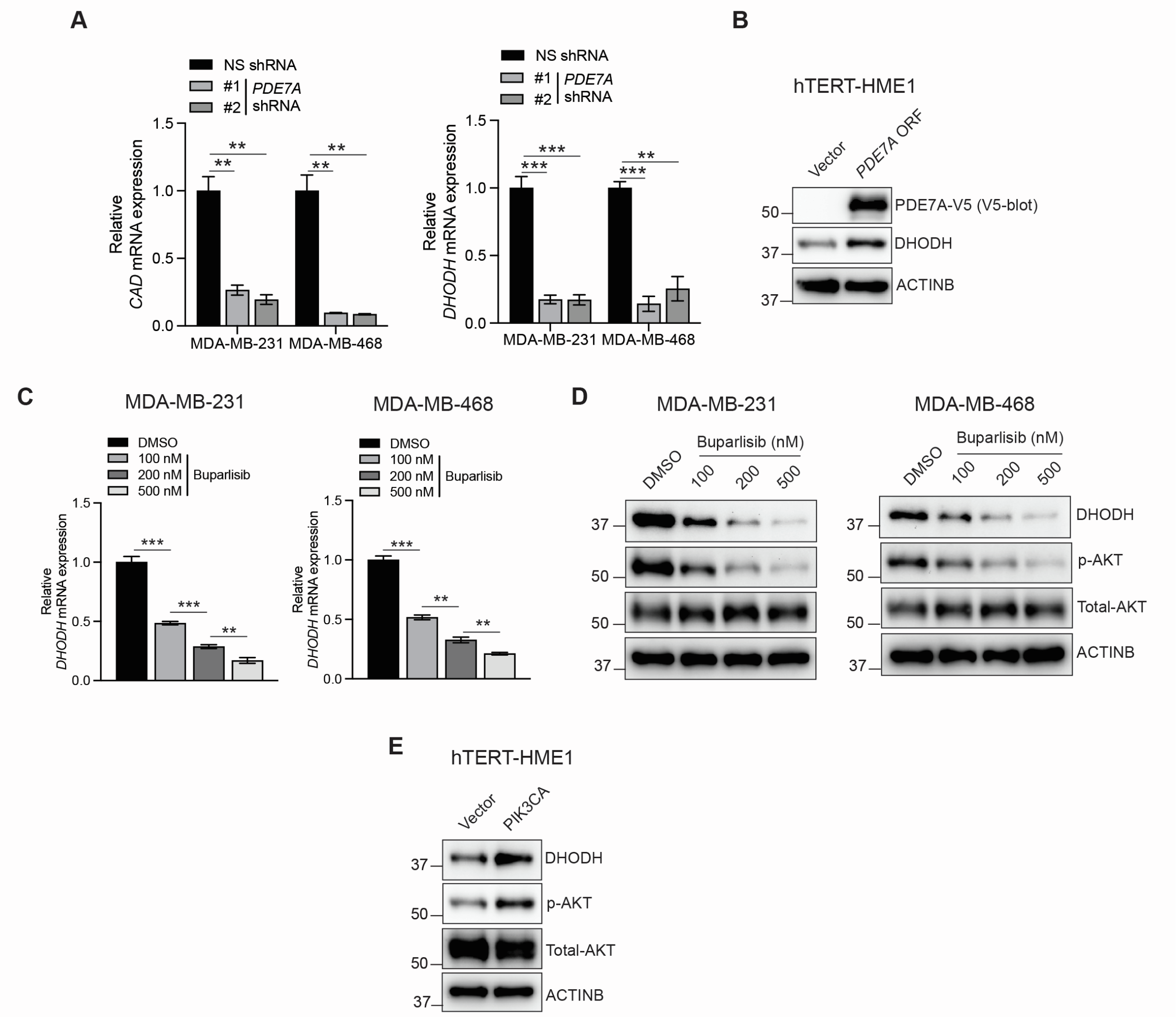

Figure S14. Impact of PDE7A knockdown on de novo pyrimidine biosynthesis genes and role of PI3K pathway in stimulating DHODH expression. Related to Figures 4 and 5. A. The indicated TNBC cell lines expressing non-specific (NS) shRNA or *PDE7A* shRNAs were analyzed for the expression of *DHODH* and *CAD* mRNA using RT-qPCR analysis (n = 3 biological replicates/group). Relative mRNA expression for *DHODH* and *CAD* in the indicated TNBC cell lines under the indicated conditions is shown. **B.** Immunoblot analysis of the expression of the indicated proteins in V5-tagged *PDE7A* ORF-expressing hTERT-HME1 cells under the indicated conditions. ACTINB was used as a loading control. **C.** The indicated TNBC cell lines were treated with either DMSO or the PI3K inhibitor buparlisib at the indicated concentrations for 48 h. *DHODH* mRNA expression was measured using RT-qPCR under the indicated conditions (n = 3 biological replicates/group). *ACTINB* was used as a normalization control. **D.** The indicated TNBC cell lines were treated with either DMSO or the PI3K inhibitor buparlisib at the indicated concentrations for 48 h. DHODH protein expression was measured using immunoblot analysis under the indicated conditions. ACTINB was used as a loading control. **E.** Immunoblot analysis of the expression of the indicated proteins in empty-vector or constitutively active PIK3CA-

expressing hTERT-HME1 cells under the indicated conditions. ACTINB was used as a loading control. All quantitative data represent the mean ± SEM. **$p<0.01$ and ***$p<0.001$.

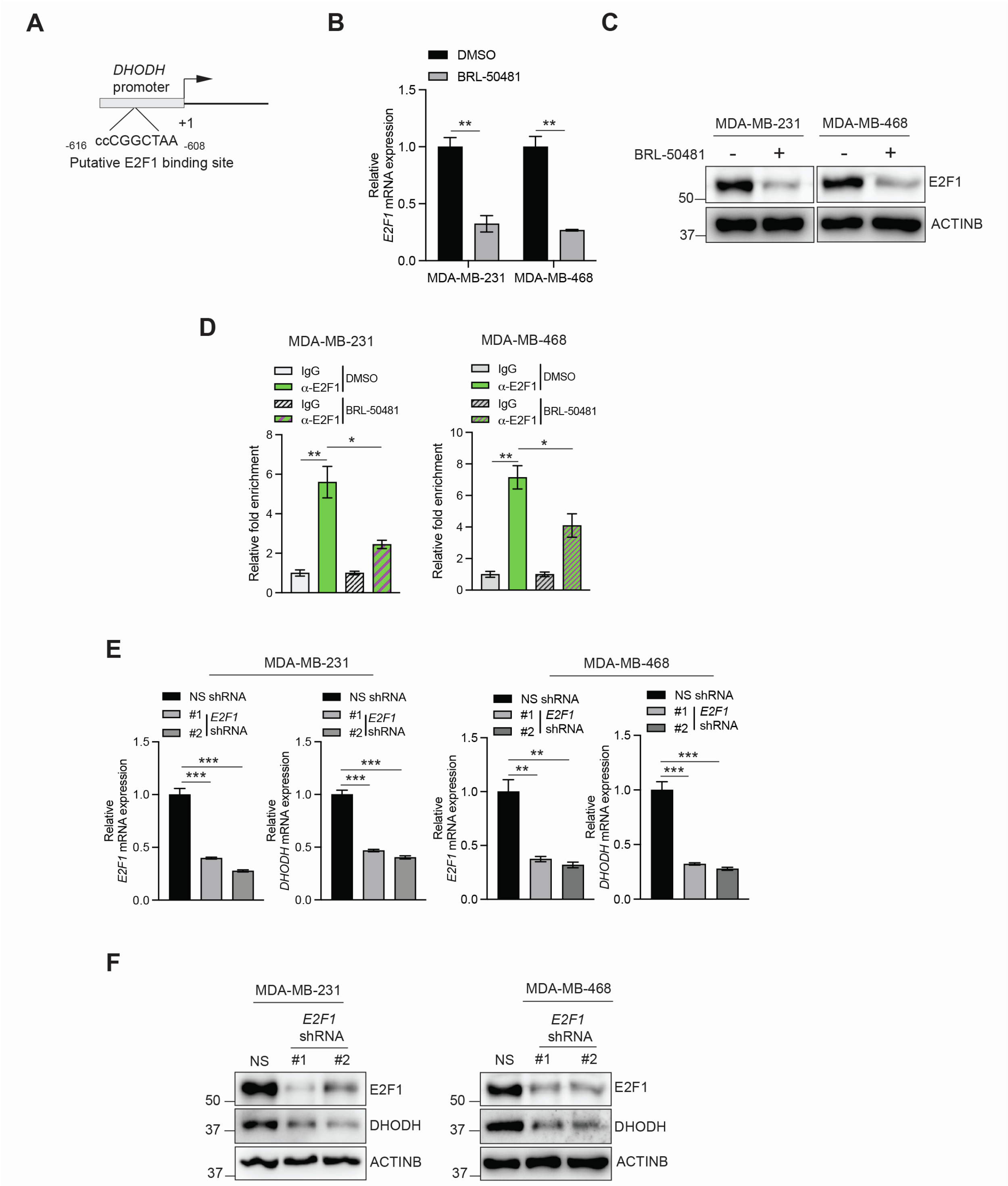

Figure S15. PDE7A via E2F1 stimulates the expression of DHODH in TNBC. Related to Figure 5. A. A schematic showing the E2F1 binding site on the *DHODH* promoter. **B.** The indicated TNBC cell lines were treated with either DMSO or BRL-50481 (50 μM) for 72 h. *E2F1* mRNA expression was measured by RT-qPCR. *E2F1* mRNA expression is plotted relative to DMSO-treated cells (n = 3 biological replicates/group). ACTINB was used as a normalization control. **C.** The indicated TNBC cell lines were treated with either DMSO or BRL-50481 (50 μM)

for 72 h. E2F1 protein expression was measured using immunoblot analysis under the indicated conditions. ACTINB was used as a loading control. **D.** The indicated TNBC cell lines were treated with either DMSO or BRL-50481 (50 μM) for 72 h, and were analyzed for assessing E2F1 binding on the *DHODH* promoter using CUT-&-RUN assay (n = 3 biological replicates/group). The ACTINB promoter was used as a control. IgG was used as a negative control for CUT-&-RUN assay, and fold-enrichment plotted relative to IgG under the indicated conditions is shown. **E.** The indicated TNBC cell lines expressing either non-specific (NS) shRNA or shRNAs targeting *E2F1* were analyzed for *E2F1* and *DHODH* mRNA expression using RT-qPCR. *E2F1* and *DHODH* mRNA expression is plotted relative to NS shRNA expressing cells (n = 3 biological replicates/group). ACTINB was used as a normalization control. **F.** The indicated TNBC cell lines expressing NS shRNA or *E2F1* shRNAs were analyzed for the expression of the indicated proteins by immunoblotting. ACTINB was used as a loading control. All quantitative data represent the mean ± SEM. *$p<0.05$, **$p<0.01$, ***$p<0.001$.

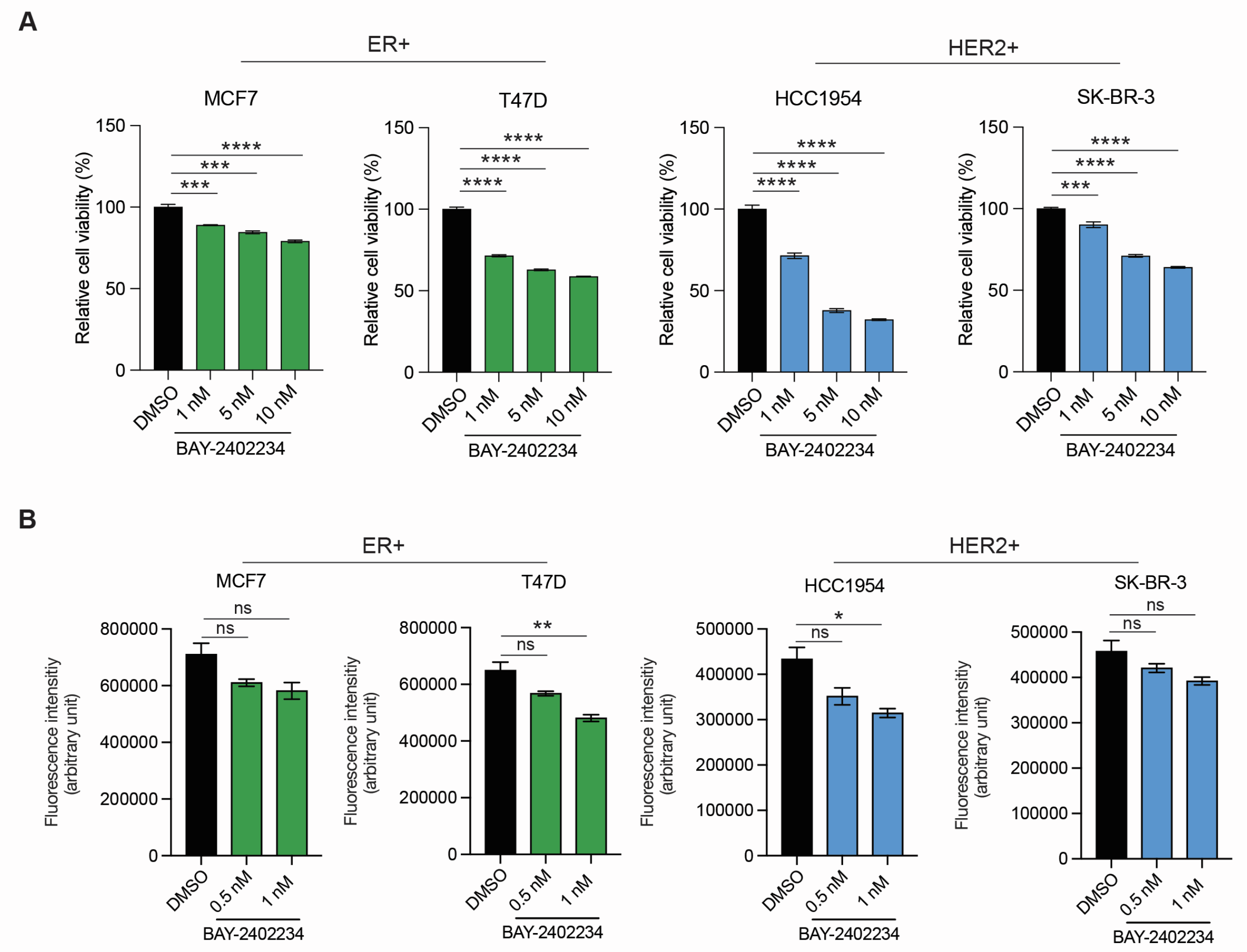

Figure S16. DHODH inhibition produces variable but largely marginal effect on the growth of non-TNBC cell lines. Related to Figure 5. A. The indicated breast cancer cell lines from the indicated subtypes were treated with DMSO or the DHODH inhibitor BAY-2402234 at the indicated concentrations for 72 h and analyzed for cell viability using the MTT assay. Relative cell viability is plotted compared to DMSO-treated cells (n = 5 biological replicates/group). **B.** The indicated breast cancer cell lines from the indicated breast cancer subtypes were treated with DMSO or BAY-2402234 at the indicated concentrations, and a quantitative soft-agar assay was performed using the CytoSelect 96-well quantitative soft agar assay. Fluorescence intensities (arbitrary unit) under the indicated conditions for the indicated breast cancer cell lines from the indicated breast cancer subtypes are shown (n = 3 biological replicates/group). All quantitative data represent the mean ± SEM. ns= not significant, *p<0.05, **p<0.01, ***p<0.001 and ****p<0.0001.

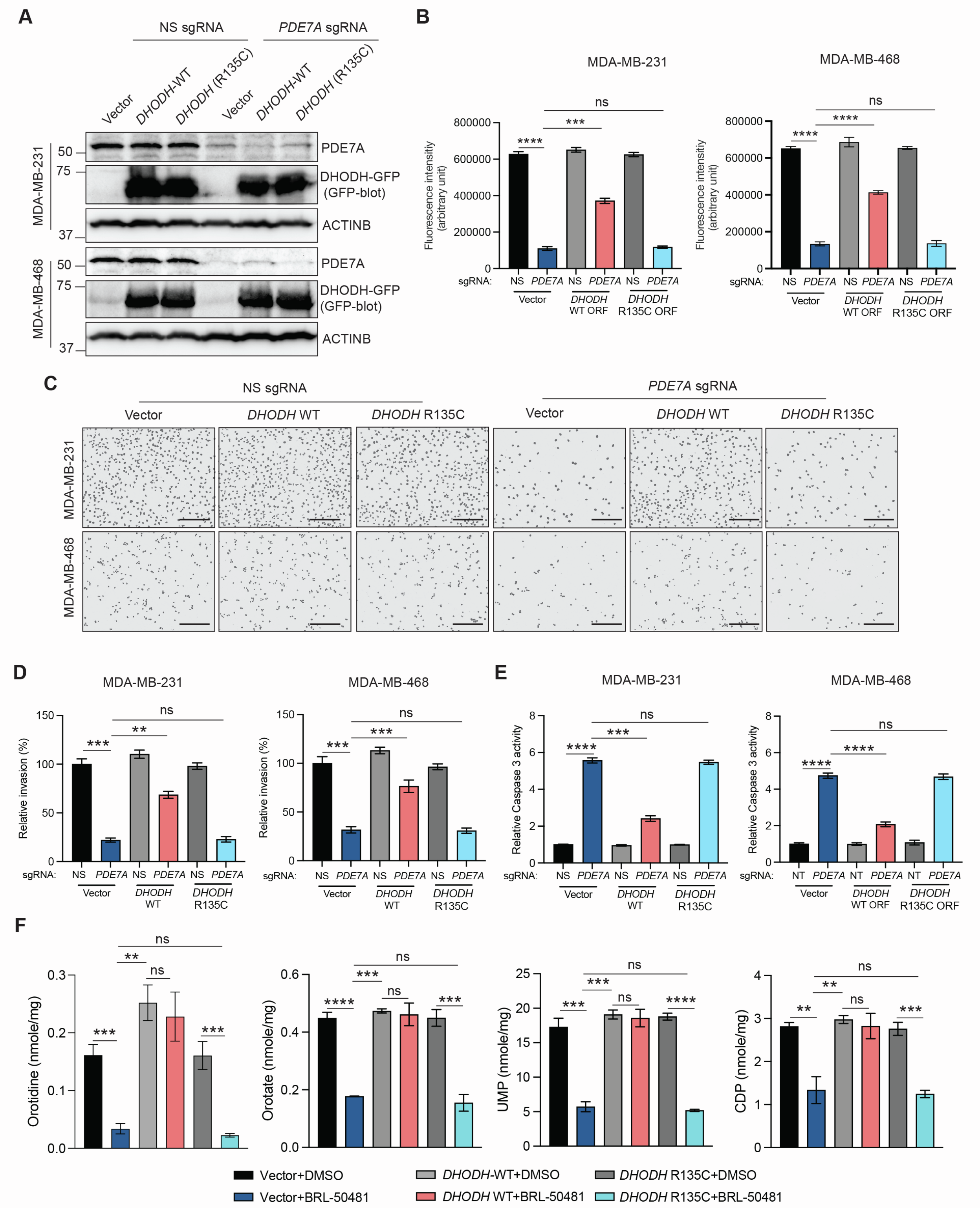

Figure S17. Enzymatic activity defective mutant DHODH (R135C), unlike enzymatic activity proficient wild-type DHODH fails to rescue PDE7A-loss driven phenotypes in TNBC cells. Related to Figure 6. A. The indicated TNBC cell lines expressing an empty vector, wild-type (WT) *DHODH* or catalytic mutant (R135C) *DHODH* ORF and simultaneously expressing non-

specific (NS) sgRNA or *PDE7A*-targeting sgRNA were analyzed for expression of the indicated proteins. ACTINB was used as the loading control. **B.** The indicated TNBC cell lines expressing an empty vector, wild-type (WT) *DHODH* or catalytic mutant (R135C) *DHODH* ORF and simultaneously expressing NS sgRNA or *PDE7A*-targeting sgRNA were analyzed using the CytoSelect 96-well quantitative soft agar assay kit. Fluorescence intensities (arbitrary unit) under the indicated conditions are shown (n = 3 biological replicates/group). **C.** The indicated TNBC cell lines expressing an empty vector, wild-type (WT) *DHODH* or catalytic mutant (R135C) *DHODH* ORF and simultaneously expressing NS sgRNA or *PDE7A*-targeting sgRNA were analyzed for invasive capacity using a Matrigel-based invasion assay. Representative images for the indicated cell lines under the indicated conditions are shown. Scale bar, 200 µm. **D.** Relative invasion (%) for the indicated cell lines under the indicated conditions is plotted for the experiment shown in panel C (n = 3 biological replicates/group). **E.** The indicated TNBC cell lines expressing an empty vector, wild-type (WT) *DHODH* or catalytic mutant (R135C) *DHODH* ORF and simultaneously expressing NS sgRNA or *PDE7A*-targeting sgRNA were analyzed for caspase-3 activity using a caspase 3-based colorimetric assay. Relative caspase-3 activity for the indicated cell lines under the indicated conditions is shown (n = 3 biological replicates/group). **F.** MDA-MB-231 cells expressing an empty vector, wild-type (WT) *DHODH* or catalytic mutant (R135C) *DHODH* ORF were treated with DMSO or BRL-50481 (50 µM) for 72 h, and were analyzed for the indicated metabolites. Indicated metabolites are presented in nmole/mg for the indicated conditions (n = 3 biological replicates/group). All quantitative data represent the mean ± SEM. ns= not significant, **p<0.01, ***p<0.001, ****p<0.0001.

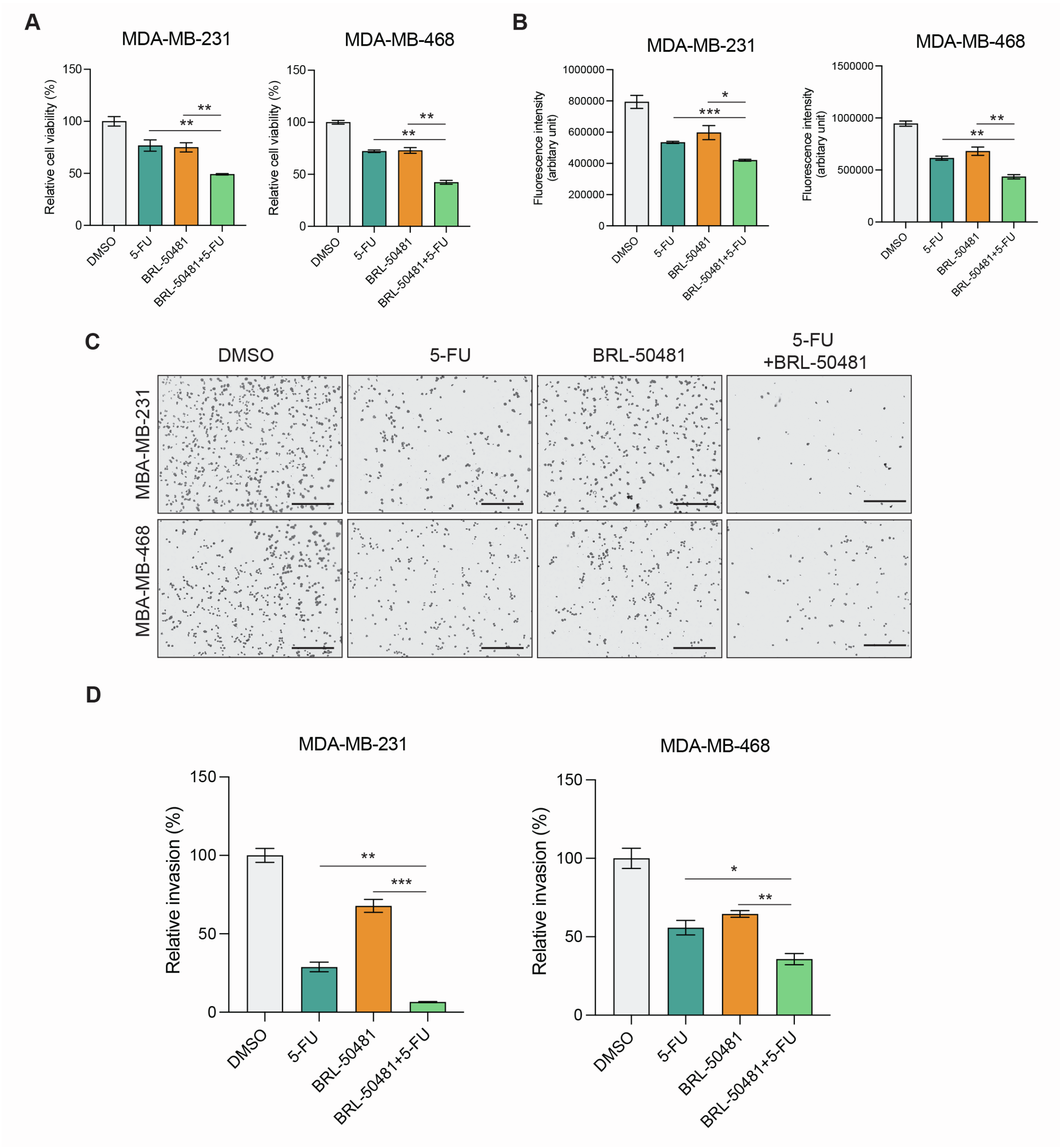

Figure S18. 5-FU enhances the growth inhibitory effect of BRL-50481 in TNBC cells. Related to Figure 6. A. The indicated TNBC cell lines were treated with DMSO, PDE7A inhibitor BRL-50481 (20 µM), 5-FU (10 µM) or with the combination of both BRL-50481 (20 µM) and 5-FU (10 µM) for 72 h, and were analyzed for cell viability using the MTT assay. Relative cell viability is plotted relative to DMSO-treated cells (n = 4 biological replicates/group). **B.** The indicated TNBC cell lines were treated with DMSO, PDE7A inhibitor BRL-50481 (20 µM), 5-FU (10 µM) or with the combination of both BRL-50481 (20 µM) and 5-FU (10 µM) and a quantitative soft-agar assay was performed using the CytoSelect 96-well quantitative soft agar assay. Fluorescence intensities (arbitrary unit) under the indicated conditions are shown (n = 3 biological

replicates/group). **C.** The indicated TNBC cell lines were treated with DMSO, PDE7A inhibitor BRL-50481 (20 μM), 5-FU (10 μM) or with the combination of both BRL-50481 (20 μM) and 5-FU (10 μM) for 20 h, and analyzed for invasive capacity using a Matrigel-based invasion assay. Representative images are shown. Scale bar, 200 μm. **D.** Relative invasion (%) relative to the DMSO-treated cells is plotted for the experiment shown in panel C (n = 3 biological replicates/group). All quantitative data represent the mean ± SEM. $^{*}p < 0.05$, $^{**}p < 0.01$, $^{***}p < 0.001$.
