# Supplementary material for: PDE7A inhibition suppresses triple-negative breast cancer by attenuating de novo pyrimidine biosynthesis
Source: Cell Rep Med. 2025 Sep 16;6(9):102356. doi: 10.1016/j.xcrm.2025.102356 (PMC12490241; doi:10.1016/j.xcrm.2025.102356)
Supplement: Document S2. Article plus supplemental information [file mmc6.pdf]

Cell Reports
Medicine

Article

PDE7A inhibition suppresses triple-negative breast cancer by attenuating *de novo* pyrimidine biosynthesis

Graphical abstract

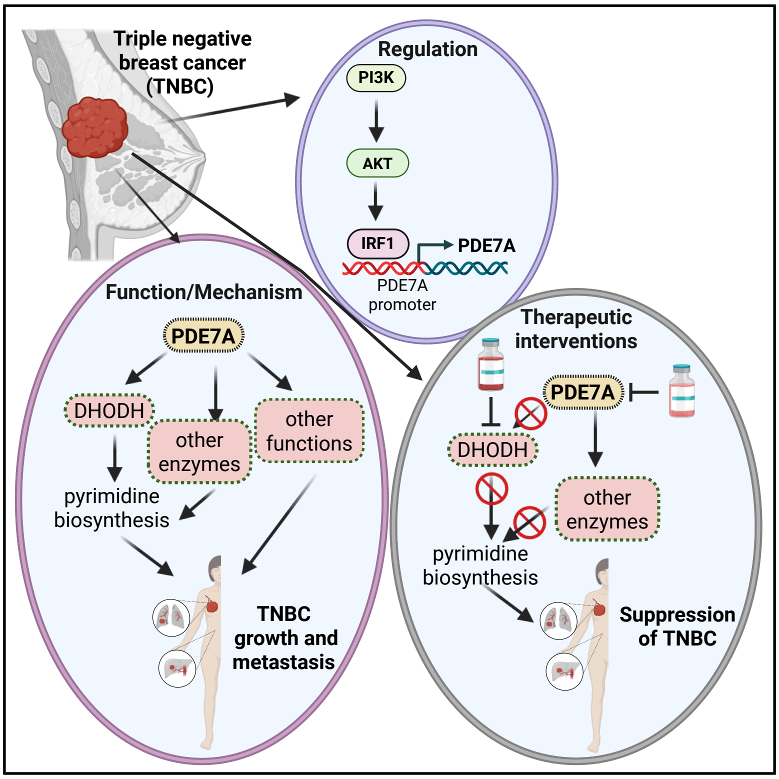

Highlights

- PDE7A is overexpressed in TNBC by the PI3K/AKT→IRF1 pathway
- PDE7A inhibition results in the suppression of TNBC tumor growth and metastasis
- PDE7A regulates *de novo* pyrimidine biosynthesis via DHODH to promote TNBC
- Combinatorial pharmacological targeting of PDE7A and DHODH potently inhibits TNBC

Authors

Parmanand Malvi, Suresh Bugide, Roshan Dutta, ..., Kamaljeet Singh, Romi Gupta, Narendra Wajapeyee

Correspondence

romigup@uab.edu (R.G.),
nwajapey@uab.edu (N.W.)

In brief

Malvi et al. identify PDE7A as a driver of triple-negative breast cancer (TNBC) tumor growth and metastasis. Inhibition of PDE7A attenuates *de novo* pyrimidine biosynthesis by repressing DHODH expression, causing suppression of TNBC tumor growth and metastasis. Furthermore, the combined pharmacological inhibition of PDE7A and DHODH effectively suppresses TNBC.

Malvi et al., 2025, Cell Reports Medicine *6*, 102356
September 16, 2025 © 2025 The Author(s). Published by Elsevier Inc.
https://doi.org/10.1016/j.xcrm.2025.102356

CellPress

Cell Reports Medicine

CellPress
OPEN ACCESS

Article

PDE7A inhibition suppresses triple-negative breast cancer by attenuating *de novo* pyrimidine biosynthesis

Parmanand Malvi,[1,4] Suresh Bugide,[1,4] Roshan Dutta,[1] Kiran Kumar Reddi,[1] Yvonne J.K. Edwards,[1] Kamaljeet Singh,[2] Romi Gupta,[1,3,*] and Narendra Wajapeyee[1,3,5,*]
[1]Department of Biochemistry and Molecular Genetics, The University of Alabama at Birmingham, Birmingham, AL 35233, USA
[2]Department of Pathology and Laboratory Medicine, Brown University, Providence, RI 02912, USA
[3]O'Neal Comprehensive Cancer Center at The University of Alabama at Birmingham, Birmingham, AL 35233, USA
[4]These authors contributed equally
[5]Lead contact
*Correspondence: romigup@uab.edu (R.G.), nwajapey@uab.edu (N.W.)
https://doi.org/10.1016/j.xcrm.2025.102356

SUMMARY

Triple-negative breast cancer (TNBC) is an aggressive subtype of breast cancer, associated with poor response to therapies and high mortality. We identify that phosphodiesterase 7A (PDE7A) is overexpressed in the majority of TNBCs, and a higher level of PDE7A associates with poor prognosis. The phosphatidylinositol 3-kinase (PI3K)/AKT pathway, via the transcription factor IRF1, stimulates the expression of PDE7A in TNBC cells. PDE7A inhibition attenuates TNBC growth in both cell culture and mouse models of TNBC. Inhibition of PDE7A suppresses *de novo* pyrimidine biosynthesis, in part through the downregulation of the enzyme dihydroorotate dehydrogenase (DHODH). DHODH suppression attenuates TNBC tumor growth, mirroring the effects of PDE7A inhibition, and ectopic DHODH expression rescues PDE7A-inhibition-induced tumor suppression. Pharmacological co-targeting of PDE7A and DHODH potently inhibits TNBC tumor growth and metastasis. These findings identify the PDE7A → DHODH → *de novo* pyrimidine biosynthesis pathway as a key driver of TNBC, offering additional therapeutic opportunities for TNBC patients.

INTRODUCTION

Breast cancer is one of the most commonly diagnosed cancers in women and a leading cause of cancer-related mortality.[1–3] The triple-negative breast cancer (TNBC) subtype represents a particularly aggressive form, characterized by the absence of estrogen receptors, progesterone receptors, and human epidermal growth factor receptor 2 (HER2) expression.[4,5] TNBC accounts for approximately 10%–15% of all breast cancer cases and displays higher probability of metastasis, poorer prognosis, and distinct patterns of recurrence.[4–6] Current therapeutic strategies for TNBC primarily involve a combination of surgery, radiation therapy, and chemotherapy.[5,7] Recent therapeutic advances have also introduced the use of immunotherapy and Poly (ADP-ribose) polymerase (PARP) inhibitors for certain TNBC patients.[8,9] Despite these developments, the search for more effective treatments remains imperative, as TNBC continues to exhibit lower survival rates compared to other breast cancer subtypes.

Cancer cells exhibit unique metabolic dependencies that distinguish them from their normal counterparts, presenting a vulnerability that can be exploited by targeted therapies.[10–12] This metabolic reprogramming is integral to tumor development and progression[13–15] as well as in determining the response to anticancer agents.[16] The identification of key metabolic enzymes and pathways that are essential for cancer cell survival has paved the way for innovative therapeutic strategies. Specifically, therapies designed to target the metabolic vulnerabilities of cancer cells are being developed, with several already advancing to clinical trials.[11,17]

Cyclic nucleotides serve as second messengers, mediating various cellular responses to extracellular signals, such as hormones, light, or neurotransmitters.[18,19] Cyclic nucleotide phosphodiesterases (PDEs) regulate the cellular concentrations of cyclic nucleotides, which result in modulation of various signal transduction pathways and transcription factors.[20–22] Mammalian cells express multiple PDEs belonging to at least 11 families, categorized according to substrate affinity and selective sensitivity to cofactors and inhibitory drugs.[23–25] Phosphodiesterase 7A (PDE7A) belongs to the PDE7 subfamily[26] and hydrolyzes the second messenger 3′,5′-cyclic adenosine monophosphate (cAMP),[27] which plays a role in many biological processes, including cancer growth, migration, and invasion; cell-cycle regulation; transcriptional activation; and apoptosis.[28–30] However, the role of PDE7A in TNBC and whether targeting PDE7A has therapeutic value in TNBC remain to be determined. Here, we identify the PDE7A → dihydroorotate dehydrogenase (DHODH) → *de novo*

© 2025 The Author(s). Published by Elsevier Inc.
This is an open access article under the CC BY license (http://creativecommons.org/licenses/by/4.0/).

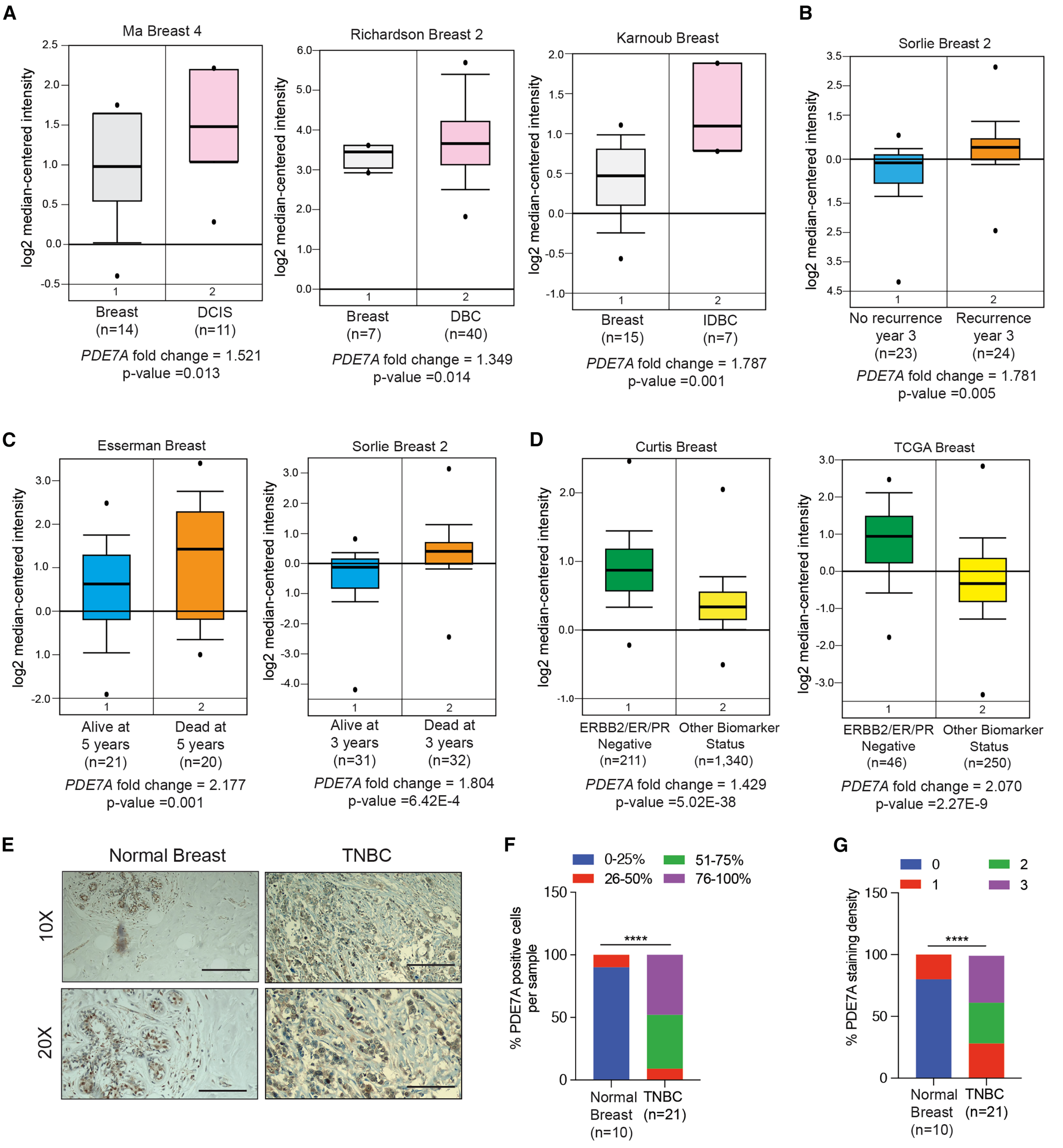

Figure 1. PDE7A is overexpressed in TNBC samples

(A) *PDE7A* mRNA expression in breast cancer samples compared to normal breast tissues in the indicated datasets. Fold changes and *p* values are shown.

(B) *PDE7A* mRNA expression levels are compared between breast cancer samples with and without recurrence at 3 years in the indicated dataset. Fold changes and *p* values for are shown.

(C) *PDE7A* mRNA expression levels in breast cancer samples from patients who are alive or dead at the indicated years in the indicated datasets. Fold changes and *p* values are shown.

(D) *PDE7A* mRNA expression levels between triple-negative breast cancer (TNBC) and other breast cancer subtypes in the indicated datasets. Fold changes and *p* values are shown.

(legend continued on next page)

pyrimidine biosynthesis pathway as a key driver of TNBC and as a therapeutic target for the treatment of TNBC.

RESULTS

PDE7A is overexpressed in TNBC by the action of the phosphatidylinositol 3-kinase-AKT-IRF1 pathway

Cancer is a complex disease driven by multiple distinct genetic and epigenetic alterations[31]; therefore, identifying the factors required for tumor growth and metastasis is likely to reveal cancer-specific liabilities that can be therapeutically targeted. To identify such cancer-specific liabilities, we analyzed patient-derived ductal breast carcinoma samples for the expression of PDEs and identified significant overexpression of *PDE7A* mRNA in patient-derived ductal breast carcinoma samples compared to normal breast tissues (Figures 1A and S1A). Furthermore, *PDE7A* overexpression in ductal breast carcinoma samples was associated with an increased incidence of disease recurrence (Figure 1B) and reduced overall survival (Figure 1C) in breast cancer patients. We also observed that higher *PDE7A* mRNA levels were more strongly associated with TNBC than with other breast cancer subtypes (Figures 1D and S1B). Consistent with this, human TNBC cell lines expressed higher levels of PDE7A compared to non-transformed mammary epithelial cells (hTERT-HME1) or non-TNBC cell lines (Figure S1C). Analysis of other PDEs showed no upregulation in TNBC samples compared to normal breast samples, except for PDE6A (Figure S2). However, PDE6A knockdown did not suppress TNBC tumor cell growth or invasion (Figure S3), demonstrating that PDE6A does not play a functional role in TNBC.

Thus, we focused on PDE7A and measured the PDE7A protein levels in TNBC samples by performing an immunohistochemistry (IHC) using a breast cancer tissue microarray (US Biomax: BC081120f) consisting of 110 cases of invasive carcinoma (including 21 TNBC cases) and 10 samples of adjacent normal breast tissue. Consistent with the observed overexpression of PDE7A mRNA in TNBC patients, significantly higher levels of PDE7A protein were observed in the majority of TNBC samples compared to adjacent normal breast tissues (Figures 1E–1G; Table S1). Collectively, these results demonstrate that PDE7A is overexpressed in TNBC.

Because our results showed *PDE7A* overexpression in TNBC at the mRNA level, we investigated the mechanism driving *PDE7A* transcription in TNBC. A common feature of TNBC is the phosphatidylinositol 3-kinase (PI3K)-AKT pathway activation due to phosphatidylinositol-4,5-bisphosphate 3-kinase, catalytic subunit alpha (PI3KCA) mutations or amplifications, or the loss of phosphatase and tensin homolog or inositol polyphosphate-4-phosphatase type II B.[32,33] Therefore, we examined whether PI3K-AKT activation is involved in the upregulation of PDE7A expression in TNBC. We measured PDE7A expression in TNBC cell lines (MDA-MB-231 and MDA-MB-468) treated with the PI3K inhibitor buparlisib[34] and found that buparlisib treatment reduced PDE7A expression at both the mRNA and protein levels in a dose-dependent manner (Figures 2A and 2B). To further strengthen the role of the PI3K/AKT pathway in stimulating the expression of PDE7A, we ectopically expressed constitutively active PIK3CA[35] in hTERT-HME1 and measured p-AKT levels and PDE7A expression. Consistent with the role of the PI3K/AKT pathway in stimulating the expression of PDE7A, constitutively active PIK3CA increased the expression level of PDE7A in hTERT-HME1 cells (Figure S4A). We also tested the role of p53, which is frequently inactivated due to the mutations in TNBC, by ectopically expressing p53 hTERT-HME1 cells and measuring PDE7A levels. We found that p53 ectopic expression did not influence PDE7A levels (Figure S4B). Similarly, unrelated growth suppressive drugs such as doxorubicin and etoposide, also had no impact on the levels of PDE7A in TNBC cells (Figure S4C). Collectively, these results demonstrate that the PI3K/AKT pathway promotes PDE7A upregulation in TNBC cells.

Next, we asked how the PI3K-AKT pathway stimulates PDE7A expression. We tested three major mechanisms by which mRNA and protein expression are typically regulated. These included transcriptional regulation, mRNA stability regulation, and protein stability regulation by ubiquitin-proteasome system. We treated TNBC cell lines (MDA-MB-231 and MDA-MB-468) with DMSO or buparlisib and tested the enrichment of RNA polymerase II with Ser2-phosphorylated C-terminal domain (CTD). Recruitment of RNA pol II CTD phospho Ser2 to gene bodies reflects productive elongation and is indicative of regulation at the transcription level.[36,37] Buparlisib-treated TNBC cells showed reduced Ser2-phosphorylated RNA polymerase II on the *PDE7A* gene body, which is indicative of reduced transcription following treatment with buparlisib (Figure S4D). However, no effect on *PDE7A* mRNA stability in the actinomycin D treatment-based mRNA half-life measurement was observed following treatment with buparlisib (Figure S4E). Similarly, treatment with proteasome inhibitor MG-132 following buparlisib treatment did not influence PDE7A protein level (Figure S4F), which rules out the role of proteasome-mediated degradation pathway in regulating PDE7A expression. Collectively, these results demonstrate that PDE7A in TNBC cells is primarily regulated at the level of transcription.

Therefore, we focused on transcription regulation as a primary mechanism for PDE7A overexpression in TNBC cells. We first analyzed the 1 kb promoter region of the *PDE7A* gene using the PROMO search program.[38,39] We found putative consensus DNA-binding sites for 10 transcription factors on the *PDE7A* gene promoter (Figure 2C). Next, we asked if these transcription factors were altered in TNBC patient samples similar to PDE7A expression. We found that all 10 transcription factors were significantly altered (either downregulated or upregulated) in TNBC samples compared to normal breast samples (Figures 2D and S5). Next, we asked if the expression of these transcription

(E) PDE7A protein expression was measured by immunohistochemistry (IHC) staining in a tissue microarray (TMA) containing TNBC samples and adjacent normal breast tissues (10× and 20× magnification). Representative images are shown. Scale bar, 200 μm for 10× and 100 μm for 20×.
(F) % PDE7A-positive cells in the TMA for TNBC samples ($n = 21$) and adjacent normal breast tissues ($n = 10$) are shown.
(G) % PDE7A staining density in the TMA for TNBC samples ($n = 21$) and matched adjacent normal breast tissues ($n = 10$) are shown.
The p value was calculated using the chi-squared test. ****$p < 0.0001$. See also Figures S1–S3 and Table S1.

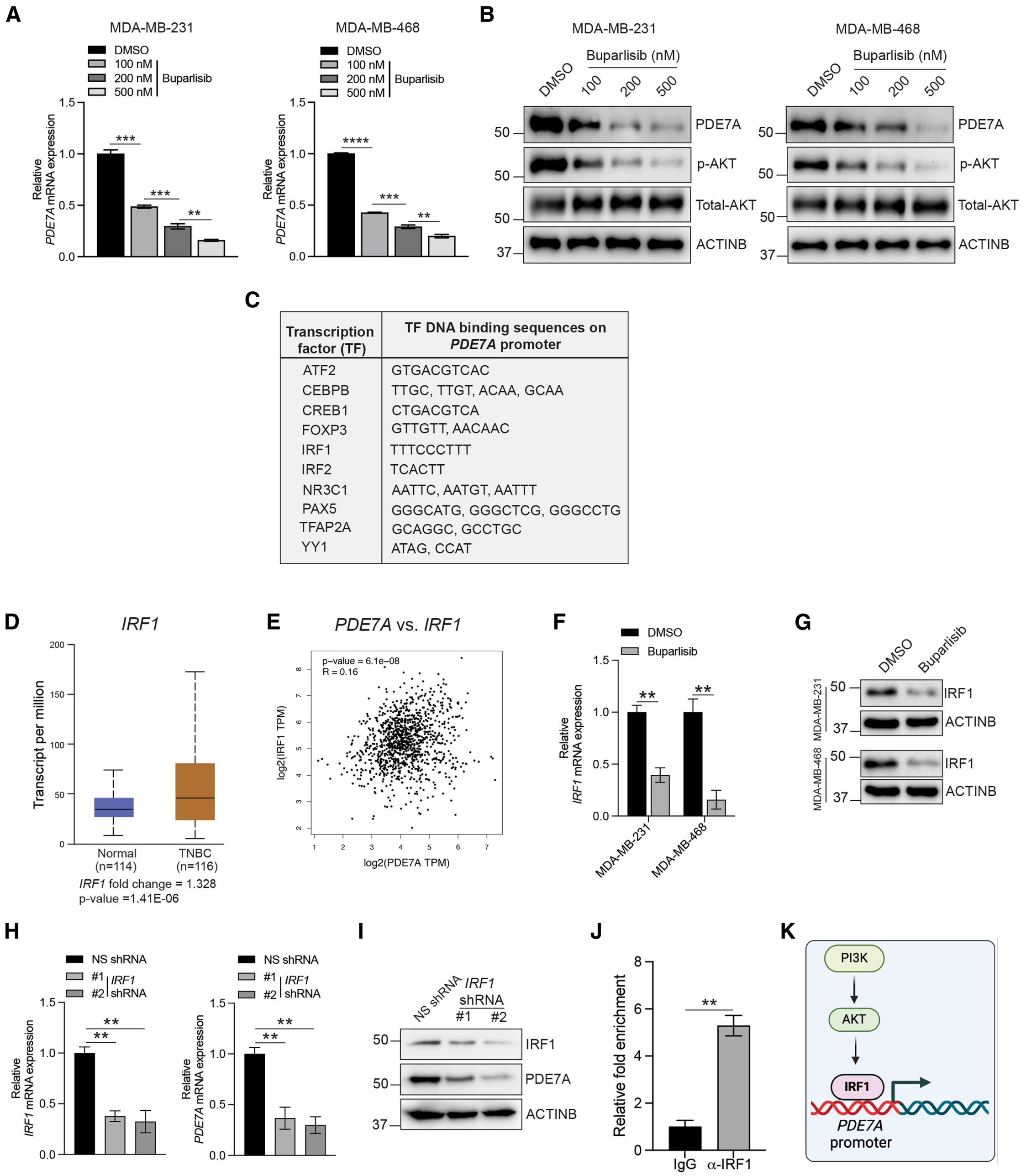

Figure 2. PI3K-AKT pathway stimulates the expression of PDE7A in TNBC cells via the transcription factor IRF1

(A) The indicated TNBC cell lines were treated with either DMSO or the PI3K inhibitor buparlisib at the indicated concentrations for 48 h. *PDE7A* mRNA expression was measured using quantitative reverse-transcription PCR (RT-qPCR) and is presented as the level in buparlisib-treated cells relative to that the in indicated DMSO-treated TNBC cells (n = 3 biological replicates/group). Actin beta (*ACTINB)* was used for normalization.

(B) The indicated TNBC cell lines were treated with either DMSO or the PI3K inhibitor buparlisib at the indicated concentrations for 48 h. PDE7A protein expression was measured using immunoblot analysis. ACTINB was used as a loading control.

(C) Transcription factors with predicted DNA binding sites on the *PDE7A* promoter DNA sequence (1 kb upstream from the transcription start site) generated using PROMO search.

(D) Using The University of Alabama at Birmingham CANcer data analysis Portal (UALCAN), *IRF1* mRNA expression in TNBC samples from The Cancer Genome Atlas (TCGA) breast cancer dataset was analyzed. The fold change and p value are shown.

(E) Correlation of *PDE7A* and *IRF1* mRNA expression in the TCGA breast cancer dataset analyzed using gene expression profiling interactive analysis (GEPIA). The dot plot, Pearson correlation coefficient (R), and p value are shown.

(F) *IRF1* mRNA expression was measured using quantitative reverse-transcription PCR (RT-qPCR) and is presented as the level in buparlisib-treated cells relative to that the in indicated DMSO-treated TNBC cells (n = 3 biological replicates/group). *ACTINB* was used for normalization.

(legend continued on next page)

factors also significantly correlated (either negatively or positively) with the expression of PDE7A mRNA in breast cancer samples. We found that 7 out of 10 transcription factors displayed significant correlation with the mRNA expression of PDE7A in breast cancer samples (Figures 2E and S6). We thus prioritized these 7 transcription factors for further analysis. As a first step, we asked which out of these 7 transcription factors, similar to PDE7A, were also regulated by the PI3K-AKT pathway. To test this, we treated TNBC cell lines (MDA-MB-231 and MDA-MB-468) with buparlisib and analyzed the expression of these 7 transcription factors. We found that the expression of only IRF1 was consistently downregulated following buparlisib treatment in all tested TNBC cell lines (Figures 2F, 2G, and S7). To directly test the involvement of IRF1 in stimulating PDE7A expression in TNBC cells, we knocked down IRF1 expression using short hairpin RNAs (shRNAs) in MDA-MB-231 cells and tested its impact on the expression of PDE7A mRNA and protein. *IRF1* knockdown resulted in reduced expression of PDE7A at both mRNA and protein levels (Figures 2H and 2I). We then performed a cleavage under targets & release using nuclease (CUT-&-RUN) assay to monitor the recruitment of IRF1 on the PDE7A promoter. This assay showed the recruitment of IRF1 on the PDE7A promoter (Figure 2J), which indicates that PDE7A is a direct transcriptional target of IRF1. These results collectively demonstrate that PDE7A expression is stimulated via the action of the PI3K-AKT-IRF1 pathway in TNBC cells (Figure 2K).

PDE7A inhibition blocks growth and metastatic attributes in cell culture models of TNBC

We next asked if PDE7A plays a role in driving TNBC cell growth and metastatic attributes in cell culture models of TNBC. We first tested the impact of pharmacological inhibition of PDE7A on TNBC tumor and metastatic characteristics using cell culture-based assays. For this purpose, we used the PDE7A inhibitor BRL-50481. BRL-50481 is a selective, substrate-competitive PDE7 inhibitor with an half-maximal inhibitory concentration (IC_{50}) of 0.15 μM for PDE7A and with more than 200-fold higher selectivity for PDE7 family members than for other PDE family members.[40] Previous studies have demonstrated that PDE7A regulates cAMP response element-binding protein (CREB) activity by modulating intracellular cAMP levels, which subsequently influences protein kinase A (PKA) activity and, in turn, the phosphorylation of CREB at serine 133, ultimately controlling CREB-dependent signaling pathways (Figure S8A).[41] As expected, treatment of TNBC cell lines (MDA-MB-231, MDA-MB-468, and BT-549) with BRL-50481 resulted in increased intracellular cAMP levels (Figure S8B) and, thus, increased CREB phosphorylation (Figure S8C). These TNBC cell lines also showed higher expression of PDE7A compared to the hTERT-HME1 (Figure S8D). Furthermore, consistent with the higher level of PDE7A in TNBC cells compared to other non-TNBC cell lines, TNBC cells showed significantly lower levels of cAMP (Figure S8E).

We next tested the effect of BRL-50481 treatment on the short-term survival of TNBC cells using a methylthiazole tetrazolium (MTT)-based cell viability assay. The results of the MTT assay showed that treatment with BRL-50481 significantly inhibited the viability of TNBC cell lines (MDA-MB-231, BT-549, and MDA-MB-468) (Figure S9A). We then used a long-term cell culture-based clonogenic assay to monitor the impact of BRL-50481 treatment on the long-term colony-forming ability of TNBC cells. We found that the treatment of TNBC cell lines with BRL-50481 suppressed their colony-forming ability (Figure S9B). Additionally, using both qualitative and quantitative soft-agar assays, we monitored the effect of BRL-50481 treatment on the anchorage-independent growth of TNBC cell lines. The soft-agar assay is a surrogate assay for measuring the tumor-forming potential of cancer cells.[42] We found that BRL-50481 treatment significantly inhibited the growth of TNBC cell lines in both the qualitative (Figure 3A) and quantitative (Figure 3B) soft-agar assays.

We then examined whether BRL-50481 treatment affected the metastatic attributes of TNBC cells. Following the treatment of TNBC cell lines with BRL-50481, we measured the invasiveness and migratory capacity using Matrigel-based invasion and wound-healing assays, respectively. BRL-50481 treatment significantly inhibited both the invasive (Figures 3C and 3D) and migratory capabilities of TNBC cells (Figures S9C and S9D). However, unlike TNBC cells, BRL-50481 treatment did not potently inhibit the growth of non-TNBC cells (Figure S10A) or suppress the invasion ability of these cells (Figures S10B and S10C). Similarly, no significant tumor suppression of non-TNBC cells was observed in mice following BRL-50481 treatment (Figure S10D).

To further strengthen these findings, we genetically knocked down the expression of *PDE7A* using shRNA and measured the impact on tumor cell growth and metastatic attributes. Genetic knockdown of *PDE7A* (Figure S11A) also inhibited TNBC growth in a quantitative soft-agar assay (Figure S11B) and suppressed invasion in a Matrigel-based invasion assay (Figures S11C and S11D). Collectively, these results demonstrate that PDE7A inhibition suppresses TNBC growth and metastatic attributes in cell culture models of TNBC.

PDE7A inhibition suppresses tumor growth in mouse models of TNBC

Next, we assessed whether PDE7A inhibition could suppress tumor growth *in vivo* using complementary mouse models of TNBC

(G) IRF1 protein expression was measured using immunoblot analysis under the indicated conditions. ACTINB was used as a loading control.
(H) MDA-MB-231 cells expressing either non-specific (NS) shRNAs or *IRF1* shRNAs were analyzed for *IRF1* and *PDE7A* mRNA expression using RT-qPCR. *IRF1* and *PDE7A* mRNA expression relative to NS shRNA-expressing cells is plotted ($n = 3$ biological replicates/group). *ACTINB* was used for normalization.
(I) MDA-MB-231 cells expressing non-specific shRNA or shRNAs targeting *IRF1* were analyzed for the indicated proteins by immunoblotting. ACTINB was used as a loading control.
(J) (CUT-&-RUN analysis of IRF1 binding on the *PDE7A* promoter. Immunoglobulin G (IgG) was used as a negative control for CUT-&-RUN, and fold enrichment plotted relative to IgG is shown ($n = 3$ biological replicates/group).
(K) A schematic showing the mechanism of PDE7A transcription upregulation by the PI3K-AKT-IRF1 pathway in TNBC.
All quantitative data represent the mean ± SEM; $^{**}p < 0.01$, $^{***}p < 0.001$, and $^{****}p < 0.0001$. See also Figures S4–S7.

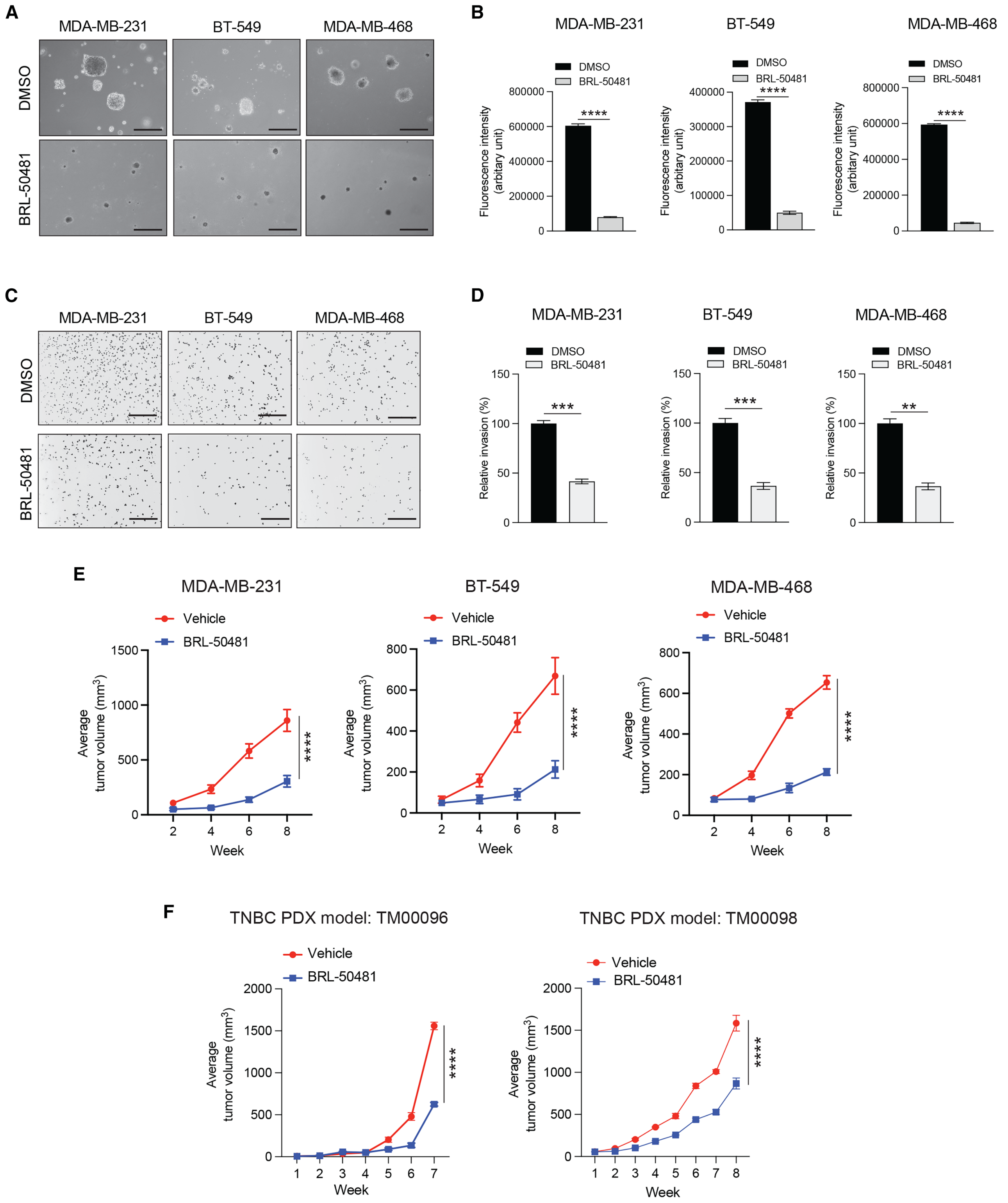

Figure 3. Pharmacological PDE7A inhibition suppresses TNBC in cell culture and in mice

(A) The indicated TNBC cell lines were treated with BRL-50481 (50 μM) or DMSO, and soft-agar assays were performed. Representative images of the soft-agar assays under DMSO or BRL-50481 (50 μM) treatment conditions are shown. Scale bar, 500 μm.

(legend continued on next page)

tumor growth. We first employed a subcutaneous xenograft mouse model of TNBC tumor growth with MDA-MB-231 cells. The mice were treated with either vehicle or BRL-50481, and tumor growth was measured every week. Compared to vehicle-treated mice, BRL-50481 treatment significantly suppressed MDA-MB-231 xenograft tumor growth (Figure 3E). Similar tumor suppression by BRL-50481 was also observed in other TNBC tumor cell line xenograft models derived from MDA-MB-468 and BT-549 cells (Figure 3E).

To further bolster the impact of our findings and increase the clinical relevance of therapeutically targeting PDE7A in TNBC, we tested the efficacy of BRL-50481 treatment in two patient-derived xenograft (PDX) models of TNBC. Both TNBC PDX models (TM00096 and TM00098) expressed higher levels of PDE7A compared to hTERT-HME1 (Figure S8F). PDX cancer models closely recapitulate several key tumor characteristics observed in human patients, including intratumoral heterogeneity, and thus are considered superior to established cell line-based models.[43,44] TNBC PDXs were implanted into the flanks of female NOD *scid* gamma **NOD *(* gamma (**NSG) mice, and the mice were treated with either vehicle or BRL-50481. We found that treatment with BRL-50481 significantly inhibited tumor growth of both TNBC PDXs in mice when compared to the vehicle treatment group (Figure 3F). Collectively, these results demonstrate that PDE7A inhibition by BRL-50481 potently suppresses TNBC tumor growth in both TNBC cell line xenograft- and PDX xenograft-based mouse models.

PDE7A inhibition attenuates *de novo* pyrimidine biosynthesis in TNBC

We next performed experiments to determine the mechanism underlying the PDE7A inhibition-mediated suppression of TNBC cell growth. We first performed RNA sequencing (RNA-seq) to identify changes in the transcriptional profiles of TNBC cells following treatment of MDA-MB-231 cells with BRL-50481 and used DMSO-treated MDA-MB-231 cells as controls. RNA-seq analysis identified several differentially expressed genes (Figure 4A; Table S2), including 4,084 significantly upregulated and 4,365 significantly downregulated genes in BRL-50481-treated condition compared with DMSO-treated MDA-MB-231 cells ($p < 0.05$; Table S2). Biological pathway analysis revealed that BRL-50481 treatment of MDA-MB-231 cells resulted in the downregulation of multiple genes involved in cellular biosynthesis pathways (Figures 4B and S12; Table S3).

Because our RNA-seq analysis identified that PDE7A inhibition downregulated multiple genes involved in cellular biosynthetic pathways in TNBC cells, therefore, we performed a large-scale, unbiased metabolomics analysis of MDA-MB-231 cells treated with either BRL-50481 or DMSO as a control to identify metabolites that are downregulated concomitantly with the transcriptional repression of metabolic genes. Metabolomic analysis was performed using capillary electrophoresis time-of-flight mass spectrometry in two modes for cationic and anionic metabolites. We detected 287 metabolites (151 metabolites in cation mode and 136 metabolites in anion mode) (Figures 4C and S13; Table S4). We then analyzed both the RNA-seq and metabolomics data to identify changes that can be integrated at both the mRNA and metabolite levels. This analysis identified the downregulation of genes encoding pyrimidine biosynthesis enzymes, such as carbamoyl-phosphate synthetase 2, aspartate transcarbamylase, and dihydroorotase (CAD) and DHODH (Figure 4D) in MDA-MB-231 cells treated with BRL-50481 compared with cells treated with DMSO, and a trend of downregulation of metabolites of *de novo* pyrimidine biosynthesis pathway, including dihydroorotic acid, uridine monophosphate (UMP), and deoxythymidine monophosphate (dTMP) (Figure 4E).

The *de novo* pyrimidine biosynthesis pathway is essential for synthesizing the nucleotides such as uridine (precursor to uracil), cytidine (precursor to cytosine), and thymidine (precursor to thymine), which are key components of RNA and DNA. It is crucial for maintaining the balance of nucleotide pools necessary for DNA replication and RNA transcription, thereby supporting cellular growth and division.[45,46] In certain cancer types and genetic contexts, cancer cells rely on the de novo pyrimidine biosynthesis pathway for survival,[47–49] positioning it as a promising therapeutic target with potential clinical value. We further validated our RNA-seq results and confirmed reduced expression of *de novo* pyrimidine biosynthesis pathway genes *DHODH* and *CAD* in multiple TNBC cell lines treated with BRL-50481 compared with cells treated with DMSO (Figures 4F–4H). Furthermore, consistent with the BRL-50481, genetic knockdown of PDE7A using shRNAs also resulted in the downregulation of genes encoding pyrimidine biosynthesis enzymes, such as DHODH and CAD (Figure S14A). Collectively, these results demonstrate that inhibition of PDE7A attenuates *de novo* pyrimidine biosynthesis pathway by repressing the expression of DHODH and other *de novo* pyrimidine biosynthesis enzymes.

(B) The indicated TNBC cell lines were treated with DMSO or BRL-50481 (50 μM), and the quantitative soft-agar assay was performed using the CytoSelect 96-well quantitative soft-agar assay kit. Fluorescence intensities (arbitrary unit) under the indicated conditions for the indicated TNBC cell lines are shown (n = 3 biological replicates/group).

(C) The indicated TNBC cell lines were treated with DMSO or BRL-50481 (50 μM) for 20 h and analyzed for invasive capacity using a Matrigel-based invasion assay. Representative images are shown. Scale bar, 200 μm.

(D) Relative invasion (%) in the BRL-50481-treated condition as compared to the DMSO-treated condition is plotted for the experiment shown in (C) (n = 3 biological replicates/group).

(E) The indicated TNBC cell lines cells were injected subcutaneously into the flanks of female NSG mice (n = 5/group, each cell line). Mice were treated every alternate day with vehicle or BRL-50481 (25 mg/kg) intraperitoneally, and tumor growth was measured. Average tumor volumes at the indicated time points are plotted.

(F) TNBC PDX models TM00098 and TM00098 were subcutaneously injected into the flanks of female NSG mice (n = 6/group, each PDX). The mice were treated every alternate day with vehicle or BRL-50481 (25 mg/kg) intraperitoneally, and tumor growth was measured. Average tumor volume at the indicated time points is plotted.

All quantitative data represent the mean ± SEM. **$p < 0.01$, ***$p < 0.001$, and ****$p < 0.0001$. See also Figures S8–S11.

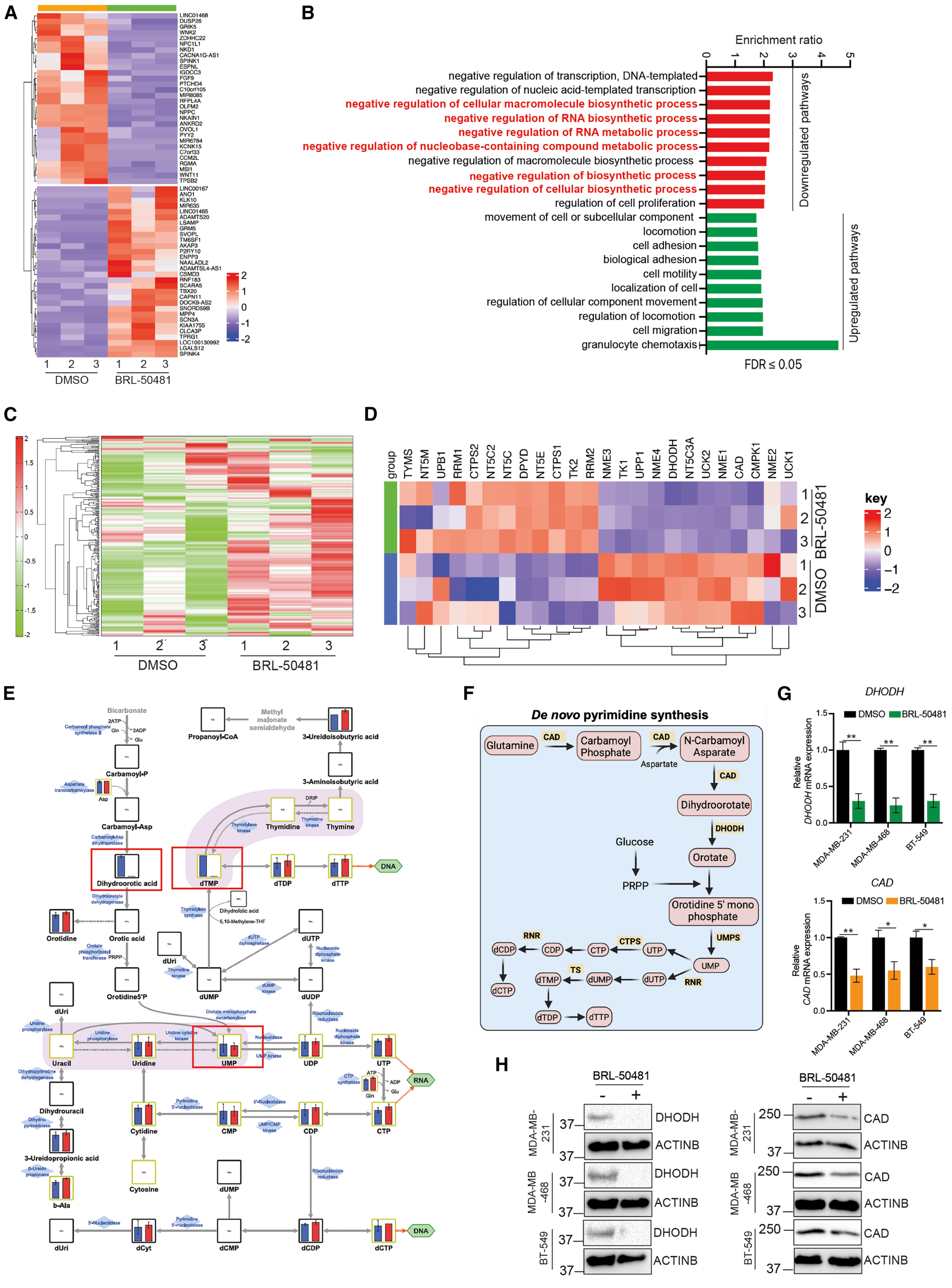

(legend on next page)

DHODH mediates the tumor-promoting effects of PDE7A in TNBC

The *de novo* pyrimidine biosynthesis pathway is regulated by several enzymes, such as DHODH, that can be pharmacologically targeted, presenting therapeutic opportunities in cancers with dependency on this pathway.[50] We performed further studies on DHODH because DHODH inhibitors have shown clinical efficacy for the treatment of rheumatoid and psoriatic arthritis.[51] Additionally, several DHODH inhibitors have entered clinical testing phases against various cancer types (ClinicalTrial.gov ID# NCT03451084; NCT01992367, and NCT03404726).[52–54] We first asked whether PDE7A can promote DHODH expression. Therefore, we ectopically expressed PDE7A in hTERT-HME1 cells. We found that ectopic expression of PDE7A promoted the expression of DHODH (Figure S14B). These results are consistent with the role of PDE7A in promoting the expression of DHODH in TNBC. Similarly, DHODH was downregulated in TNBC cells upon treatment with buparlisib (Figures S14C and S14D), while DHODH was upregulated following ectopic expression of constitutively active PIK3CA in hTERT-HME1 cells (Figure S14E). Collectively, these results show that PDE7A stimulates the expression of DHODH in TNBC cells.

We then asked how PDE7A promotes the expression of DHODH in TNBC cells. Since the effect was at the mRNA level, we focused on the transcription-based regulation of DHODH in TNBC cells. To identify potential regulators of DHODH downstream of PDE7A, we re-analyzed our RNA-seq data from BRL-50481-treated TNBC cells. We found that, similar to DHODH, transcription factor E2F1 was also downregulated following BRL-50481 treatment in the RNA-seq data (Table S2). We also identified E2F1-binding sites on DHODH promoter DNA (Figure S15A). Based on these findings, we performed experiments to determine the role of E2F1 transcription factor downstream of PDE7A in regulating the expression of DHODH. We first measured the *E2F1* mRNA and protein levels in TNBC cell lines (MDA-MB-231 and MDA-MB-468) treated with BRL-50481. BRL-50481 treatment resulted in decreased expression of *E2F1* mRNA (Figure S15B) and protein (Figure S15C) levels. Furthermore, consistent with the role of E2F1 in directly regulating the transcription of DHODH, in a CUT-&-RUN assay, we found that E2F1 was enriched on the DHODH promoter, and this enrichment was inhibited after BRL-50481 treatment (Figure S15D). We then directly tested the impact of knocking down *E2F1* on DHODH expression in TNBC cells. shRNA-mediated knockdown of *E2F1* resulted in reduced DHODH mRNA (Figure S15E) and protein levels (Figure S15F). Collectively, these results demonstrate that E2F1 downstream of PDE7A stimulates the expression of DHODH in TNBC cells.

Next, we examined whether DHODH inhibition exerts similar tumor-suppressive effects on TNBC cells as those observed in response to PDE7A inhibition. We used a DHODH inhibitor BAY-2402234, which has an IC_{50} of 1.2 nM.[55] BAY-2402234 and other DHODH inhibitors are currently being evaluated in clinical trials, with a focus on its ability to trigger cell differentiation and halt tumor growth, particularly myeloid malignancies.[56] We treated TNBC cell lines (MDA-MB-231, MDA-MB-468, and BT-549) with increasing concentrations of BAY-2402234 and measured cell proliferation using an MTT-based cell viability assay, which showed that BAY-2402234 effectively inhibited TNBC cell viability (Figure 5A). We then performed qualitative and quantitative soft-agar assays. In both qualitative and quantitative soft-agar assays, treatment of TNBC cell lines (MDA-MB-231, MDA-MB-468, and BT-549) with BAY-2402234 inhibited their ability to form colonies (Figures 5B and 5C). However, unlike TNBC cell lines, non-TNBC cell lines showed variable response to BAY-2402234, and growth suppression was not as effective as observed in the case of TNBC cell lines (Figure S16).

We next asked if, similar to cell culture-based assays, BAY-2402234 can suppress tumor growth *in vivo*. To test this, TNBC cell lines (MDA-MB-231, MDA-MB-468, and BT-549) were injected subcutaneously into female NSG mice and were treated with either vehicle or BAY-2402234. We observed that, in a mouse model of TNBC xenograft, treatment with BAY-2402234 also effectively blocked TNBC tumor growth (Figure 5D).

Next, to establish that the effect of DHODH inhibition by BAY-2402234 on TNBC growth was driven by reduced pyrimidine biosynthesis, we performed a pyrimidine biosynthesis rescue experiment by uridine supplementation. Uridine supplementation

Figure 4. Pharmacological PDE7A inhibition suppresses multiple genes that encode enzymes regulating the pyrimidine biosynthesis pathway in TNBC

(A) Heatmap showing the top 30 upregulated and top 30 downregulated genes in MDA-MB-231 cells treated with BRL-50481 (50 μM) for 72 h compared with DMSO-treated cells (n = 3 biological replicates/group).

(B) Biological pathways that were altered in MDA-MB-231 cells treated with the PDE7A inhibitor BRL-50481 (50 μM) for 72 h compared with DMSO-treated cells based on the mRNA expression profiles identified from RNA sequencing results.

(C) Heatmap showing metabolite levels of altered metabolites in MDA-MB-231 cells treated with BRL-50481 (50 μM) for 72 h compared with DMSO-treated cells (n = 3 biological replicates/group).

(D) Heatmap showing mRNA levels of altered pyrimidine biosynthesis genes in MDA-MB-231 cells treated with BRL-50481 (50 μM) for 72 h compared with DMSO-treated cells (n = 3 biological replicates/group).

(E) Schematic for the pyrimidine biosynthesis pathway showing metabolite levels measured through our global metabolomics analysis. Dihydroorotic acid, UMP, and dTMP levels are highlighted in red rectangles.

(F) A schematic showing metabolites and enzymes of the *de novo* pyrimidine biosynthesis pathway.

(G) *DHODH* and *CAD* mRNA expression levels in the indicated TNBC cells treated with 50 μM BRL-50481 for 72 h compared with DMSO-treated cells are shown. *ACTINB* was used for normalization (n = 3 biological replicates/group).

(H) DHODH and CAD protein levels in MDA-MB-231 cells treated with 50 μM BRL-50481 or DMSO for 72 h were measured using immunoblotting. ACTINB was used as a loading control.

All quantitative data represent the mean ± SEM. *p < 0.05 and **p < 0.01. See also Figures S12–S14 and Tables S2, S3, and S4.

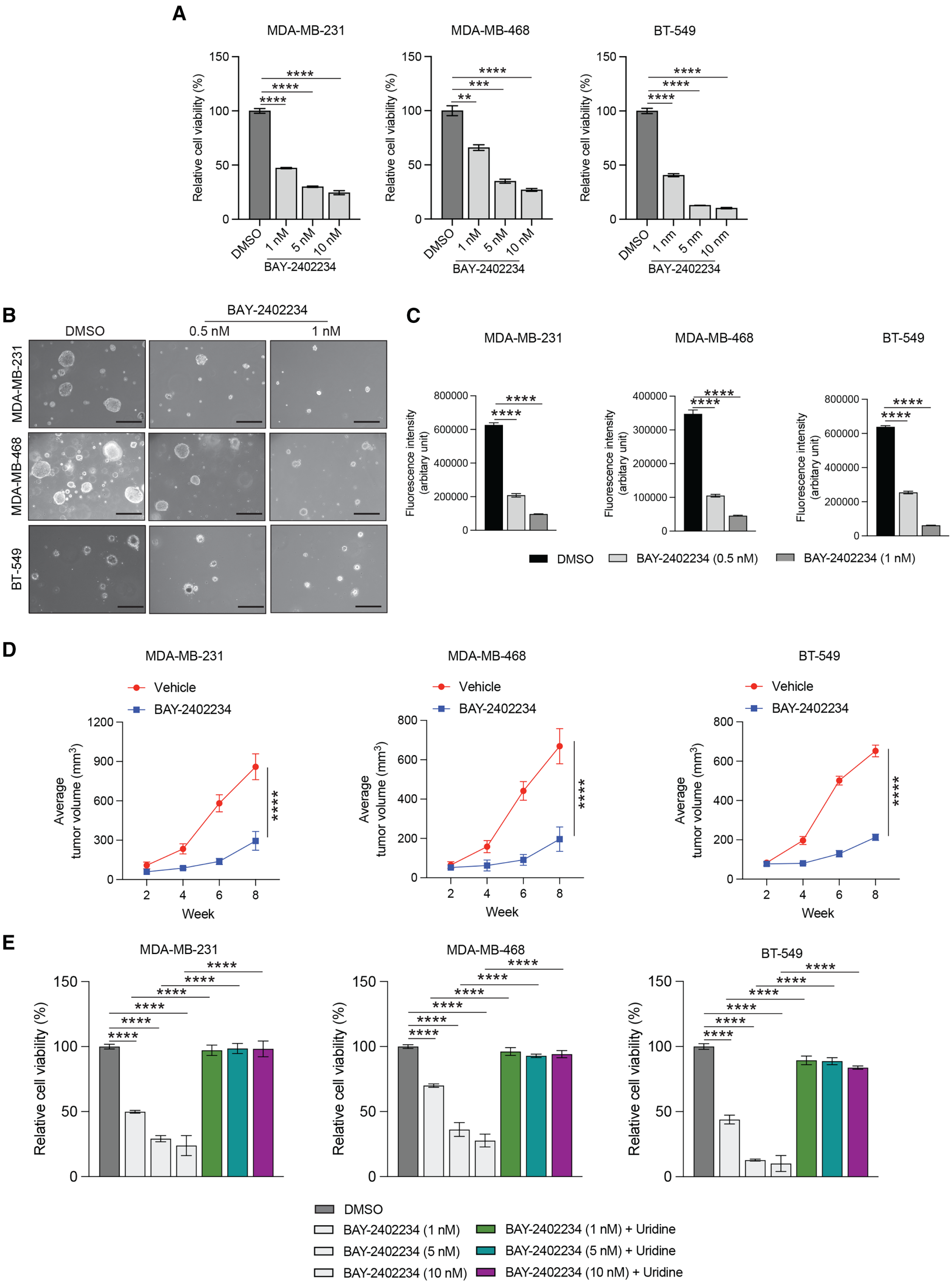

Figure 5. Pharmacological inhibition of DHODH suppresses TNBC tumor growth

(A) The indicated TNBC cell lines were treated with BAY-2402234 at the indicated concentrations for 72 h and analyzed for cell viability using the MTT assay. Relative cell viability is plotted relative to DMSO-treated cells (*n* = 3 biological replicates/group).

(B) TNBC cell lines were treated with BAY-2402234 at the indicated concentrations, and soft-agar assays were performed. Representative images of soft-agar assays for the indicated TNBC cell lines treated with BAY-2402234 (0.5 or 1 nM) are shown. Scale bar, 500 μm.

(legend continued on next page)

rescues the effects of *de novo* pyrimidine synthesis inhibition by bypassing the need for intracellular pyrimidine production, providing an exogenous source of pyrimidines. Consistent with the role of pyrimidine biosynthesis as a mediator of the effect of DHODH inhibition, uridine supplementation rescued the growth of TNBC cells treated with BAY-2402234 (Figure 5E).

We then asked whether DHODH is a downstream mediator of PDE7A function in TNBC. First, we generated a PDE7A knockout (PDE7A-KO) cell line using a CRISPR-based single guide RNA (sgRNA) in MDA-MB-231 cells (Figure 6A). PDE7A-KO, similar to the pharmacological inhibition of PDE7A, resulted in significant TNBC growth inhibition in both qualitative and quantitative soft-agar assays (Figures 6B and 6C). We next overexpressed DHODH in PDE7A-KO cells (Figure 6D) and examined whether DHODH ectopic expression could rescue the growth of PDE7A-KO TNBC cells. DHODH ectopic expression partially but significantly rescued the growth of PDE7A-KO MDA-MB-231 cells in both qualitative and quantitative soft-agar assays (Figures 6E and 6F). This rescue in growth was due to reduced apoptosis following DHODH ectopic expression in PDE7A knockout TNBC cells, as observed by reduced caspase-3 activity (Figure 6G). We next established the role of DHODH as a downstream mediator of PDE7A using a mouse xenograft model. PDE7A-KO TNBC cells without or with DHODH overexpression were subcutaneously injected into the flanks of female NSG mice, and tumor growth was measured. In a mouse xenograft model also, ectopic expression of DHODH in PDE7A-KO TNBC cells rescued their tumor growth (Figure 6H).

Next, we tested whether the enzymatic activity of DHODH is required for its ability to promote TNBC growth downstream of PDE7A. Therefore, we ectopically expressed wild-type DHODH or a DHODH mutant, DHODH (R135C), that is catalytically inactive and, thus, lacks oxidoreductase activity in PDE7A-KO TNBC cells (Figure S17A) and examined whether DHODH ectopic expression could rescue the growth of PDE7A-KO TNBC cells. Expression of wild-type DHODH partially but significantly rescued the growth of PDE7A-KO MDA-MB-231 cells in the quantitative soft-agar assay (Figure S17B), while catalytically inactive DHODH mutant DHODH (R135C) failed to do so (Figure S17B). Similarly, PDE7A-KO inhibited the invasion ability of TNBC cells, which was rescued by the ectopic expression of wild-type DHODH but not by DHODH (R135C) (Figures S17C and S17D). This rescue in growth following DHODH ectopic expression in PDE7A-KO TNBC cells was due to reduced apoptosis, as observed by reduced caspase-3 activity in wild-type DHODH-expressing PDE7A-KO TNBC cells but not in cells expressing DHODH (R135C) (Figure S17E). Consistent with this, BRL-50481-treated cells expressing wild-type DHODH showed restored level of downstream metabolites of the *de novo* pyrimidine biosynthesis pathway, but mutant DHODH (R135C) failed to do so (Figure S17F).

Combinatorial targeting of PDE7A and DHODH potently suppresses TNBC tumor growth and metastasis

As both BRL-50481 and BAY-2402234 inhibited TNBC in both cell culture and mouse models of TNBC, we tested whether combining BRL-50481 and BAY-2402234 could achieve even stronger suppression of TNBC tumor growth and metastasis. The rationale for combining both PDE7A and DHODH inhibitors is supported by previous studies in which targeting multiple nodes of the same cancer driver pathway has been shown to achieve better clinical outcomes.[57–60] We first tested the effects of these inhibitors, either alone or in combination, on TNBC growth using qualitative and quantitative soft-agar assays and found that combining BRL-50481 with BAY-2402234 resulted in a more potent growth inhibition than either inhibitor alone (Figures 6I and 6J). Combined treatment of TNBC cells with BRL-50481 and BAY-2402234 was more effective in inducing apoptosis than either inhibitor alone, offering a potential mechanistic explanation for the enhanced therapeutic effect of combination therapy (Figure 6K). Similarly, we tested the combination of 5-fluorouracil (5-FU) and BRL-50481 based on the previous studies in which DHODH inhibitor and 5-FU have shown synergistic effects in tumor suppression[61–63] and the fact that PDE7A inhibition can suppress DHODH expression. As expected, 5-FU and BRL-50481 combination was more effective in inhibiting tumor growth compared to either of these drugs alone in inhibiting TNBC cell growth and metastatic attributes in MTT assay (Figure S18A), quantitative soft-agar assay (Figure S18B), and Matrigel-based invasion assay (Figures S18C and S18D).

We extended our studies with this combination in TNBC PDX mouse models. TNBC PDXs (TM00096 and TM00098) were injected subcutaneously into the flanks of female NSG mice and were treated with BRL-50481 and BAY-2402234 either alone or in combination. Combined BRL-50481 and BAY-2402234 treatment caused more potent TNBC tumor growth inhibition than treatment with either inhibitor alone or vehicle (Figures 7A and 7B).

We next tested the effects of combined BRL-50481 and BAY-2402234 treatment in an orthotopic xenograft mouse model of TNBC tumor growth and spontaneous metastasis, which recapitulates several aspects of breast cancer observed in humans, including a multistage metastatic process that mirrors metastatic progression as it occurs in breast cancer patients.[64] We orthotopically engrafted MDA-MB-231-*F-Luc* cells into the mammary fat pad of female NSG mice and treated mice with vehicle, BRL-50481 alone, BAY-2402234 alone, or a combination of

(C) The indicated TNBC cell lines were treated with DMSO or BAY-2402234 (0.5 or 1 nM), and a quantitative soft-agar assay was performed using the CytoSelect 96-well quantitative soft-agar assay kit. Fluorescence intensities (arbitrary unit) under the indicated conditions for the indicated TNBC cell lines are shown (n = 3 biological replicates/group).

(D) The indicated TNBC cell lines were injected subcutaneously into the flanks of female NSG mice (n = 5/group, each cell line). Mice were treated every alternate day with either vehicle or BAY-2402234 (2 mg/kg) intraperitoneally, and tumor growth was measured. Average tumor volumes at the indicated time points are plotted.

(E) MTT assay was performed to measure cell viability for the indicated TNBC cells treated with DMSO or the indicated concentration of BAY-2402234 for 72 h with or without 100 μM uridine (n = 4 biological replicates/group).

All quantitative data represent the mean ± SEM. $^{**}p < 0.01$, $^{***}p < 0.001$, and $^{****}p < 0.0001$. See also Figures S14–S16 and Table S2.

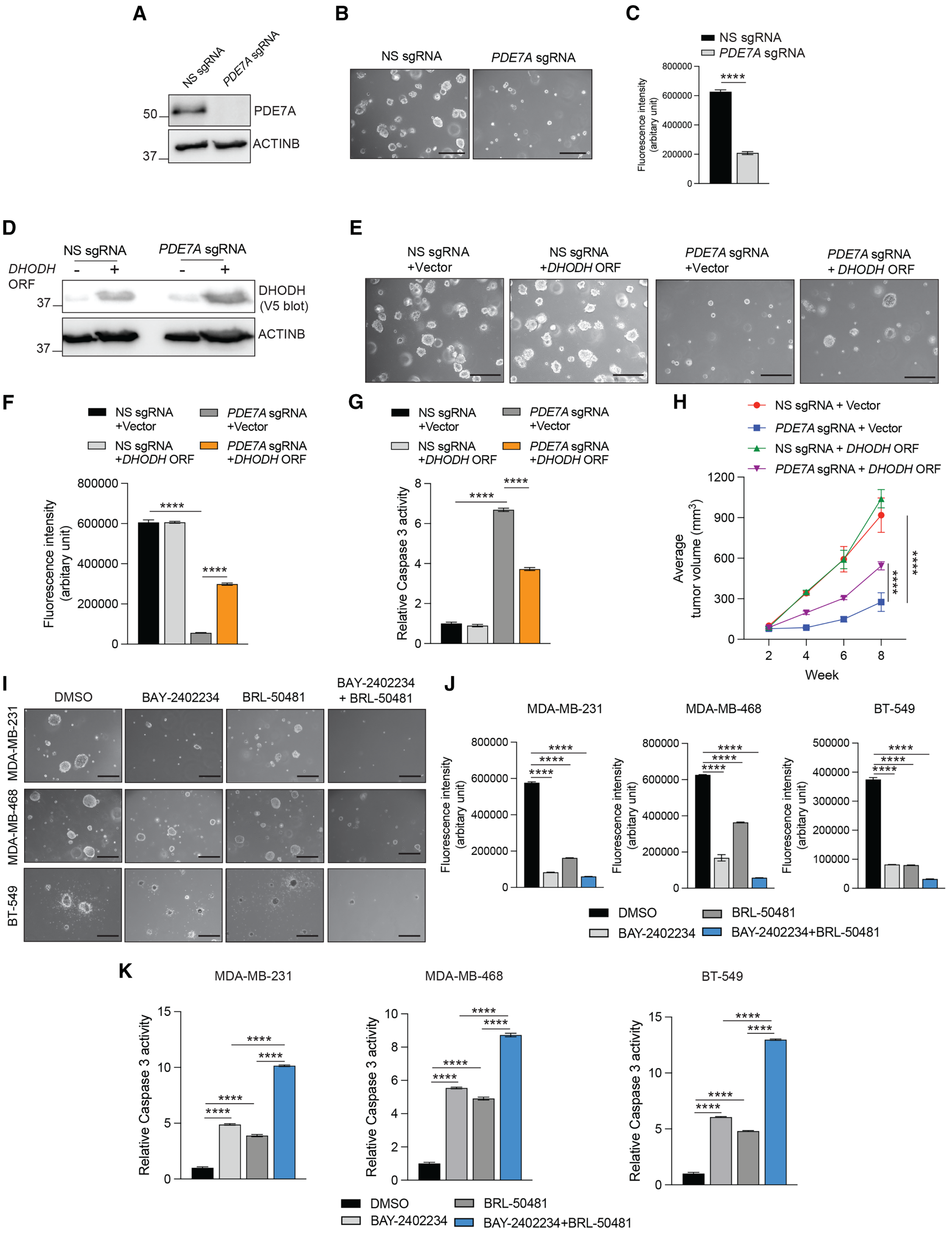

Figure 6. Genetic inhibition of PDE7A suppresses TNBC tumor growth in part via DHODH downregulation, and co-targeting PDE7A and DHODH potently inhibits TNBC

(A) Immunoblotting for PDE7A protein expression in MDA-MB-231 cells expressing non-specific single guide RNA (NS sgRNA) or *PDE7A*-targeting sgRNA. ACTINB was used as a loading control.

(B) MDA-MB-231 cells expressing NS sgRNA or PDE7A-targeting sgRNA were analyzed by soft-agar assay. Representative images of the soft-agar assays for MDA-MB-231 cells expressing either NS sgRNA or *PDE7A*-targeting sgRNA are shown. Scale bar, 500 μm.

(C) MDA-MB-231 cells expressing either NS sgRNA or *PDE7A*-targeting sgRNA were analyzed using the quantitative soft-agar assay using the CytoSelect 96-well quantitative soft-agar assay kit. Fluorescence intensities (arbitrary unit) under the indicated conditions are shown (n = 3 biological replicates/group).

(legend continued on next page)

BRL-50481 and BAY-2402234. Tumor growth and metastasis to distal organs were assessed by monitoring *F-Luc* activity via bioluminescence imaging using the Xenogen *in vivo* imaging system. The combination of BRL-50481 and BAY-2402234 treatment significantly inhibited orthotopic tumor growth compared with either single-drug treatments (Figures 7C and 7D). We also observed significant reductions in spontaneous metastasis to the lungs and liver in all treatment groups compared with the vehicle-treated control group (Figures 7E and 7F).

Collectively, these *in vivo* studies demonstrate that combined BRL-50481 and BAY-2402234 treatment results in potent inhibition of tumor growth and metastasis in clinically relevant orthotopic and PDX mouse models of TNBC. These results provide evidence to support the superior therapeutic benefits offered by combining BRL-50481 with BAY-2402234 against TNBC tumor growth and metastasis.

DISCUSSION

In this study, we identified the PDE7A→DHODH→*de novo* pyrimidine biosynthesis pathway as an important driver of TNBC and as a potential therapeutic target of clinical value for TNBC treatment (Figure 7G). Cyclic nucleotide PDEs hydrolyze cAMP or cyclic guanosine monophosphate (cGMP), playing central roles in cyclic nucleotide signaling regulation. In humans, 21 different genes encode PDEs, and additional diversity is derived from alternative mRNA splicing, the use of alternative promoters, and other mechanisms,[23–25] resulting in more than 100 different PDE isoforms with distinct tissue-specific expression patterns and functions.[23–25] PDE7A belongs to the PDE7 family and functions as a cAMP-specific PDE.[26,27] Previous studies have shown that PDE proteins play important roles in cancer.[28–30] One study showed that PDE10A suppresses β-catenin and Rat sarcoma (RAS) signaling in ovarian cancer.[65] Similarly, PDE3A has been shown to drive stem cell-like properties and metastasis in breast cancer.[66] Another study implicated PDE4D in the suppression of the AKT-mechanistic target of rapamycin (mTOR)-MYC proto-oncogene, BHLH transcription factor (MYC) signaling pathway, reducing the malignant properties of colon cancer cells.[67] However, the role of PDE7A in TNBC has not been explored previously. We found that PDE7A is overexpressed in TNBC by the action of PI3K/AKT pathway via the transcription factor IRF1. Furthermore, PDE7A inhibition blocked TNBC tumor growth and metastasis, which underpins its role as an important driver of TNBC.

Pyrimidine biosynthesis occurs via two main pathways: *de novo* and salvage.[68] In cancer cells, the *de novo* pathway is more frequently upregulated, allowing them to bypass the limited availability of pyrimidine bases required for the salvage pathway in fast-growing tumor environments.[69] mTORC1, RAS-Mitogen-activated protein kinase kinase (MEK)-extracellular signal-regulated kinase (ERK), and PI3K-AKT pathways have all been previously implicated in driving deregulation of pyrimidine biosynthesis pathway.[50,70,71] We have identified a previously undocumented mechanism through which PDE7A loss results in the attenuation of the *de novo* pyrimidine biosynthesis pathway by modulating the expression of *de novo* pyrimidine biosynthesis pathway enzymes, including DHODH. Furthermore, ectopic expression of the pyrimidine biosynthesis enzyme DHODH was able to rescue TNBC growth following the loss of PDE7A, establishing DHODH and the pyrimidine biosynthesis pathway as important downstream mediators of TNBC-driving function of PDE7A. These results identified a previously undocumented role for PDE7A in the regulation of DHODH and stimulation of the *de novo* pyrimidine biosynthesis pathway.

PDE family proteins and pyrimidine biosynthesis pathway enzymes have been previously explored as potential therapeutic targets in some cancers.[50,72] DHODH inhibitors are in clinical trials to treat a series of different cancer types.[54] In particular, in some cancers with IDH1/IDH2 mutations, such as gliomas, cells become reliant on the *de novo* pyrimidine synthesis pathway.[47,48] DHODH inhibition in this context can cause nucleotide pool

(D) MDA-MB-231 cells expressing an empty vector or V5-tagged *DHODH* open reading frame (ORF)-expressing cells simultaneously expressing NS sgRNA or *PDE7A*-targeting sgRNA. *DHODH* protein expression was assessed by an antibody for V5-tag. ACTINB was used as the loading control.

(E) MDA-MB-231 cells expressing an empty vector or *DHODH* ORF-expressing cells simultaneously expressing NS sgRNA or *PDE7A*-targeting sgRNA were analyzed using a soft-agar assay. Representative images of the soft-agar assay under indicated conditions are shown. Scale bar, 500 μm.

(F) MDA-MB-231 cells expressing an empty vector or V5-tagged DHODH ORF-expressing cells simultaneously expressing NS sgRNA or PDE7A-targeting sgRNA were analyzed using the quantitative soft-agar assay performed with the CytoSelect 96-well quantitative soft-agar assay kit. Fluorescence intensities (arbitrary unit) under the indicated conditions are shown (n = 3 biological replicates/group).

(G) MDA-MB-231 cells expressing NS sgRNA or *PDE7A*-targeting sgRNA and simultaneously expressing *DHODH* ORF or an empty vector control were analyzed for caspase-3 activity using the caspase-3-based colorimetric assay. Relative caspase-3 activity under the indicated conditions is shown (n = 4 biological replicates/group).

(H) MDA-MB-231 cells expressing NS sgRNA or *PDE7A*-targeting sgRNA and simultaneously expressing *DHODH* ORF or an empty vector control were subcutaneously injected into the flanks of female NSG mice (n = 5/group), and tumor volumes were measured. Average tumor volumes at the indicated times are shown.

(I) The indicated TNBC cell lines were treated with DMSO, BAY-2402234 (0.5 nM), BRL-50481 (20 μM), or both BAY-2402234 (0.5 nM) and BRL-50481 (20 μM) and were analyzed by the soft-agar assay. Representative images of the soft-agar assays for these indicated TNBC cell lines under the indicated conditions are plotted. Scale bar, 500 μm.

(J) The indicated TNBC cell lines were treated with DMSO, BAY-2402234 (0.5 nM), BRL-50481 (20 μM), or both BAY-2402234 (0.5 nM) and BRL-50481 (20 μM) and were analyzed using the quantitative soft-agar assay performed using the CytoSelect 96-well quantitative soft-agar assay kit. Fluorescence intensities (arbitrary unit) under the indicated conditions are shown (n = 3 biological replicates/group).

(K) The indicated TNBC cell lines were treated with DMSO, BAY-2402234 (0.5 nM), BRL-50481 (20 μM), or BAY-2402234 (0.5 nM) + BRL-50481 (20 μM) and analyzed for caspase-3 activity using a caspase-3-based colorimetric assay. Relative caspase-3 activity is shown under the indicated conditions (n = 4 biological replicates/group).

All quantitative data represent the mean ± SEM. ****$p < 0.0001$. See also Figures S17 and S18.

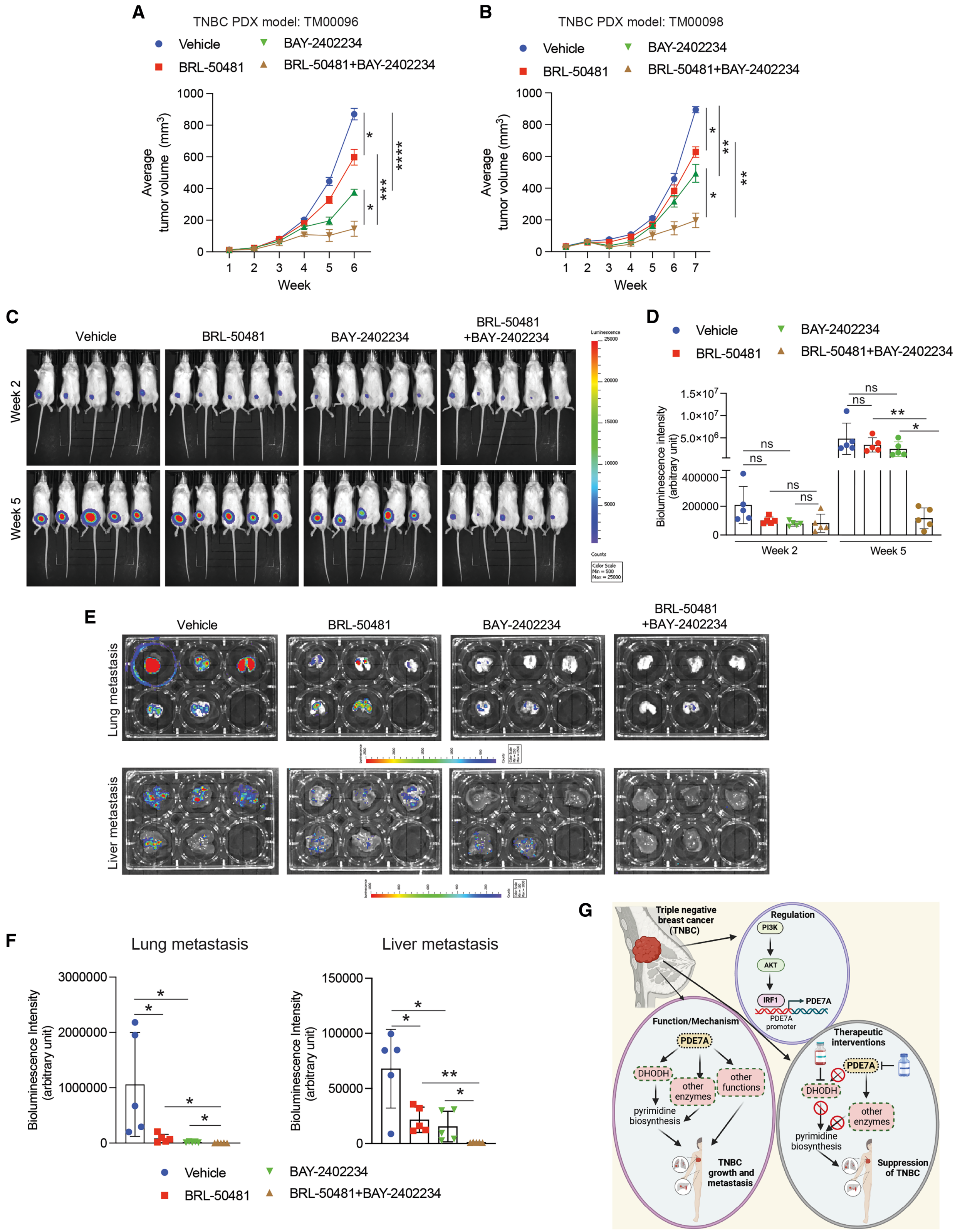

(legend on next page)

imbalance, leading to tumor suppression.[47,48] Additionally, DHODH inhibition has shown to overcome differentiation arrest in HOXA9-expressing acute myeloid leukemia (AML) in both *in vitro* and mouse models of AML.[56] Similarly, in the context of MYC-amplified medulloblastoma, the *de novo* pyrimidine biosynthesis pathway and DHODH emerged as specific vulnerabilities of these cancers, and in these tumors DHODH loss resulted in MYC degradation and pyrimidine deprivation-induced cell-cycle arrest and apoptosis induction.[73] Furthermore, context-dependent function is also identified in case of Kirsten rat sarcoma viral oncogene homolog (KRAS)/LKB1 mutant non-small cell lung cancers where overexpression of CPS1 drove *de novo* pyrimidine biosynthesis pathway dependency.[49]

Our findings that DHODH expression and the *de novo* pyrimidine biosynthesis pathway are stimulated by PDE7A led us to test inhibitors of PDE7A and DHODH in TNBC cells. We found that PDE7A inhibitor, both alone and in combination with a DHODH inhibitor, suppressed TNBC tumor growth and metastasis. Combinatorial treatment with PDE7A and DHODH inhibitors was more effective against TNBC, in part due to their ability to induce more potent apoptosis induction compared to when these drugs are used alone. The combinatorial effects observed with PDE7A and DHODH inhibitors are consistent with what has been observed in the context of mitogen-activated protein kinase signaling cascade; combination therapy targeting B-Raf proto-oncogene, serine/threonine kinase (BRAF) and MEK has shown superior clinical efficacy compared to monotherapy in BRAF-mutant melanomas. Dual inhibition with agents such as dabrafenib (BRAF inhibitor) and trametinib (MEK inhibitor) significantly delays resistance and improves progression-free survival, as BRAF inhibition alone often results in pathway reactivation through MEK or ERK.[74] Another illustrative case is the dual inhibition of epidermal growth factor receptor (EGFR) and ERK in EGFR-mutant non-small cell lung cancer. Combining EGFR and ERK inhibitors has been shown to block this escape route and sustain therapeutic response.[75] Previous studies have shown that inhibition of DHODH and the pyrimidine biosynthesis pathway results in nucleotide pool imbalance, which in turn results in DNA damage and consequentially apoptosis induction.[47–49] This is consistent with our observation of increased apoptosis after DHODH inhibition in TNBC cells. Of note, PDE7A likely influences other cellular pathways besides the *de novo* pyrimidine biosynthesis to drive TNBC growth, which may in part explain both the partial rescue of PDE7 inhibition following DHODH ectopic expression and enhanced TNBC tumor and metastasis suppression by co-targeting of PDE7A and DHODH compared to either inhibitor alone. These findings provide rationale for further evaluating PDE7A inhibitors in the clinic, either alone or in combination with DHODH inhibitors, for TNBC therapy. A favorable outcome of such clinical trials will likely provide new therapeutic options for TNBC patients who currently achieve limited benefits from available therapies.

Limitations of the study

Our study identifies PDE7A and its downstream effector DHODH as important drivers of TNBC. Although various TNBC subtypes have been identified,[76] we did not examine subtype-specific effects of PDE7A. While DHODH was the main focus of our analysis, other metabolic players like CAD may also be relevant. Furthermore, non-metabolic pathways downstream of PDE7A could also contribute to TNBC progression. Future studies are required to further explore these possibilities in greater detail.

RESOURCE AVAILABILITY

Lead contact

Further information and requests for resources and reagents should be directed to and will be fulfilled by the lead contact, Narendra Wajapeyee (nwajapey@uab.edu).

Materials availability

This study did not generate new unique reagents.

Data and code availability

- The RNA-seq data are available at the Gene Expression Omnibus (GEO) under the accession number GEO: GSE192830. The global metabolomic data are available at the National Metabolomics Data Repository (NMDR) under the accession number NMDR: ST004077. All other data are available in the main text or the supplemental information.
- This paper does not report original code.
- Any additional information required to reanalyze the data reported in this paper is available from the lead contact upon request.

ACKNOWLEDGMENTS

We gratefully acknowledge the following grants from the National Institutes of Health: R01CA233481 (R.G.) and R03CA292128 (R.G.). We also acknoweldge the Mary Ann Harvard Grant from O’Neal Comprehensive Cancer Center, UAB to R.G..

AUTHOR CONTRIBUTIONS

R.G. and N.W. designed the experiments. P.M. and S.B. performed the majority of the experiments. R.D. and K.K.R. also performed experiments. K.S.

Figure 7. Pharmacological inhibition of PDE7A and DHODH combinatorically inhibits TNBC tumor growth and metastasis in mice

(A and B) The indicated TNBC PDXs (TM00096 and TM00098) were subcutaneously injected into the flanks of female NSG mice ($n = 6$/group, each PDX). The mice were treated every alternate day with vehicle, BRL-50481 (10 mg/kg), BAY-2402234 (0.5 mg/kg body weight), or the combination of BRL-50481 (10 mg/kg) and BAY-2402234 (0.5 mg/kg) intraperitoneally and analyzed for tumor growth. Average tumor volumes at the indicated time points are shown.

(C) Firefly luciferase-labeled (*F-Luc*) MDA-MB-231 cells were orthotopically injected into the mammary fat pad of female NSG mice ($n = 5$/group). The mice were treated daily with vehicle, BRL-50481 (10 mg/kg body weight), BAY-2402234 (0.5 mg/kg body weight), or the combination of BRL-50481 (10 mg/kg body weight) and BAY-2402234 (0.5 mg/kg body weight) and analyzed for tumor growth. Tumor growth was measured via weekly bioluminescence imaging. Representative whole-body bioluminescence images at the indicated weeks are shown.

(D) Whole-body bioluminescence intensities at the indicated weeks for the experiment shown in (C) are shown ($n = 5$/group).

(E) Lungs and livers were collected and imaged at the end of the experiment shown in (C).

(F) Bioluminescence intensities for the lungs and livers for images in the (E) are shown ($n = 5$/group).

(G) A model summarizing the role of PDE7A in TNBC.

All quantitative data represent the mean ± SEM. ns, not significant p value; *$p < 0.05$, **$p < 0.01$, ***$p < 0.001$, and ****$p < 0.0001$.

performed the TNBC IHC sample analysis. Y.J.K.E. performed the bioinformatics analysis for the RNA-seq data and submitted data to GEO. P.M., S. B., R.G., and N.W. wrote the manuscript. All authors have read and approved the final version of the manuscript.

DECLARATION OF INTERESTS

The authors declare no competing interests.

STAR★METHODS

Detailed methods are provided in the online version of this paper and include the following:

- KEY RESOURCES TABLE
- EXPERIMENTAL MODEL AND STUDY PARTICIPANT DETAILS
  - Cell culture
  - Experimental mice
  - Mouse tumor growth and metastasis experiments
  - Orthotopic injection of TNBC cells in the mouse mammary fat pad
  - Mouse tumorigenesis experiment using TNBC PDX models to test PDE7A inhibitor BRL-50481
  - Mouse tumorigenesis experiment using TNBC PDX models to test the combination of PDE7A inhibitor BRL-50481 and DHODH inhibitor BAY-2402234
- METHOD DETAILS
  - Chemical inhibitors
  - Plasmids
  - shRNAs and lentivirus generation
  - CRISPR knockout of PDE7A with single-guide RNA (sgRNAs)
  - MTT assay
  - Clonogenic assay
  - Soft-agar assay
  - CytoSelect 96-well quantitative soft agar assay
  - Matrigel invasion assay
  - Wound-healing assay
  - Caspase 3 activity assay
  - Measurement of cAMP levels
  - RNA preparation, complementary DNA (cDNA) preparation, reverse transcription (RT), and quantitative PCR (qPCR) analysis
  - Analysis of expression of various cyclic nucleotide phosphodiesterases (PDEs) and other genes in TCGA breast cancer dataset using UALCAN
  - PDE7A expression analysis in TNBC gene expression datasets
  - PDE7A protein expression in breast cancer samples from the Human Protein Tissue Atlas
  - Bioinformatics analysis of transcription factor binding on PDE7A promoter and correlation analysis
  - Bioinformatics analysis of E2F1 binding on DHODH promoter
  - Cleavage under targets & release using nuclease (CUT-&-RUN) assay
  - RNA sequencing and data analysis
  - Immunoblot analysis
  - Immunohistochemistry (IHC)
  - PDE7A mRNA stability assay
  - PDE7A protein degradation assay using MG132
  - RNA pol II CTD phospho Ser2 antibody ChIP on PDE7A promoter
  - Global metabolomic analysis
  - Analysis of key metabolites of pyrimidine biosynthesis pathway
- QUANTIFICATION AND STATISTICAL ANALYSIS

SUPPLEMENTAL INFORMATION

Supplemental information can be found online at https://doi.org/10.1016/j.xcrm.2025.102356.

Received: October 29, 2024
Revised: July 1, 2025
Accepted: August 18, 2025
Published: September 16, 2025

REFERENCES

1. Harbeck, N., Penault-Llorca, F., Cortes, J., Gnant, M., Houssami, N., Poortmans, P., Ruddy, K., Tsang, J., and Cardoso, F. (2019). Breast cancer. Nat. Rev. Dis. Primers *5*, 66. https://doi.org/10.1038/s41572-019-0111-2.
2. Russnes, H.G., Lingjærde, O.C., Børresen-Dale, A.L., and Caldas, C. (2017). Breast Cancer Molecular Stratification: From Intrinsic Subtypes to Integrative Clusters. Am. J. Pathol. *187*, 2152–2162. https://doi.org/10.1016/j.ajpath.2017.04.022.
3. Nolan, E., Lindeman, G.J., and Visvader, J.E. (2023). Deciphering breast cancer: from biology to the clinic. Cell *186*, 1708–1728. https://doi.org/10.1016/j.cell.2023.01.040.
4. Bianchini, G., Balko, J.M., Mayer, I.A., Sanders, M.E., and Gianni, L. (2016). Triple-negative breast cancer: challenges and opportunities of a heterogeneous disease. Nat. Rev. Clin. Oncol. *13*, 674–690. https://doi.org/10.1038/nrclinonc.2016.66.
5. Bianchini, G., De Angelis, C., Licata, L., and Gianni, L. (2022). Treatment landscape of triple-negative breast cancer - expanded options, evolving needs. Nat. Rev. Clin. Oncol. *19*, 91–113. https://doi.org/10.1038/s41571-021-00565-2.
6. Grasset, E.M., Dunworth, M., Sharma, G., Loth, M., Tandurella, J., Cimino-Mathews, A., Gentz, M., Bracht, S., Haynes, M., Fertig, E.J., and Ewald, A. J. (2022). Triple-negative breast cancer metastasis involves complex epithelial-mesenchymal transition dynamics and requires vimentin. Sci. Transl. Med. *14*, eabn7571. https://doi.org/10.1126/scitranslmed.abn7571.
7. Han, H.S., Vikas, P., Costa, R.L.B., Jahan, N., Taye, A., and Stringer-Reasor, E.M. (2023). Early-Stage Triple-Negative Breast Cancer Journey: Beginning, End, and Everything in Between. Am. Soc. Clin. Oncol. Educ. Book. *43*, e390464. https://doi.org/10.1200/EDBK_390464.
8. Cortes, J., Rugo, H.S., Cescon, D.W., Im, S.A., Yusof, M.M., Gallardo, C., Lipatov, O., Barrios, C.H., Perez-Garcia, J., Iwata, H., et al. (2022). Pembrolizumab plus Chemotherapy in Advanced Triple-Negative Breast Cancer. N. Engl. J. Med. *387*, 217–226. https://doi.org/10.1056/NEJMoa2202809.
9. Anders, C.K., Winer, E.P., Ford, J.M., Dent, R., Silver, D.P., Sledge, G.W., and Carey, L.A. (2010). Poly(ADP-Ribose) polymerase inhibition: "targeted" therapy for triple-negative breast cancer. Clin. Cancer Res. *16*, 4702–4710. https://doi.org/10.1158/1078-0432.CCR-10-0939.
10. DeBerardinis, R.J., and Chandel, N.S. (2016). Fundamentals of cancer metabolism. Sci. Adv. *2*, e1600200. https://doi.org/10.1126/sciadv.1600200.
11. Stine, Z.E., Schug, Z.T., Salvino, J.M., and Dang, C.V. (2022). Targeting cancer metabolism in the era of precision oncology. Nat. Rev. Drug Discov. *21*, 141–162. https://doi.org/10.1038/s41573-021-00339-6.
12. Nagarajan, A., Malvi, P., and Wajapeyee, N. (2016). Oncogene-directed alterations in cancer cell metabolism. Trends Cancer *2*, 365–377. https://doi.org/10.1016/j.trecan.2016.06.002.
13. Colegio, O.R., Chu, N.Q., Szabo, A.L., Chu, T., Rhebergen, A.M., Jairam, V., Cyrus, N., Brokowski, C.E., Eisenbarth, S.C., Phillips, G.M., et al. (2014). Functional polarization of tumour-associated macrophages by tumour-derived lactic acid. Nature *513*, 559–563. https://doi.org/10.1038/nature13490.
14. Roshanzamir, F., Robinson, J.L., Cook, D., Karimi-Jafari, M.H., and Nielsen, J. (2022). Metastatic triple negative breast cancer adapts its metabolism to destination tissues while retaining key metabolic signatures. Proc. Natl. Acad. Sci. USA *119*, e2205456119. https://doi.org/10.1073/pnas.2205456119.

CellPress
OPEN ACCESS

15. Vander Heiden, M.G., and DeBerardinis, R.J. (2017). Understanding the Intersections between Metabolism and Cancer Biology. Cell *168*, 657–669. https://doi.org/10.1016/j.cell.2016.12.039.

16. Zaal, E.A., and Berkers, C.R. (2018). The Influence of Metabolism on Drug Response in Cancer. Front. Oncol. *8*, 500. https://doi.org/10.3389/fonc.2018.00500.

17. Luengo, A., Gui, D.Y., and Vander Heiden, M.G. (2017). Targeting Metabolism for Cancer Therapy. Cell Chem. Biol. *24*, 1161–1180. https://doi.org/10.1016/j.chembiol.2017.08.028.

18. Newton, A.C., Bootman, M.D., and Scott, J.D. (2016). Second Messengers. Cold Spring Harbor Perspect. Biol. *8*, a005926. https://doi.org/10.1101/cshperspect.a005926.

19. Cardinale, A., and Fusco, F.R. (2018). Inhibition of phosphodiesterases as a strategy to achieve neuroprotection in Huntington's disease. CNS Neurosci. Ther. *24*, 319–328. https://doi.org/10.1111/cns.12834.

20. Ahmad, F., Murata, T., Shimizu, K., Degerman, E., Maurice, D., and Manganiello, V. (2015). Cyclic nucleotide phosphodiesterases: important signaling modulators and therapeutic targets. Oral Dis. *21*, e25–e50. https://doi.org/10.1111/odi.12275.

21. Essayan, D.M. (2001). Cyclic nucleotide phosphodiesterases. J. Allergy Clin. Immunol. *108*, 671–680. https://doi.org/10.1067/mai.2001.119555.

22. Conti, M. (2000). Phosphodiesterases and cyclic nucleotide signaling in endocrine cells. Mol. Endocrinol. *14*, 1317–1327. https://doi.org/10.1210/mend.14.9.0534.

23. Bolger, G.B. (2021). The PDE-Opathies: Diverse Phenotypes Produced by a Functionally Related Multigene Family. Trends Genet. *37*, 669–681. https://doi.org/10.1016/j.tig.2021.03.002.

24. Epstein, P.M. (2017). Different phosphodiesterases (PDEs) regulate distinct phosphoproteomes during cAMP signaling. Proc. Natl. Acad. Sci. USA *114*, 7741–7743. https://doi.org/10.1073/pnas.1709073114.

25. Azevedo, M.F., Faucz, F.R., Bimpaki, E., Horvath, A., Levy, I., de Alexandre, R.B., Ahmad, F., Manganiello, V., and Stratakis, C.A. (2014). Clinical and molecular genetics of the phosphodiesterases (PDEs). Endocr. Rev. *35*, 195–233. https://doi.org/10.1210/er.2013-1053.

26. Bloom, T.J., and Beavo, J.A. (1996). Identification and tissue-specific expression of PDE7 phosphodiesterase splice variants. Proc. Natl. Acad. Sci. USA *93*, 14188–14192. https://doi.org/10.1073/pnas.93.24.14188.

27. Maurice, D.H., Ke, H., Ahmad, F., Wang, Y., Chung, J., and Manganiello, V.C. (2014). Advances in targeting cyclic nucleotide phosphodiesterases. Nat. Rev. Drug Discov. *13*, 290–314. https://doi.org/10.1038/nrd4228.

28. Keravis, T., and Lugnier, C. (2012). Cyclic nucleotide phosphodiesterase (PDE) isozymes as targets of the intracellular signalling network: benefits of PDE inhibitors in various diseases and perspectives for future therapeutic developments. Br. J. Pharmacol. *165*, 1288–1305. https://doi.org/10.1111/j.1476-5381.2011.01729.x.

29. Hao, L., and Yu, H. (2017). MiR-23b inhibits cell migration and invasion through targeting PDE7A in colon cancer cells. Int. J. Clin. Exp. Pathol. *10*, 9436–9443.

30. Peng, T., Gong, J., Jin, Y., Zhou, Y., Tong, R., Wei, X., Bai, L., and Shi, J. (2018). Inhibitors of phosphodiesterase as cancer therapeutics. Eur. J. Med. Chem. *150*, 742–756. https://doi.org/10.1016/j.ejmech.2018.03.046.

31. You, J.S., and Jones, P.A. (2012). Cancer genetics and epigenetics: two sides of the same coin? Cancer Cell *22*, 9–20. https://doi.org/10.1016/j.ccr.2012.06.008.

32. Khan, M.A., Jain, V.K., Rizwanullah, M., Ahmad, J., and Jain, K. (2019). PI3K/AKT/mTOR pathway inhibitors in triple-negative breast cancer: a review on drug discovery and future challenges. Drug Discov. Today *24*, 2181–2191. https://doi.org/10.1016/j.drudis.2019.09.001.

33. Pascual, J., and Turner, N.C. (2019). Targeting the PI3-kinase pathway in triple-negative breast cancer. Ann. Oncol. *30*, 1051–1060. https://doi.org/10.1093/annonc/mdz133.

34. Garrido-Castro, A.C., Saura, C., Barroso-Sousa, R., Guo, H., Ciruelos, E., Bermejo, B., Gavilá, J., Serra, V., Prat, A., Paré, L., et al. (2020). Phase 2 study of buparlisib (BKM120), a pan-class I PI3K inhibitor, in patients with metastatic triple-negative breast cancer. Breast Cancer Res. *22*, 120. https://doi.org/10.1186/s13058-020-01354-y.

35. Zhao, J.J., Liu, Z., Wang, L., Shin, E., Loda, M.F., and Roberts, T.M. (2005). The oncogenic properties of mutant p110alpha and p110beta phosphatidylinositol 3-kinases in human mammary epithelial cells. Proc. Natl. Acad. Sci. USA *102*, 18443–18448. https://doi.org/10.1073/pnas.0508988102.

36. Ahn, S.H., Kim, M., and Buratowski, S. (2004). Phosphorylation of serine 2 within the RNA polymerase II C-terminal domain couples transcription and 3' end processing. Mol. Cell *13*, 67–76. https://doi.org/10.1016/s1097-2765(03)00492-1.

37. Hsin, J.P., and Manley, J.L. (2012). The RNA polymerase II CTD coordinates transcription and RNA processing. Genes Dev. *26*, 2119–2137. https://doi.org/10.1101/gad.200303.112.

38. Messeguer, X., Escudero, R., Farré, D., Núñez, O., Martínez, J., and Albà, M.M. (2002). PROMO: detection of known transcription regulatory elements using species-tailored searches. Bioinformatics *18*, 333–334. https://doi.org/10.1093/bioinformatics/18.2.333.

39. Farre, D., Roset, R., Huerta, M., Adsuara, J.E., Rosello, L., Alba, M.M., and Messeguer, X. (2003). Identification of patterns in biological sequences at the ALGGEN server: PROMO and MALGEN. Nucleic Acids Res. *31*, 3651–3653. https://doi.org/10.1093/nar/gkg605.

40. Smith, S.J., Cieslinski, L.B., Newton, R., Donnelly, L.E., Fenwick, P.S., Nicholson, A.G., Barnes, P.J., Barnette, M.S., and Giembycz, M.A. (2004). Discovery of BRL 50481 [3-(N,N-dimethylsulfonamido)-4-methyl-nitrobenzene], a selective inhibitor of phosphodiesterase 7: in vitro studies in human monocytes, lung macrophages, and CD8+ T-lymphocytes. Mol. Pharmacol. *66*, 1679–1689. https://doi.org/10.1124/mol.104.002246.

41. Naqvi, S., Martin, K.J., and Arthur, J.S.C. (2014). CREB phosphorylation at Ser133 regulates transcription via distinct mechanisms downstream of cAMP and MAPK signalling. Biochem. J. *458*, 469–479. https://doi.org/10.1042/BJ20131115.

42. Borowicz, S., Van Scoyk, M., Avasarala, S., Karuppusamy Rathinam, M.K., Tauler, J., Bikkavilli, R.K., and Winn, R.A. (2014). The soft agar colony formation assay. J. Vis. Exp. e51998. https://doi.org/10.3791/51998.

43. Dobrolecki, L.E., Airhart, S.D., Alferez, D.G., Aparicio, S., Behbod, F., Bentires-Alj, M., Brisken, C., Bult, C.J., Cai, S., Clarke, R.B., et al. (2016). Patient-derived xenograft (PDX) models in basic and translational breast cancer research. Cancer Metastasis Rev. *35*, 547–573. https://doi.org/10.1007/s10555-016-9653-x.

44. Lai, Y., Wei, X., Lin, S., Qin, L., Cheng, L., and Li, P. (2017). Current status and perspectives of patient-derived xenograft models in cancer research. J. Hematol. Oncol. *10*, 106. https://doi.org/10.1186/s13045-017-0470-7.

45. Walter, M., and Herr, P. (2022). Re-Discovery of Pyrimidine Salvage as Target in Cancer Therapy. Cells *11*, 739. https://doi.org/10.3390/cells11040739.

46. Okesli, A., Khosla, C., and Bassik, M.C. (2017). Human pyrimidine nucleotide biosynthesis as a target for antiviral chemotherapy. Curr. Opin. Biotechnol. *48*, 127–134. https://doi.org/10.1016/j.copbio.2017.03.010.

47. Shi, D.D., Savani, M.R., Levitt, M.M., Wang, A.C., Endress, J.E., Bird, C.E., Buehler, J., Stopka, S.A., Regan, M.S., Lin, Y.F., et al. (2022). De novo pyrimidine synthesis is a targetable vulnerability in IDH mutant glioma. Cancer Cell *40*, 939–956.e16. https://doi.org/10.1016/j.ccell.2022.07.011.

48. Pal, S., Kaplan, J.P., Nguyen, H., Stopka, S.A., Savani, M.R., Regan, M.S., Nguyen, Q.D., Jones, K.L., Moreau, L.A., Peng, J., et al. (2022). A druggable addiction to de novo pyrimidine biosynthesis in diffuse midline glioma. Cancer Cell *40*, 957–972.e10. https://doi.org/10.1016/j.ccell.2022.07.012.

49. Kim, J., Hu, Z., Cai, L., Li, K., Choi, E., Faubert, B., Bezwada, D., Rodriguez-Canales, J., Villalobos, P., Lin, Y.F., et al. (2017). CPS1 maintains

pyrimidine pools and DNA synthesis in KRAS/LKB1-mutant lung cancer cells. Nature *546*, 168–172. https://doi.org/10.1038/nature22359.

50. Wang, W., Cui, J., Ma, H., Lu, W., and Huang, J. (2021). Targeting pyrimidine metabolism in the era of precision cancer medicine. Front. Oncol. *11*, 684961. https://doi.org/10.3389/fonc.2021.684961.

51. Muehler, A., Kohlhof, H., Groeppel, M., and Vitt, D. (2019). The selective oral Immunomodulator vidofludimus in patients with active rheumatoid arthritis: safety results from the COMPONENT study. Drugs R&D *19*, 351–366. https://doi.org/10.1007/s40268-019-00286-z.

52. Zhou, J., Yiying Quah, J., Ng, Y., Chooi, J.Y., Hui-Min Toh, S., Lin, B., Zea Tan, T., Hosoi, H., Osato, M., Seet, Q., et al. (2020). ASLAN003, a potent dihydroorotate dehydrogenase inhibitor for differentiation of acute myeloid leukemia. Haematologica *105*, 2286–2297. https://doi.org/10.3324/haematol.2019.230482.

53. Sainas, S., Pippione, A.C., Lupino, E., Giorgis, M., Circosta, P., Gaidano, V., Goyal, P., Bonanni, D., Rolando, B., Cignetti, A., et al. (2018). Targeting Myeloid Differentiation Using Potent 2-Hydroxypyrazolo[1,5- a]pyridine Scaffold-Based Human Dihydroorotate Dehydrogenase Inhibitors. J. Med. Chem. *61*, 6034–6055. https://doi.org/10.1021/acs.jmedchem.8b00373.

54. Zhou, Y., Tao, L., Zhou, X., Zuo, Z., Gong, J., Liu, X., Zhou, Y., Liu, C., Sang, N., Liu, H., et al. (2021). DHODH and cancer: promising prospects to be explored. Cancer Metabol. *9*, 22. https://doi.org/10.1186/s40170-021-00250-z.

55. Christian, S., Merz, C., Evans, L., Gradl, S., Seidel, H., Friberg, A., Eheim, A., Lejeune, P., Brzezinka, K., Zimmermann, K., et al. (2019). The novel dihydroorotate dehydrogenase (DHODH) inhibitor BAY 2402234 triggers differentiation and is effective in the treatment of myeloid malignancies. Leukemia *33*, 2403–2415. https://doi.org/10.1038/s41375-019-0461-5.

56. Sykes, D.B., Kfoury, Y.S., Mercier, F.E., Wawer, M.J., Law, J.M., Haynes, M.K., Lewis, T.A., Schajnovitz, A., Jain, E., Lee, D., et al. (2016). Inhibition of dihydroorotate dehydrogenase overcomes differentiation blockade in acute myeloid leukemia. Cell *167*, 171–186.e15. https://doi.org/10.1016/j.cell.2016.08.057.

57. Jin, H., Wang, L., and Bernards, R. (2023). Rational combinations of targeted cancer therapies: background, advances and challenges. Nat. Rev. Drug Discov. *22*, 213–234. https://doi.org/10.1038/s41573-022-00615-z.

58. Long, G.V., Stroyakovskiy, D., Gogas, H., Levchenko, E., de Braud, F., Larkin, J., Garbe, C., Jouary, T., Hauschild, A., Grob, J.J., et al. (2014). Combined BRAF and MEK inhibition versus BRAF inhibition alone in melanoma. N. Engl. J. Med. *371*, 1877–1888. https://doi.org/10.1056/NEJMoa1406037.

59. Tricker, E.M., Xu, C., Uddin, S., Capelletti, M., Ercan, D., Ogino, A., Pratilas, C.A., Rosen, N., Gray, N.S., Wong, K.K., and Jänne, P.A. (2015). Combined EGFR/MEK inhibition prevents the emergence of resistance in EGFR-mutant lung cancer. Cancer Discov. *5*, 960–971. https://doi.org/10.1158/2159-8290.CD-15-0063.

60. Browne, I.M., André, F., Chandarlapaty, S., Carey, L.A., and Turner, N.C. (2024). Optimal targeting of PI3K-AKT and mTOR in advanced oestrogen receptor-positive breast cancer. Lancet Oncol. *25*, e139–e151. https://doi.org/10.1016/S1470-2045(23)00676-9.

61. Amalia, E., Diantini, A., Endang Prabandari, E., Waluyo, D., and Subarnas, A. (2022). Caffeic Acid Phenethyl Ester as a DHODH Inhibitor and Its Synergistic Anticancer Properties in Combination with 5-Fluorouracil in a Breast Cancer Cell Line. J. Exp. Pharmacol. *14*, 243–253. https://doi.org/10.2147/JEP.S365159.

62. Hosseini, M., Dousset, L., Michon, P., Mahfouf, W., Muzotte, E., Bergeron, V., Bortolotto, D., Rossignol, R., Moisan, F., Taieb, A., et al. (2019). UVB-induced DHODH upregulation, which is driven by STAT3, is a promising target for chemoprevention and combination therapy of photocarcinogenesis. Oncogenesis *8*, 52. https://doi.org/10.1038/s41389-019-0161-z.

63. Pizzorno, G., Wiegand, R.A., Lentz, S.K., and Handschumacher, R.E. (1992). Brequinar potentiates 5-fluorouracil antitumor activity in a murine model colon 38 tumor by tissue-specific modulation of uridine nucleotide pools. Cancer Res. 52, 1660–1665.

64. Cheng, R.Y.S., Patel, N.L., Back, T., Basudhar, D., Somasundaram, V., Kalen, J.D., Wink, D.A., and Ridnour, L.A. (2020). Studying Triple Negative Breast Cancer Using Orthotopic Breast Cancer Model. J. Vis. Exp. https://doi.org/10.3791/60316.

65. Borneman, R.M., Gavin, E., Musiyenko, A., Richter, W., Lee, K.J., Crossman, D.K., Andrews, J.F., Wilhite, A.M., McClellan, S., Aragon, I., et al. (2022). Phosphodiesterase 10A (PDE10A) as a novel target to suppress beta-catenin and RAS signaling in epithelial ovarian cancer. J. Ovarian Res. *15*, 120. https://doi.org/10.1186/s13048-022-01050-9.

66. Hao, N., Shen, W., Du, R., Jiang, S., Zhu, J., Chen, Y., Huang, C., Shi, Y., Xiang, R., and Luo, Y. (2020). Phosphodiesterase 3A Represents a Therapeutic Target that Drives Stem Cell-like Property and Metastasis in Breast Cancer. Mol. Cancer Therapeut. *19*, 868–881. https://doi.org/10.1158/1535-7163.MCT-18-1233.

67. Kim, D.U., Nam, J., Cha, M.D., and Kim, S.W. (2019). Inhibition of phosphodiesterase 4D decreases the malignant properties of DLD-1 colorectal cancer cells by repressing the AKT/mTOR/Myc signaling pathway. Oncol. Lett. *17*, 3589–3598. https://doi.org/10.3892/ol.2019.9996.

68. Moffatt, B.A., and Ashihara, H. (2002). Purine and pyrimidine nucleotide synthesis and metabolism. Arabidopsis Book *1*, e0018. https://doi.org/10.1199/tab.0018.

69. Villa, E., Ali, E.S., Sahu, U., and Ben-Sahra, I. (2019). Cancer Cells Tune the Signaling Pathways to Empower de Novo Synthesis of Nucleotides. Cancers (Basel) *11*, 688. https://doi.org/10.3390/cancers11050688.

70. Ben-Sahra, I., Howell, J.J., Asara, J.M., and Manning, B.D. (2013). Stimulation of de novo pyrimidine synthesis by growth signaling through mTOR and S6K1. Science *339*, 1323–1328. https://doi.org/10.1126/science.1228792.

71. Loffler, M., Fairbanks, L.D., Zameitat, E., Marinaki, A.M., and Simmonds, H.A. (2005). Pyrimidine pathways in health and disease. Trends Mol. Med. *11*, 430–437. https://doi.org/10.1016/j.molmed.2005.07.003.

72. Wu, H.L., Gong, Y., Ji, P., Xie, Y.F., Jiang, Y.Z., and Liu, G.Y. (2022). Targeting nucleotide metabolism: a promising approach to enhance cancer immunotherapy. J. Hematol. Oncol. *15*, 45. https://doi.org/10.1186/s13045-022-01263-x.

73. Gwynne, W.D., Suk, Y., Custers, S., Mikolajewicz, N., Chan, J.K., Zador, Z., Chafe, S.C., Zhai, K., Escudero, L., Zhang, C., et al. (2022). Cancer-selective metabolic vulnerabilities in MYC-amplified medulloblastoma. Cancer Cell *40*, 1488–1502.e7. https://doi.org/10.1016/j.ccell.2022.10.009.

74. Flaherty, K.T., Infante, J.R., Daud, A., Gonzalez, R., Kefford, R.F., Sosman, J., Hamid, O., Schuchter, L., Cebon, J., Ibrahim, N., et al. (2012). Combined BRAF and MEK inhibition in melanoma with BRAF V600 mutations. N. Engl. J. Med. *367*, 1694–1703. https://doi.org/10.1056/NEJMoa1210093.

75. Ercan, D., Xu, C., Yanagita, M., Monast, C.S., Pratilas, C.A., Montero, J., Butaney, M., Shimamura, T., Sholl, L., Ivanova, E.V., et al. (2012). Reactivation of ERK signaling causes resistance to EGFR kinase inhibitors. Cancer Discov. *2*, 934–947. https://doi.org/10.1158/2159-8290.CD-12-0103.

76. Lehmann, B.D., Bauer, J.A., Chen, X., Sanders, M.E., Chakravarthy, A.B., Shyr, Y., and Pietenpol, J.A. (2011). Identification of human triple-negative breast cancer subtypes and preclinical models for selection of targeted therapies. J. Clin. Investig. *121*, 2750–2767. https://doi.org/10.1172/JCI45014.

77. Ding, S., Wu, X., Li, G., Han, M., Zhuang, Y., and Xu, T. (2005). Efficient transposition of the piggyBac (PB) transposon in mammalian cells and mice. Cell *122*, 473–483. https://doi.org/10.1016/j.cell.2005.07.013.

78. Fang, J., Uchiumi, T., Yagi, M., Matsumoto, S., Amamoto, R., Saito, T., Takazaki, S., Kanki, T., Yamaza, H., Nonaka, K., and Kang, D. (2012). Protein instability and functional defects caused by mutations of dihydro-orotate dehydrogenase in Miller syndrome patients. Biosci. Rep. *32*, 631–639. https://doi.org/10.1042/BSR20120046.

79. Chandrashekar, D.S., Karthikeyan, S.K., Korla, P.K., Patel, H., Shovon, A. R., Athar, M., Netto, G.J., Qin, Z.S., Kumar, S., Manne, U., et al. (2022). UALCAN: An update to the integrated cancer data analysis platform. Neoplasia *25*, 18–27. https://doi.org/10.1016/j.neo.2022.01.001.

80. Ma, X.M., Han, Y.W., Zhang, J., Cui, W.J., and Wang, X. (2013). [Differences and clinical significance of receptor expression between primary and locally recurrent breast tumor tissues]. Zhonghua Zhongliu Zazhi *35*, 372–376. https://doi.org/10.3760/cma.j.issn.0253-3766.2013.05.011.

81. Karnoub, A.E., Dash, A.B., Vo, A.P., Sullivan, A., Brooks, M.W., Bell, G.W., Richardson, A.L., Polyak, K., Tubo, R., and Weinberg, R.A. (2007). Mesenchymal stem cells within tumour stroma promote breast cancer metastasis. Nature *449*, 557–563. https://doi.org/10.1038/nature06188.

82. Richardson, A.L., Wang, Z.C., De Nicolo, A., Lu, X., Brown, M., Miron, A., Liao, X., Iglehart, J.D., Livingston, D.M., and Ganesan, S. (2006). X chromosomal abnormalities in basal-like human breast cancer. Cancer Cell *9*, 121–132. https://doi.org/10.1016/j.ccr.2006.01.013.

83. Julka, P.K., Chacko, R.T., Nag, S., Parshad, R., Nair, A., Oh, D.S., Hu, Z., Koppiker, C.B., Nair, S., Dawar, R., et al. (2008). A phase II study of sequential neoadjuvant gemcitabine plus doxorubicin followed by gemcitabine plus cisplatin in patients with operable breast cancer: prediction of response using molecular profiling. Br. J. Cancer *98*, 1327–1335. https://doi.org/10.1038/sj.bjc.6604322.

84. Sorlie, T., Perou, C.M., Tibshirani, R., Aas, T., Geisler, S., Johnsen, H., Hastie, T., Eisen, M.B., van de Rijn, M., Jeffrey, S.S., et al. (2001). Gene expression patterns of breast carcinomas distinguish tumor subclasses with clinical implications. Proc. Natl. Acad. Sci. USA *98*, 10869–10874. https://doi.org/10.1073/pnas.191367098.

85. Esserman, L.J., Berry, D.A., Cheang, M.C.U., Yau, C., Perou, C.M., Carey, L., DeMichele, A., Gray, J.W., Conway-Dorsey, K., Lenburg, M.E., et al. (2012). Chemotherapy response and recurrence-free survival in neoadjuvant breast cancer depends on biomarker profiles: results from the I-SPY 1 TRIAL (CALGB 150007/150012; ACRIN 6657). Breast Cancer Res. Treat. *132*, 1049–1062. https://doi.org/10.1007/s10549-011-1895-2.

86. Curtis, C., Shah, S.P., Chin, S.F., Turashvili, G., Rueda, O.M., Dunning, M. J., Speed, D., Lynch, A.G., Samarajiwa, S., Yuan, Y., et al. (2012). The genomic and transcriptomic architecture of 2,000 breast tumours reveals novel subgroups. Nature *486*, 346–352. https://doi.org/10.1038/nature10983.

87. Cancer Genome Atlas Network (2012). Comprehensive molecular portraits of human breast tumours. Nature *490*, 61–70. https://doi.org/10.1038/nature11412.

88. Maston, G.A., Zhu, L.J., Chamberlain, L., Lin, L., Fang, M., and Green, M. R. (2012). Non-canonical TAF complexes regulate active promoters in human embryonic stem cells. eLife *1*, e00068. https://doi.org/10.7554/eLife.00068.

STAR★METHODS

KEY RESOURCES TABLE

REAGENT or RESOURCE	SOURCE	IDENTIFIER
Antibodies		
ACTINB	Cell signaling	Cat# 4970; RRID: AB_2223172
PDE7A (for WB)	Abcam	Cat# ab154857; RRID: AB_2889912
PDE7A (for IHC)	Sigma-Aldrich	HPA027340; RRID: AB_1855127
IRF1	Cell Signaling	Cat# 8478; RRID:AB_10949108
DHODH	Cell Signaling	Cat# 80981
CAD	Cell Signaling	Cat# 93925; RRID: AB_2750933
CREB	Cell Signaling	Cat# 4820; RRID: AB_1903940
Phospho-CREB	Cell Signaling	Cat# 9198; RRID: AB_2561044
p-AKT (Ser473)	Cell Signaling	Cat# 9271; RRID: AB_329825
AKT	Cell Signaling	Cat# 9272; RRID: AB_329827
V5-Tag	Cell Signaling	Cat# 13202; RRID: AB_2687461
GFP	Cell Signaling	Cat# 2956; RRID:AB_1196615
E2F1	Cell Signaling	Cat# 3742; RRID:AB_2096936
γ-H2AX	Cell Signaling	Cat# 9718; RRID:AB_2118009
PDE6A	Proteintech	Cat# 67832-I
P53	Santa Cruz Biotechnology	Cat# sc-126; RRID:AB_628082
RNA pol II CTD phospho Ser2	Active Motif	Cat# 61083; RRID: AB_2687450
Biological samples		
Breast Cancer Tissue Microarray	US Biomax, Inc.	Cat# BC081120f
Chemicals, peptides, and recombinant proteins		
DMEM	GIBCO	Cat# 11965-092
RPMI	GIBCO	Cat# 11875-093
Mammary Epithelial Cell Basal Medium	ATCC	Cat# PCS-600-030
MEGM Mammary Epithelial Cell Growth Medium	Lonza	Cat# CC-3150
Fetal Bovine Serum	GIBCO	Cat# 10437-028
Trypsin-EDTA	GIBCO	Cat# 25200-056
Trypsin inhibitor	Sigma-Aldrich	Cat# T6414
Penicillin-Streptomycin	GIBCO	Cat# 15140-122
Effectene Transfection Reagent	QIAGEN	Cat# 301427
Methylthiazole tetrazolium (MTT)	Sigma-Aldrich	Cat# M5655
Agarose, Low gelling	Sigma-Aldrich	Cat# A9045
XenoLight D-Luciferin - K+ Salt Bioluminescent Substrate	Perkin Elmer	Cat# 122799
Matrigel Basement Membrane Matrix	Corning	Cat# 356237
Matrigel Invasion Chambers	BD Biosciences	Cat# 354483
VECTASHIELD Hardset Antifade Mounting Medium with DAPI	Vector Laboratories	Cat# H-1500
BRL-50481	SelleckChem	Cat# S5837
BAY-2402234	SelleckChem	Cat# S8847
Buparlisib (BKM120)	SelleckChem	Cat# S2247
Uridine	Sigma-Aldrich	Cat# U3750
Actinomycin D	Sigma-Aldrich	Cat# A9415
MG-132	Sigma-Aldrich	Cat# 474790

(*Continued on next page*)

CellPress
OPEN ACCESS

Continued

REAGENT or RESOURCE	SOURCE	IDENTIFIER
5-Fluorouracil	Sigma-Aldrich	Cat# 343922
Doxorubicin	Cayman Chemical	Cat# 15007
Etoposide	Sigma-Aldrich	Cat# E1383
**Critical commercial assays**		
Cytoselect 96-well cell transformation assay	Cell Biolabs Inc.	Cat#CBA-130
Caspase 3 Assay Kit, Colorimetric	Sigma-Aldrich	Cat# CASP3C
cAMP Biotrak Enzyme immunoassay (EIA) System	GE Healthcare	Cat# RPN225
CUT&RUN Assay Kit	Cell Signaling	Cat#86652
QuikChange II Site-Directed Mutagenesis Kit	Agilent	Cat# 200523
**Deposited data**		
RNA-Seq performed with MDA-MB-231 cells treated with either DMSO or BRL-50481	This paper	Gene Expression Omnibus (GEO): GSE192830
Global metabolomic analysis performed with MDA-MB-231 cells treated with either DMSO or BRL-50481	This paper	National Metabolomics Data Repository (NMDR): ST004077
**Experimental models: Cell lines/organoids/PDXs**		
293T	ATCC	ATCC CRL-3216
MDA-MB-231	ATCC	ATCC HTB-26
MDA-MB-468	ATCC	ATCC HTB-132
BT-549	ATCC	ATCC HTB-122
MCF7	ATCC	ATCC HTB-22
T47D	ATCC	ATCC HTB-133
HCC1954	ATCC	ATCC CRL-2338
SK-BR-3	ATCC	ATCC HTB-30
hTERT-HME1	ATCC	ATCC CRL-4010
TNBC PDX	Jackson Laboratory	TM00096
TNBC PDX	Jackson Laboratory	TM00098
**Experimental models: Organisms/strains**		
Mouse: female NSG mice	Jackson Laboratory	Stock No. 005557
**Oligonucleotides**		
PDE7A shRNA#1	Sigma-Aldrich	TRC clone ID: TRCN0000048863
PDE7A shRNA#2	Sigma-Aldrich	TRC clone ID: TRCN0000048864
PDE6A shRNA#1	Sigma-Aldrich	TRC clone ID: TRCN0000008602
PDE6A shRNA#2	Sigma-Aldrich	TRC clone ID: TRCN0000008604
IRF1 shRNA#1	Sigma-Aldrich	TRC clone ID: TRCN0000014672
IRF1 shRNA#2	Sigma-Aldrich	TRC clone ID: TRCN0000077438
E2F1 shRNA#1	Sigma-Aldrich	TRC clone ID: TRCN0000039659
E2F1 shRNA#2	Sigma-Aldrich	TRC clone ID: TRCN0000039661
	Forward Primer	Reverse Primer
Human PDE7A	CGTATGCTAGGAGATGTACGTGT	TGAAACCGCAGTACCACGAAA
Human IRF1	CTGTGCGAGTGTACCGGATG	ATCCCCACATGACTTCCTCTT
Human DHODH	GTTCTGGGCCATAAATTCCGA	TCTGGGTCTAGGGTTTCCTTC
Human CAD	AGTGGTGTTTCAAACCGGCAT	CAGAGGATAGGTGAGCACTAAGA
Human ATF2	GCACAGCCCACATCAGCTATT	GGTGCCTGGGTGATTACAGT
Human CEBPB	CTTCAGCCCGTACCTGGAG	GGAGAGGAAGTCGTGGTGC
Human CREB1	ATTCACAGGAGTCAGTGGATAGT	CACCGTTACAGTGGTGATGG

(*Continued on next page*)

Continued

REAGENT or RESOURCE	SOURCE	IDENTIFIER
Human FOXP3	GTGGCCCGGATGTGAGAAG	GGAGCCCTTGTCGGATGATG
Human PAX5	ACTTGCTCATCAAGGTGTCAG	TCCTCCAATTACCCCAGGCTT
Human YY1	ACGGCTTCGAGGATCAGATTC	TGACCAGCGTTTGTTCAATGT
Human E2F1	GGACCTGGAAACTGACCATCAG	CAGTGAGGTCTCATAGCGTGAC
Human PDE6A	GTCCGTGCTTTCCTCAACTGTG	GGACCAGAGTAAGGTGGAACTTC
Human ACTINB	GTCTTCCCCTCCATCGTGGG	CCTCTCTTGCTCTGGGCCTC
PDE7A promoter CUT&RUN primers	TTAGCGCTCGGGGGGATGC	TTCCCTAGCTCCTCGCCAG
DHODH promoter CUT&RUN primers	TGCCACTACGCCCGGCTAAT	GGTCAACACCCAAAACCCCGT
ACTINB promoter CUT&RUN primers	TCTTGGCTGGGCGTGACTGT	AAGGTGGGCTCTACAGGGCA
NS sgRNA	CACCGAAAAAGCTTCCGCCTGATGG	AAACCCATCAGGCGGAAGCTTTTC
PDE7A sgRNA	CACCGCCATAACGCAGTCCACGCTG	AAACCAGCGTGGACTGCGTTATGGC
DHODH (R135C) mutagenesis primers	GACCCAGAGTCTTCTGCC TCCCTGAGGAC	GTCCTCAGGGAGGCAGA AGACTCTGGGTC
RNA pol II CTD phospho Ser2 ChIP primer for PDE7A gene	GAACTCTGCAGTCCGGGTAC	AATTACCAGGACCGGCGCG
**Recombinant DNA**		
Plasmid: piggyBac GFP-Luc	Ding et al.[77]	N/A
Plasmid: Act-PBase	Ding et al.[77]	N/A
Plasmid: psPAX2	Addgene	Cat# 12260, RRID:Addgene_12260
Plasmid: pMD2.G	Addgene	Cat# 12259, RRID:Addgene_12259
Plasmid: lentiCRISPR v2	Addgene	Cat# 52961, RRID:Addgene_52961
Plasmid: pLX304-V5-BLAST	Addgene	Cat# 25890; RRID:Addgene_25890
Plasmid: pLX304-*DHODH*-V5-BLAST	Horizon Discovery	Cat# OHS6269-213578631
Plasmid: pLX304-*PDE7A*-V5-BLAST	Horizon Discovery	Cat# OHS6085-213573825
Plasmid: Constitutively active PIK3CA (pBabe puro Myr HA *PIK3CA*)	Addgene	Cat# 12523, RRID:Addgene_12523
Plasmid: pBABE-puro	Addgene	Cat# 1764, RRID:Addgene_1764
Adenovirus: Ad-CMV-GFP	Vector Biolabs	Cat# 1060
Adenovirus: Ad-CMV-*p53* (GFP)	Vector Biolabs	Cat# 1260
Plasmid: *DHODH* mGFP-tagged Lenti ORF Clone	OriGene	Cat# RC209034L4
**Software and algorithms**		
Prism 10.0	GraphPad	www.graphpad.com/scientific software/prism
ImageJ	https://imagej.nih.gov/ij	N/A
PROMO 3.0	https://alggen.lsi.upc.es/cgi-bin/promo_v3/promo/promoinit.cgi?dirDB=TF_8.3	N/A
rVista 2.0	https://rvista.dcode.org	N/A

EXPERIMENTAL MODEL AND STUDY PARTICIPANT DETAILS

Cell culture

TNBC cell lines (MDA-MB-231, MDA-MB-468 and BT-549), non-TNBC [ER + cell lines (MCF7, and T47D) and HER2+ cell lines (HCC1954 and SK-BR-3)] and 293T cells were purchased from American Type Culture Collection (ATCC) and maintained in a humidified atmosphere containing 5% CO_2 at 37°C in Dulbecco's modified Eagle medium (Life Technologies, Carlsbad, CA, USA) or Roswell Park Memorial Institute (RPMI)-1640 medium (Life Technologies), supplemented with 10% fetal bovine serum (Life Technologies) and 1% penicillin/streptomycin (Life Technologies), as recommended. hTERT-HME1 cells were were purchased from ATCC, and maintained in a humidified atmosphere containing 5% CO_2 at 37°C in MEBM Basal Medium supplemented with bovine pituitary extract (BPE), human epidermal growth factor (hEGF), insulin and hydrocortisone. All cell lines were authenticated by ATCC using

using short tandem repeat (STR) markers analysis. All cell lines were also tested regularly using Lonza's MycoSEQ kit and confirmed to be negative before performing the experiments.

Experimental mice

For all the tumor growth– and/or metastasis–based experiments, 5–6-week-old female NSG mice (stock no. 005557; The Jackson Laboratory, Bar Harbor, ME, USA) were used. All the mice were housed and were maintained in accordance with the UAB's Institutional Animal Care and Use Committee (IACUC) guidelines. All protocols for these experiments were approved by the UAB's IACUC. All these experiments were performed in accordance with the IACUC guidelines.

Mouse tumor growth and metastasis experiments

Subcutaneous xenograft tumor growth experiments in mice for PDE7A inhibitor BRL-50481

5×10^6 MDA-MB-231 cells or 5×10^6 MDA-MB-468 cells or 5×10^6 BT-549 cells were injected subcutaneous into the dorsal flank of 5–6-week-old female NSG mice. When the average tumor volumes reached approximately 100 mm^3 in each experimental group, mice were treated with either vehicle (0.5% methyl cellulose) or BRL-50481 (25 mg/kg body weight) intraperitoneally every other day until the end of the experimental methods. Average tumor volume was measured and are reported in respective figures. Tumor volumes were calculated using the following formula: $length \times width^2 \times 0.5$. All protocols were approved by the UAB Institutional Animal Care and Use Committee.

Subcutaneous xenograft tumor growth experiments in mice for DHODH inhibitor BAY-2402234

5×10^6 MDA-MB-231 cells or 5×10^6 MDA-MB-468 cells or 5×10^6 BT-549 cells were injected subcutaneous into the dorsal flank of 5–6-week-old female NSG mice. When the average tumor volumes reached approximately 100 mm^3 in each experimental group mice, were treated with either vehicle (0.5% methyl cellulose) or BAY-2402234 (2 mg/kg body weight) intraperitoneally other day until the end of the experimental methods. Average tumor volume was measured and are reported in respective figures. Tumor volumes were calculated using the following formula: $length \times width^2 \times 0.5$. All protocols were approved by the UAB Institutional Animal Care and Use Committee.

Subcutaneous xenograft tumor growth experiments in mice for PDE7A knockout and DHODH *ORF–expressing TNBC cells*

5×10^6 MDA-MB-231 cells expressing NT sgRNA or *PDE7A* sgRNA with empty vector or *DHODH* ORF were injected subcutaneous into the dorsal flank of 5–6-week-old female NSG mice. Average tumor volume was measured and are reported in respective figures. Tumor volumes were calculated using the following formula: $length \times width^2 \times 0.5$. All protocols were approved by the UAB Institutional Animal Care and Use Committee.

Orthotopic injection of TNBC cells in the mouse mammary fat pad

MDA-MB-231 cells stably expressing firefly luciferase under the control of a cytomegalovirus promoter were generated by co-transfection of the transposon vector piggyBac GFP-Luc and the helper plasmid Act-PBase as described previously.[77] Cells with stable transposon integration were selected using blasticidin S (Thermo-Fisher Scientific, Waltham, MA, USA). MDA-MB-231-*F-Luc* cells (2.5×10^5) (suspended in Matrigel, 1:1 in 35 μL of PBS solution) were orthotopically into the mammary fat pad of 5–6-week-old NSG female mice (stock no. 005557, Jackson Laboratory). Imaging was performed every week until the completion of the experiment by injecting mice with D-luciferin using the Spectrum *In Vivo* Imaging System (PerkinElmer, Waltham, MA, USA). Total luminescence counts of the tumor-bearing areas were measured using the Living Image *in vivo* imaging software (PerkinElmer). After tumor became palpable, mice were treated with vehicle (0.5% methyl cellulose in water) or BRL-50481 (10 mg/kg body weight) or BAY-2402234 (0.5 mg/kg body weight) or combination of BRL-50481 (10 mg/kg body weight) and BAY-2402234 (0.5 mg/kg body weight) intraperitoneally every other day until the end of the experimental period. At the endpoint all the animals were subjected to imaging of whole-body, lung and liver to monitor the metastasis in liver and lungs. The bioluminescence intensity at week-2 and at week-5 was plotted. All protocols were approved by the UAB Institutional Animal Care and Use Committee.

Mouse tumorigenesis experiment using TNBC PDX models to test PDE7A inhibitor BRL-50481

TNBC PDXs (stock no. TM00096 and TM00098- Jackson Laboratory) were obtained in donor NSG PDX-engrafted mice. After 6–8 weeks, the PDXs were harvested, and implanted into 5–6-week-old female NSG mice (stock no. 005557, Jackson Laboratory). In brief, PDXs tissues were minced to a size of 2 mm × 2 mm and subcutaneously implanted into the right flank of female NSG mice. Tumor volume was measured every week. When the tumor volumes reached 80–100 mm^3, the mice were treated with vehicle (0.5% methyl cellulose) and BRL-50481 (25 mg/kg body weight) intraperitoneally every other day until the end of the experimental period. Tumor volume was measured every week and plotted. Subcutaneous tumors from individual groups were harvested and imaged. All protocols were approved by the UAB Institutional Animal Care and Use Committee.

Mouse tumorigenesis experiment using TNBC PDX models to test the combination of PDE7A inhibitor BRL-50481 and DHODH inhibitor BAY-2402234

TNBC PDXs (stock no. TM00096 and TM00098- Jackson Laboratory) were obtained in donor NSG PDX-engrafted mice. After 6–8 weeks, the PDXs were harvested, and implanted into 5–6-week-old female NSG mice (stock no. 005557, Jackson Laboratory).

In brief, PDXs tissues were minced to a size of 2 mm × 2 mm and subcutaneously implanted into the right flank of female NSG mice. Tumor volume was measured every week. When the tumor volumes reached 80–100 mm^3, the mice treated with vehicle (0.5% methyl cellulose in water) or BRL-50481 (10 mg/kg body weight) or BAY-2402234 (0.5 mg/kg body weight) or combination of BRL-50481 (10 mg/kg body weight) or BAY-2402234 (0.5 mg/kg body weight) intraperitoneally every other day until the end of the experimental period. Tumor volume was measured every week and plotted. Subcutaneous tumors from individual groups were harvested and imaged. All protocols were approved by the UAB Institutional Animal Care and Use Committee.

METHOD DETAILS

Chemical inhibitors

The PDE7A inhibitor BRL-50481, DHODH inhibitor BAY-2402234, and PI3K inhibitor buparlisib were purchased from Selleckchem and dissolved for cell culture and *in vivo* experiments as recommended in the datasheet. Actinomycin D, MG-132, 5-Fluorouracil, Doxorubicin and Etoposide were purchased from Sigma-Aldrich and dissolved for cell culture experiments. Relevant information is provided in key resources table. The treatment conditions are described in the corresponding figure legends.

Plasmids

The V5-tagged DHODH lentiviral expression construct in the pLX304-Blast-V5 plasmid purchased from Horizon Discovery (Waterbeach, UK). The lentiviral empty vector (pLX304-Blast-V5) plasmid was purchased from Addgene (Watertown, MA, USA). The Constitutively active PIK3CA plasmid was purchased from Addgene (Watertown, MA, USA). The mGFP-tagged *DHODH* Lenti ORF Clone was purchased from OriGene (Rockville, MD, USA). We performed site-directed mutagenesis to generate *DHODH* R135C mutant, which abolishes the catalytic activity of DHODH,[78] using a QuikChange II Site-Directed Mutagenesis Kit from Agilent (Santa Clara, CA, USA) per the manufacturer's instructions. Gene-specific lentiviral short hairpin RNAs (shRNAs) were obtained from Horizon Discovery (Waterbeach, UK). The details of the plasmids are listed in the key resources table.

shRNAs and lentivirus generation

Horizon Discovery supplied the pLKO.1 lentiviral vector-based shRNAs for specific target genes and non-specific (NS) control shRNA. The shRNAs are listed in the key resources table. As described in detail at https://portals.broadinstitute.org/gpp/public/resources/protocols, lentiviral particles were generated by co-transfecting 293T cells with gene-specific or NS shRNA plasmids and lentiviral packaging plasmids. Effectene Transfection Reagent (Qiagen, Hilden, Germany) was utilized for every lentiviral transduction. TNBC cells were infected with shRNA lentiviral particles in 12-well plates to generate stable cell lines. For cell selection, appropriate concentrations of puromycin (0.5–1.5 μg/mL) were used. For the pLX304-Blast-V5-based lentivirus, 2 μg/mL blasticidin (Thermo Fisher Scientific) was used to select successfully transduced TNBC cells.

CRISPR knockout of PDE7A with single-guide RNA (sgRNAs)

Gene-specific lentiviral *PDE7A* sgRNAs were cloned into the pLentiCRISPR v2 vector. The sgRNA sequences are provided in the key resources table. For lentivirus production, sgRNAs were transfected into 293T cells, along with the PDM2.G and psPAX2 packaging plasmids, using Effectene Transfection Reagent (Qiagen, Hilden, Germany). Stable cell lines were generated by infecting cells seeded in 12-well plates with sgRNA lentiviral particles, followed by selection with appropriate concentrations of puromycin (0.6 μg/mL) to enrich infected cells.

MTT assay

For this assay, 3×10^3 TNBC and non-TNBC cells were plated in a 100 μL volume in 96-well plates. After 24 h, inhibitors were added to 100 μL media at a range of concentrations as shown in figure and were then added to the cells. After 72 h of inhibitor treatment, cell viability was evaluated by adding 20 μL of 5 mg/mL MTT solution dissolved in 1× PBS to each well, followed by incubation for 1 h in a 37°C incubator. The MTT solution was gently removed, and 100 μL dimethyl sulfoxide was added to each well. After mixing by pipetting, absorbance was measured at 590 nm and 630 nm using the Biotek Synergy MX Multi-Format Microplate Reader (Winooski, VT, USA). The average measurement at 630 nm was subtracted from the average measurement at 590 nm, and the relative cell viability at each concentration was plotted with respect to vehicle-treated cells. For uridine supplementation-based MTT experiments, cells were plated and analyzed the same way as above except in case of uridine supplementation cells were grown in the presence of 100 μM uridine.

Clonogenic assay

Under DMSO or inhibitor-treated conditions, the clonogenic potential of TNBC cells was assessed. In 6-well culture plates, cells were seeded for these tests at 5×10^3 cells/well. The medium containing inhibitors was changed every 3 days. After 2 weeks of treatment, surviving colonies were stained with a solution of 40% methanol, 10% acetic acid, and 0.005% Coomassie Brilliant Blue R-250 (Sigma-Aldrich), and the plates were scanned with an Epson Perfection V850 Pro Photo Scanner (USA).

Soft-agar assay

Soft-agar assays were performed by seeding sgRNA- or open reading frame (ORF)-expressing or inhibitor-treated TNBC cells (10×10^3/well) as indicated in respective figure legends onto 0.4% low-melting-point agarose (Sigma-Aldrich) layered on top of 0.8% agarose. After 3–4 weeks of treatment colonies formed were imaged under a microscope and representative images are shown.

CytoSelect 96-well quantitative soft agar assay

Quantitative anchorage-independent cell growth assay (soft-agar assay) was also performed using the CytoSelect 96-well Cell Transformation Kit (Cell Biolabs, San Diego, CA, USA) according to the manufacturer's instructions. Briefly, shRNA-, sgRNA- or open reading frame (ORF)-expressing or inhibitor-treated TNBC cells as indicated in respective figure legends cells were seeded in 96-well plates (5×10^3 cells per well) in triplicates. After 7 days of incubation, culture medium was removed by inverting the plate and 50 μL of agar solubilization solution was added to each well of the 96-well plate and incubated for 1 h at 37°C. The agar mixture was incubated with a CyQuant working solution. Fluorescence was measured at 485/520 nm using a 96-well microplate reader and relative florescence unit (RFU) were plotted as a graph.

Matrigel invasion assay

Invasion assays were performed in BioCoat Growth Factor Reduced Matrigel Invasion Chambers (Cat #354483, Corning, Corning, NY, USA) in shRNA-, sgRNA- or open reading frame (ORF)-expressing or inhibitor-treated TNBC cells as indicated in respective figure legends. The cells were serum-starved for 6 h, and 5×10^4 cells/insert were seeded in triplicate in the top chamber containing low-serum medium. The cells were then incubated for 20 h to allow invasion toward the serum-rich medium in the bottom well. The number of cells invading the Matrigel was quantified by DAPI staining and imaging; 8–12 fields per membrane were counted, and nuclei quantification was performed using ImageJ software (NIH; https://imagej.nih.gov/ij/) and plotted.

Wound-healing assay

TNBC cell lines (MDA-MB-231, MDA-MB-468, and BT-549) were seeded in 6 well plate at a density of 2×10^5 cells per well and grown plates until fully confluent. A scratch was then created using a sterile 20-μL pipette tip, and the cell were then treated with DMSO or inhibitors at different concentration. Cell migration into the wound was monitored at 0, 24 and 48 h using light microscopy. Quantification of wound healing was performed using ImageJ software (https://imagej.nih.gov/ij/).

Caspase 3 activity assay

Caspase 3 activity assay was performed using Caspase-3 colorimetric assay kit (CASP3C, Sigma-Aldrich) as per the manufacturer's instructions in TNBC cells under conditions indicated in the respective figure legends. Caspase-3 activity assays were performed in 100 μL volume in a 96 well plate format using the Biotek Synergy MX Multi Format Microplate Reader (Biotek) and measurements were performed at 405 nm. The Caspase-3 Colorimetric Assay Kit is based on the hydrolysis of acetyl-Asp-Glu-Val-Asp *p*-nitroanilide (Ac-DEVD-pNA) by caspase-3, resulting in the release of the *p*-nitroaniline (pNA) moiety. *p*-Nitroaniline is detected at 405 nm using the Biotek Synergy MX Multi Format Microplate Reader (Biotek).

Measurement of cAMP levels

cAMP levels in TNBC and non-TNBC cells were measured using cAMP Biotrak enzyme immunoassay (EIA) kit system (GE Healthcare, IL, USA) under conditions indicated in the respective figure legends. This method uses the non-acetylation assay with the lysis reagents and combined amount of intracellular and cell supernatant cAMP level is measured. This fraction is referred to as 'total' cellular cAMP and has the additional benefit of not requiring decantation of the cell culture supernatant. cAMP is measured in the range 25–6400 fmol/well.

RNA preparation, complementary DNA (cDNA) preparation, reverse transcription (RT), and quantitative PCR (qPCR) analysis

Total RNA was extracted with TRIzol Reagent (Invitrogen, Carlsbad, CA, USA) and purified using the RNeasy Mini Kit (Qiagen, Hilden, Germany). Then, cDNA was generated using the M-MuLV First Strand cDNA Synthesis Kit (New England Biolabs, Ipswich, MA, USA) according to the manufacturer's instructions. Next, qPCR was performed with gene-specific primers using Power SYBR-Green Master Mix (Applied Biosystems, Foster City, CA, USA) according to the manufacturer's instructions. Beta actin (*ACTB*) levels were used as a normalization control. The primer sequences are provided in key resources table.

Analysis of expression of various cyclic nucleotide phosphodiesterases (PDEs) and other genes in TCGA breast cancer dataset using UALCAN

Datasets with gene expression data from TCGA breast cancer dataset for various PDE genes were analyzed in normal breast tissues ($n = 114$) and patient-derived TNBC samples ($n = 116$) using UALCAN (https://ualcan.path.uab.edu).[79] The fold change and *p*-value for the conditions indicated in the respective figures are plotted.

PDE7A expression analysis in TNBC gene expression datasets

Datasets with gene expression data from TNBC tissues and normal triple negative breast surface epithelium tissues were identified by searching the Oncomine cancer profiling database. The Ma Breast 4 [n = 14 for normal breast, and n = 11 for DCIS],[80] Karnoub Breast [n = 15 for normal breast, and n = 7 for IDBC],[81] Richardson Breast 2 [n = 7 for normal breast, and n = 40 for DBC],[82] Julka Breast [n = 8 for TNBC, and n = 31 for other biomarker status],[83] Sorlie Breast 2 [n = 23 for no recurrence at year-3, n = 24 for recurrence at year-3, n = 31 for alive at 3 years, and n = 32 for dead at 3 years],[84] Esserman breast [n = 21 for alive at 5 years, and n = 20 for dead at 5 years],[85] Curtis Breast [n = 211 for TNBC, and n = 1340 for other biomarker status],[86] Bittner Breast [n = 39 for TNBC, and n = 129 for other biomarker status] and TCGA Breast [n = 46 for TNBC, and n = 250 for other biomarker status][87] reference datasets were used for different types analysis shown in the figures.

PDE7A protein expression in breast cancer samples from the Human Protein Tissue Atlas

Protein expression of PDE7A in normal breast tissues and patient-derived breast cancer samples were retrieved and analyzed from the Human Protein Tissue Atlas (https://www.proteinatlas.org). PDE7A staining quantity in normal breast tissues and patient-derived breast cancer samples for PDE7A protein expression (from the Human Protein Tissue Atlas), and the representative image are shown the corresponding figure.

Bioinformatics analysis of transcription factor binding on PDE7A promoter and correlation analysis

The 1 kb promoter sequence for PDE7A was downloaded from the University of California, Santa Cruz (UCSC) genome browser (https://genome.ucsc.edu/cgi-bin/hgGateway). The promoter sequence was analyzed for identifying transcription factors regulating PDE7A expression using PROMO 3.0 (https://alggen.lsi.upc.es/cgi-bin/promo_v3/promo/promoinit.cgi?dirDB=TF_8.3) software using version 8.3 of TRANSFAC. Transcription factors predicted within a dissimilarity margin of 0% were shortlisted for further analysis. For identifying the correlation between PDE7A and the potential transcription factor that regulates the expression of PDE7A, we used the TCGA data and performed pairwise correlation analysis. We report the Pearson correlation coefficient (R) and p-values for reach pairwise comparison in the relevant figures.

Bioinformatics analysis of E2F1 binding on DHODH promoter

The 1 kb promoter sequence for DHODH was downloaded from the University of California, Santa Cruz (UCSC) genome browser (https://genome.ucsc.edu/cgi-bin/hgGateway). The promoter sequence was analyzed for identifying E2F1 binding site DHDOH expression using rVista2.0 (https://rvista.dcode.org).

Cleavage under targets & release using nuclease (CUT-&-RUN) assay

CUT-&-RUN assays were performed in TNBC cell lines under conditions indicated in respective figure legends using the CUT-&-RUN Assay Kit (Cat #86652; Cell Signaling Technology, Danvers, MA, USA), according to the manufacturer's instructions. Briefly, 5×10^5 cells were harvested, washed, bound to activated Concanavalin A-coated Beads, and permeabilized. Bead–cell complexes were incubated with the respective antibody overnight at 4°C. Cells were then washed three times, resuspended in 100 μL pAG/MNase (Protein A and Protein G fused to micrococcal nuclease), and incubated for 1 h at room temperature. Samples were washed three times with Digitonin Buffer plus Protease Inhibitor Cocktail, resuspended in 150 μL Digitonin Buffer, and incubated for 5 min on ice. MNase was activated by adding calcium chloride, and samples were incubated at 4°C for 30 min. The reaction was stopped by adding 150 μL of 1X Stop Buffer, and samples were incubated at 37°C for 10 min to release the DNA fragments. The DNA was extracted using the included DNA Purification Spin Columns, and qPCR was performed using transcription factor-specific primers for *PDE7A* promoter or *DHODH* promoter. Relative fold-change was calculated as the ratio of immunoprecipitated DNA to IgG-precipitated DNA. Primer sequences and antibodies used for CUT-&-RUN assays are listed in key resources table.

RNA sequencing and data analysis

RNA-sequencing was carried out for 6 samples comprising two groups (DMSO treated and BRL-50481 treated) (Table S2). Single end 76p reads were sequenced utilizing the Illumina NextSeq500 sequencing instrument. Pre-alignment quality assessments of the raw fastq sequences were carried out using FastQC (version 0.11.7) (Andrews, 2010). The number of reads for the 6 samples range from 40M to 44M. The raw fastq sequences were aligned to the human hg38 reference genome (GenBank assembly accession: GCA_000001405.28). The alignments were carried out using STAR (version 2.7.1a) (Dobin et al. 2013) with default parameters. Post-alignment quality assessments were carried out with RSeQC (version 2.6.3) (Wang et al. 2012) and MultiQC (version 1.4) (Ewels et al. 2016). Samtools (version 0.0.19) (Li et al. 2009) and IGV (version 2.6.2) (Thorvaldsdóttir et al. 2013) were used for indexing and viewing the alignments respectively. Gene expression was quantified as gene level using the htseq-count function (version 0.12.3) (Anders et al. 2015). The UCSC gene annotations for the human genome were used. The htseq-count default parameters were used, except for the strand parameter which was set to reverse to consider the strandedness of library. Genes for which there are less than 3 samples with normalized counts greater than or equal to 4 were filtered out. Differentially expressed genes were identified using DESeq2 (version 1.28) (Love et al. 2014). DESeq2 was run with default parameters (Love et al. 2014). Genes were considered differentially expressed if the padj-value <0.05 and the absolute log2 Fold Change >1. The normalized gene expression data was used for downstream analyses such as the heatmaps. The complex heatmap package version 1.12.0 (Gu et al., 2016) was used to generate

heatmaps. The volcano plot was generated with an R Bioconductor package EnhancedVolcano (Blighe K, 2019). To determine the functions altered under conditions of treatment common to two cell lines, over-representation enrichment analysis was performed using the WEB-based GEne SeT AnaLysis Toolkit (Webgestalt) (Liao et al. 2019): the functional database selected was the Gene Ontology Biological Process; the reference set selected was the genome; a list of gene symbols for genes of interest were supplied to Webgestalt; the organism of interest selected was human. The hypergeometric test was used for over-representation analysis for the lists of common differentially expressed genes. The Benjamini and Hochberg method was used to calculate the adjusted *p*-values (q) and the significance cutoff filter was set to $q < 0.05$.

Immunoblot analysis

Whole-cell protein extracts were prepared using IP Lysis Buffer (Pierce Chemical, Rockford, IL, USA) containing Protease Inhibitor Cocktail (Roche, Basel, Switzerland) and Phosphatase Inhibitor Cocktail (Sigma-Aldrich, St. Louis, MO, USA). Lysed samples were centrifuged at 12,000 rpm for 40 min, and clarified supernatants were stored at −80°C. Protein concentrations were determined using Bradford Protein Assay Reagent (Bio-Rad Laboratories, Hercules, CA, USA). Equal amounts of protein samples were electrophoresed on 10% or 12% sodium dodecyl sulfate (SDS)-polyacrylamide gels and transferred onto polyvinylidene difluoride membranes (Millipore, Burlington, MA, USA) using a wet-transfer apparatus (Bio-Rad). Membranes were blocked in 5% skim milk prepared in Tris-buffered saline containing 0.1% Tween 20 and probed with primary antibodies. After washing, the membranes were incubated with the appropriate horseradish peroxidase-conjugated secondary antibodies (1:2,000) (GE Healthcare Life Sciences, Chicago, IL, USA). The blots were developed using SuperSignal West Pico or Femto Chemiluminescent Substrate (Thermo Fisher Scientific, Waltham, MA, USA). All antibodies used for immunoblotting are listed in key resources table.

Immunohistochemistry (IHC)

Formalin-fixed, paraffin-embedded tissue microarray (TMA) slides containing TNBC samples (n = 21) and adjacent normal breast tissues (n = 10) were obtained from US Biomax (#BC081120f). Briefly, following slide deparaffinization, antigen retrieval was performed in citrate buffer (pH 6.0) at 97°C for 20 min, using the Lab Vision PT Module (ThermoFisher Scientific). Endogenous peroxides were blocked by incubation in hydrogen peroxide for 30 min, followed by washing with 1× Tris-buffered saline, and proteins were blocked by incubation with 0.3% BSA for 30 min. Slides were incubated in anti-PDE7A antibody (dilution 1:100) followed by secondary anti-rabbit HRP-conjugated antibody (Dako, Jena, Germany). Slides were then stained using the Dako Liquid DAB+ Substrate Chromogen System and counterstained with Dako Automation Hematoxylin Histological Staining Reagent. PDE7A staining was scored by Dr. Kamaljeet Singh, who was blinded to the identity of each slide. All antibodies used for immunohistochemistry analyses are listed in key resources table.

PDE7A mRNA stability assay

To analyze the stability of *PDE7A* mRNA, TNBC cells were treated with either DMSO or the PI3K inhibitor buparlisib (200 nM) for 48 h followed by treatment with actinomycin D (5 μg/mL) for 0, 1, 2, 3, 4, 6, and 8 h. Total RNA was extracted at the indicated time points using TRIzol (Invitrogen, MA, USA) and purified using RNeasy Mini Columns (Qiagen, MD, USA) according to the manufacturer's instructions. The cDNA was generated using the ProtoScript first-strand cDNA synthesis kit (New England Biolabs, MA, USA) and then qPCR was performed using the Power SYBR Green (Master Mix) (Life Technologies, CA, USA). The half-life of *PDE7A* mRNA was calculated by transforming the mRNA data and calculating slope using simple linear regression (best-fit) using Graphpad Prism. The slope from simple linear regression (best-fit) was finally used to calculate the *PDE7A* mRNA half-life using the formula: $\mathrm{Log}_{10}(0.5)/\mathrm{slope}$.

PDE7A protein degradation assay using MG132

To evaluate the degradation of PDE7A protein upon PI3K inhibition, TNBC cell lines were treated with either DMSO or the PI3K inhibitor buparlisib (200 nM) for 48 h followed by treatment with MG132 (5 μM) for additional 16 h. Thereafter, whole-cell protein extracts were prepared using IP Lysis Buffer (Pierce Chemical, Rockford, IL, USA) containing Protease Inhibitor Cocktail (Roche, Basel, Switzerland) and Phosphatase Inhibitor Cocktail (Sigma-Aldrich, St. Louis, MO, USA). The samples were analyzed for PDE7A protein by immunoblot analysis.

RNA pol II CTD phospho Ser2 antibody ChIP on PDE7A promoter

TNBC cell lines were treated with DMSO or PI3K inhibitor buparlisib (200 nM) for 48 h and enrichment of RNA pol II CTD phospho Ser2 antibody was analyzed using ChIP experiment. ChIP experiment was performed as described previously.[88] Cell lysates were incubated with specific antibodies (anti-IgG (control) or RNA pol II CTD phospho Ser2 antibody) as required and recommended by Active Motif (antibody information has been provided in key resources table). The chromatin was eluted, and DNA was extracted using the included DNA purification columns. qPCR was then performed using *PDE7A* gene specific primers, and relative fold change was calculated as the ratio of immunoprecipitated DNA to IgG-precipitated DNA.

Global metabolomic analysis

For metabolomics analysis, MDA-MB-231 cells were treated with either BRL-50481 (50 μM) and control DMSO for 72 h. 2×10^7 cells were harvested and were analyzed for metabolic pathway alterations using the capillary electrophoresis time-of-flight mass

spectrometry-based basic scan profiling method of Human Metabolome Technologies (Boston, MA, USA). Cells (1×10^6) for each condition in triplicate were analyzed by this method, and samples were prepared as per the recommendations of Human Metabolome Technologies (Boston, MA, USA). For data analysis, peaks detected in capillary electrophoresis time-of-flight mass spectrometry analysis were extracted using automated integration software (MasterHands version 2.16.0.15 developed at Keio University, Tokyo, Japan) to obtain mass/charge ratio (*m/z*), migration time and peak area. Peak area was then converted to relative peak area using the following equation: relative peak area = metabolite peak area/internal standard peak area × number of cells. The peak detection limit was determined based on signal-to-noise ratio = 3. Putative metabolites were then assigned from the Human Metabolomic Technologies standard library and known–unknown peak library on the basis of *m/z* and migration time. All metabolite concentrations were calculated by normalizing the peak area of each metabolite with respect to the area of the internal standard and by using standard curves, which were obtained by single-point (100 μM) calibrations. The profile of peaks of putative metabolites was represented on metabolic pathway maps using Visualization and Analysis of Networks containing Experimental Data (VANTED) software (http://vanted.ipk-gatersleben.de/).

Analysis of key metabolites of pyrimidine biosynthesis pathway

MDA-MB-231 cells expressing an empty vector, wild-type (WT) *DHODH* ORF or catalytic mutant (R135C) *DHODH* ORF were treated with either BRL-50481 (50 μM) and control DMSO for 72 h. Next, 10×10^6 cells were washed twice with ice-cold mannitol (5% in LC-MS grade water) followed by complete removal of the mannitol. Further, cells were scraped, and the cell pellets were snap frozen in liquid nitrogen. Each cell pellet sample was lysed in 250 μL of 80% methanol using an MM 400 mill mixer at 30 Hz for 2 min, followed by centrifugation. The clear supernatants were diluted with a 13C10-GTP internal standard solution. The protein pellets were collected and used for protein assay using a standardized BCA procedure. The calibration solutions containing 13C10-GTP were prepared with standard substances of the targeted metabolites of pyrimidine biosynthesis pathway in 16% methanol. 10-μL aliquots of the resultant sample and calibration standard solutions were injected in turn into a C18 UPLC column to run MRM/MS with (−) ion detection on a Waters Acquity UPLC system, coupled to a Sciex QTRAP 6500 Plus MS instrument. The binary solvent mobile phase for gradient elution consisted of a tributylamine solution and acetonitrile. LC-MRM/MS was carried out under optimized separation and detection conditions. For quantification of the metabolites, linear regression calibration curves of the individual metabolites were constructed with the data acquired from the injected calibration solutions. The concentrations of the detected compounds in the samples were calculated by interpolating the calibration curves with data from the injected sample solutions.

QUANTIFICATION AND STATISTICAL ANALYSIS

All experiments were conducted with at least three biological replicates. The number of replicates or number of mice are indicated in the corresponding figures and/or figure legends. Results for individual experiments are expressed as the mean ± standard error of the mean (SEM). Error bars represent SEM. Statistical analysis of tumor progression from TNBC cell lines and TNBC PDX that were subcutaneous injected in mice was performed using the area under the curve (AUC) method in the GraphPad Prism software, version 10.0, for Macintosh (GraphPad Software; https://www.graphpad.com). For IHC experiments, contingency analysis using the chi-square test was used to determine the *p*-values. For the remaining experiments, *p*-values were calculated using two-tailed unpaired Student's *t* tests in the GraphPad Prism software, version 10.0, for Macintosh. A p-value <0.05 was considered statistically significant.

Cell Reports Medicine, Volume *6*

Supplemental information

PDE7A inhibition suppresses triple-negative breast cancer by attenuating *de novo* pyrimidine biosynthesis

Parmanand Malvi, Suresh Bugide, Roshan Dutta, Kiran Kumar Reddi, Yvonne J.K. Edwards, Kamaljeet Singh, Romi Gupta, and Narendra Wajapeyee

SUPPLEMENTARY FIGURES and LEGENDS

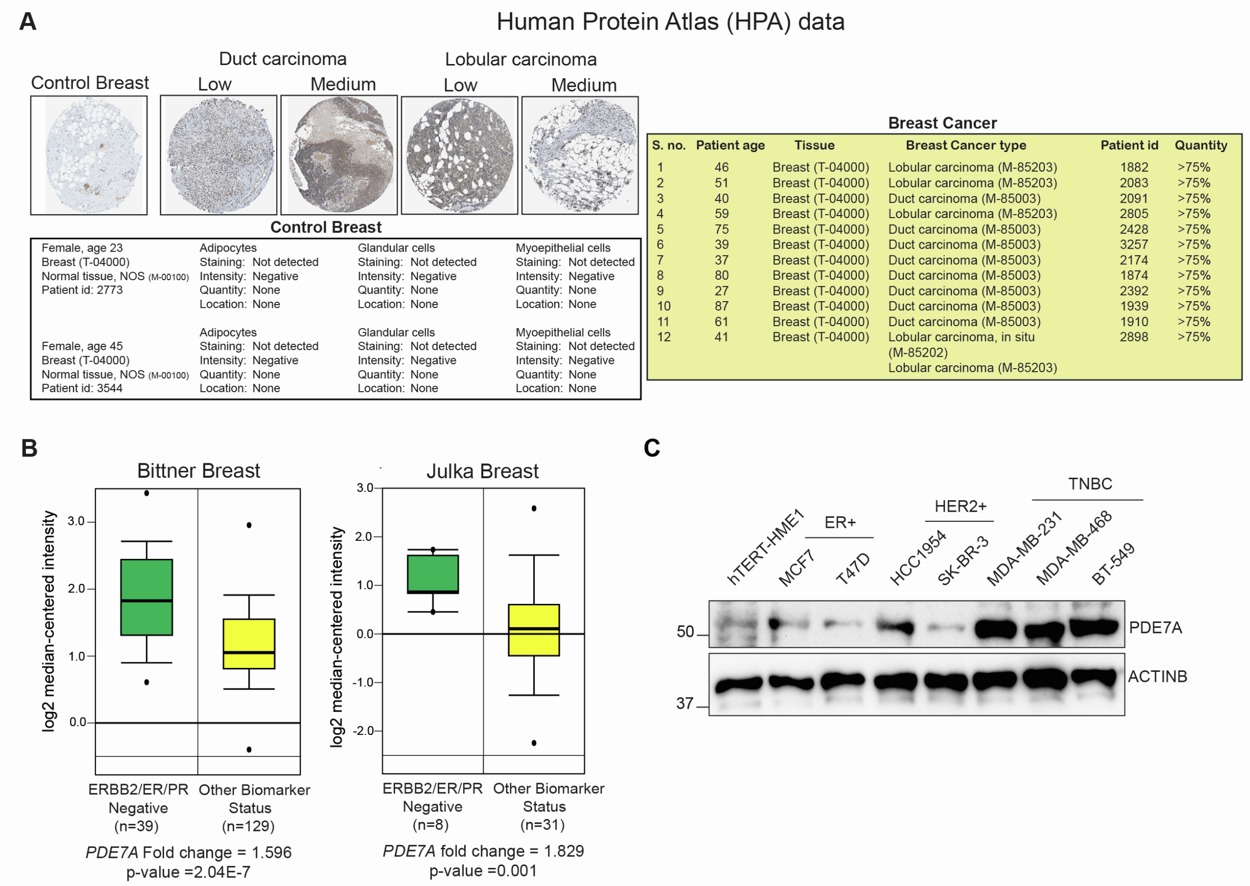

Figure S1. PDE7A is overexpressed in TNBC. Related to Figure 1. A. Relative expression of PDE7A protein in patient-derived breast cancer samples. Representative image of PDE7A protein expression in normal breast tissue and breast cancer samples from the Human Protein Tissue Atlas. **B**. *PDE7A* mRNA expression levels in TNBC samples compared with other breast cancer subtypes (ERBB2+, ER+ and ERBB2/ER+) in the indicated datasets. Fold changes and p-values for the indicated databases under indicated conditions are shown. **C.** Immunoblot analysis of PDE7A expression in non-transformed human mammary epithelial cells (hTERT-HME1) and in the breast cancer cell lines of the indicated subtypes. ACTINB was used as a loading control.

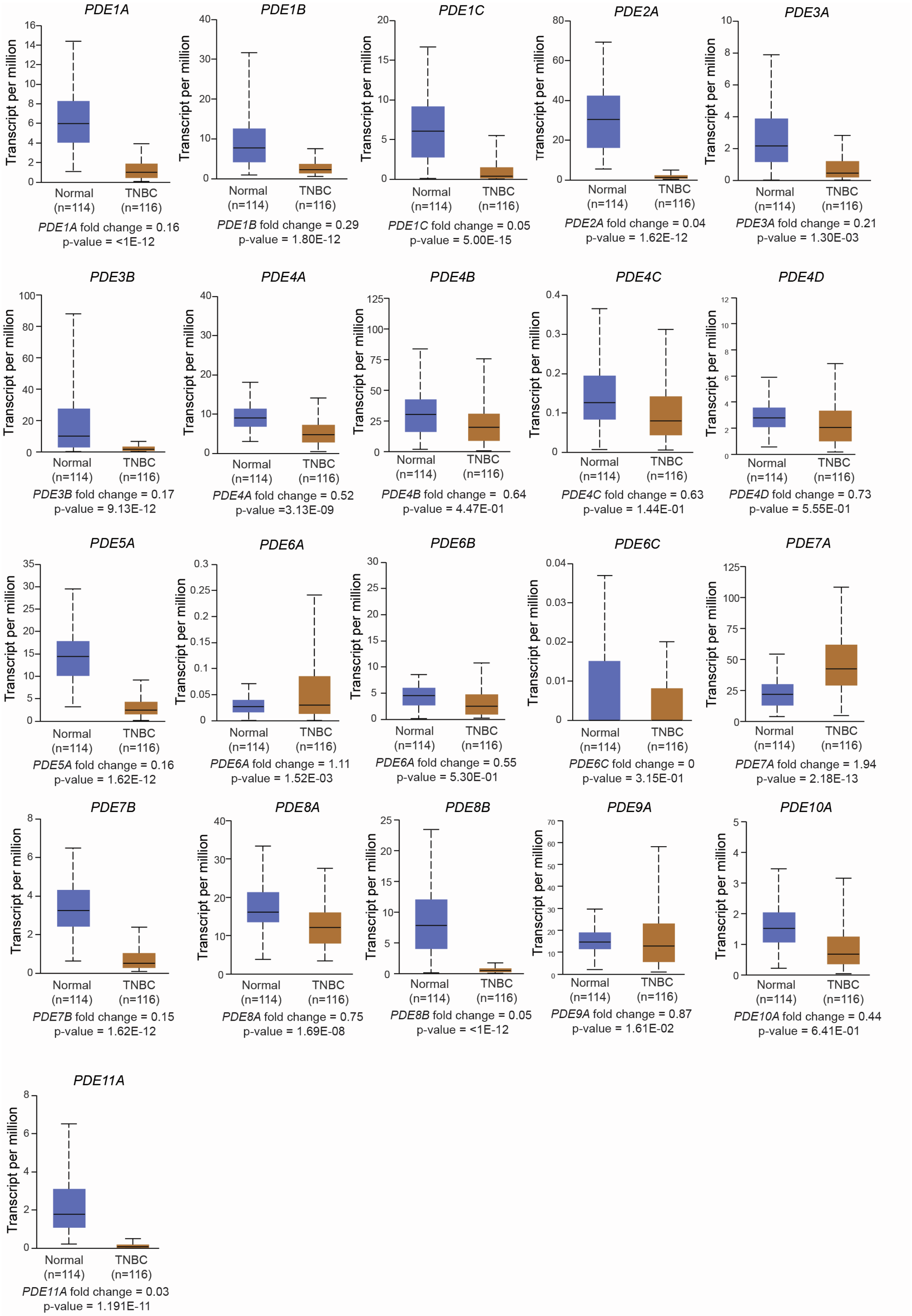

PDE1A
Transcript per million
Normal (n=114)
TNBC (n=116)
PDE1A fold change = 0.16
p-value = <1E-12
PDE1B
PDE1B fold change = 0.29
p-value = 1.80E-12
PDE1C
PDE1C fold change = 0.05
p-value = 5.00E-15
PDE2A
PDE2A fold change = 0.04
p-value = 1.62E-12
PDE3A
PDE3A fold change = 0.21
p-value = 1.30E-03
PDE3B
PDE3B fold change = 0.17
p-value = 9.13E-12
PDE4A
PDE4A fold change = 0.52
p-value =3.13E-09
PDE4B
PDE4B fold change = 0.64
p-value = 4.47E-01
PDE4C
PDE4C fold change = 0.63
p-value = 1.44E-01
PDE4D
PDE4D fold change = 0.73
p-value = 5.55E-01
PDE5A
PDE5A fold change = 0.16
p-value = 1.62E-12
PDE6A
PDE6A fold change = 1.11
p-value = 1.52E-03
PDE6B
PDE6A fold change = 0.55
p-value = 5.30E-01
PDE6C
PDE6C fold change = 0
p-value = 3.15E-01
PDE7A
PDE7A fold change = 1.94
p-value = 2.18E-13
PDE7B
PDE7B fold change = 0.15
p-value = 1.62E-12
PDE8A
PDE8A fold change = 0.75
p-value = 1.69E-08
PDE8B
PDE8B fold change = 0.05
p-value = <1E-12
PDE9A
PDE9A fold change = 0.87
p-value = 1.61E-02
PDE10A
PDE10A fold change = 0.44
p-value = 6.41E-01
PDE11A
PDE11A fold change = 0.03
p-value = 1.191E-11

Figure S2. Expression of phosphodiesterases (PDEs) in TNBC. Related to Figure 1. mRNA expression for all phosphodiesterases (PDEs) in the TNBC samples compared to normal breast tissue samples in the breast cancer TCGA dataset analyzed using UALCAN. Fold changes and p-values for individual PDE mRNAs compared to normal breast tissue samples are shown.

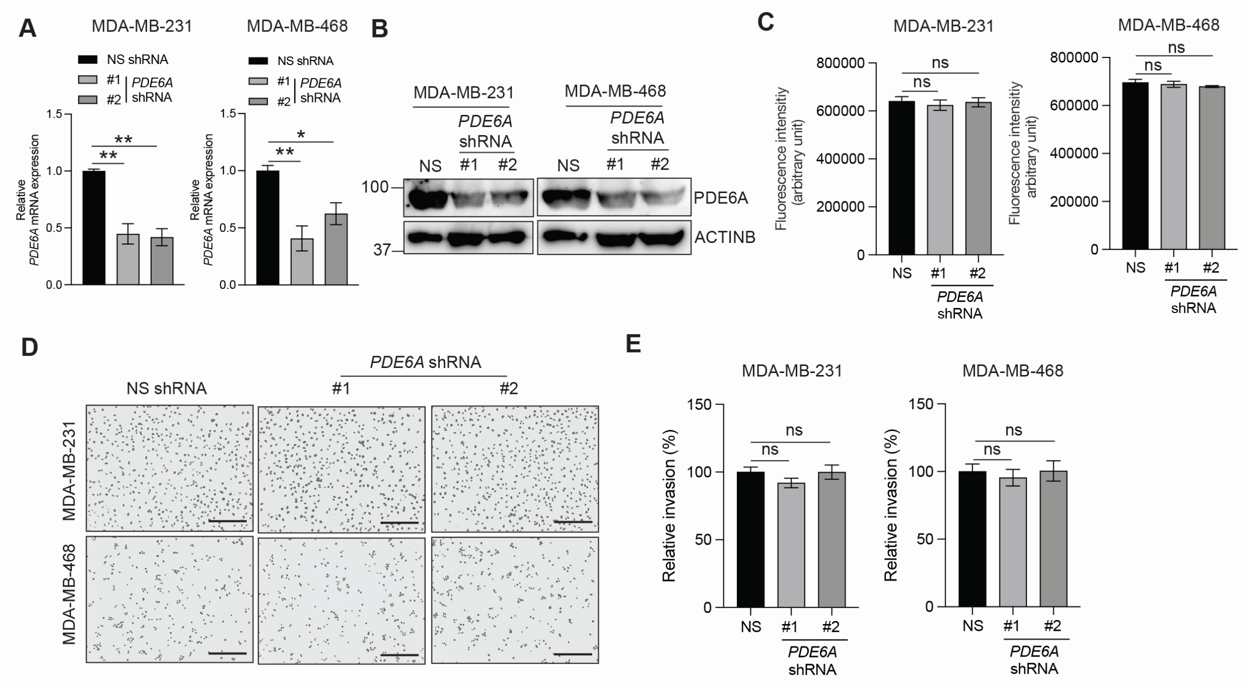

Figure S3. PDE6A knockdown does not suppress TNBC growth and invasion ability. Related to Figure 1. A. Indicated TNBC cell lines expressing either non-specific (NS) shRNA or *PDE6A* shRNAs were analyzed for the expression of *PDE6A* mRNA expression by RT-qPCR (n = 3 biological replicates/group). *PDE6A* mRNA expression is plotted under indicated conditions. *ACTINB* was used as a normalization control. **B.** Indicated TNBC cell lines expressing either NS shRNA or *PDE6A* shRNAs were analyzed for the expression of PDE6A protein by immunoblotting. PDE6A protein expression is shown under indicated conditions. ACTINB was used as a loading control. **C.** Indicated TNBC cell lines expressing NS shRNA or *PDE6A* shRNAs were analyzed by quantitative soft-agar assay using the CytoSelect 96-well quantitative soft agar assay. Fluorescence intensities (arbitrary unit) under the indicated conditions are shown (n = 3 biological replicates/group). **D.** Indicated TNBC cell lines expressing NS shRNA or *PDE6A* *s*hRNAs were analyzed for invasive capacity using a Matrigel-based invasion assay. Representative images are shown. Scale bar, 200 µm. **E.** Relative invasion (%) relative to the NS shRNA expressing cells is plotted for the experiment shown in panel D (n = 3 biological replicates/group). All quantitative data represent the mean ± SEM. ns= not significant p-value, *p < 0.05, **p < 0.01.

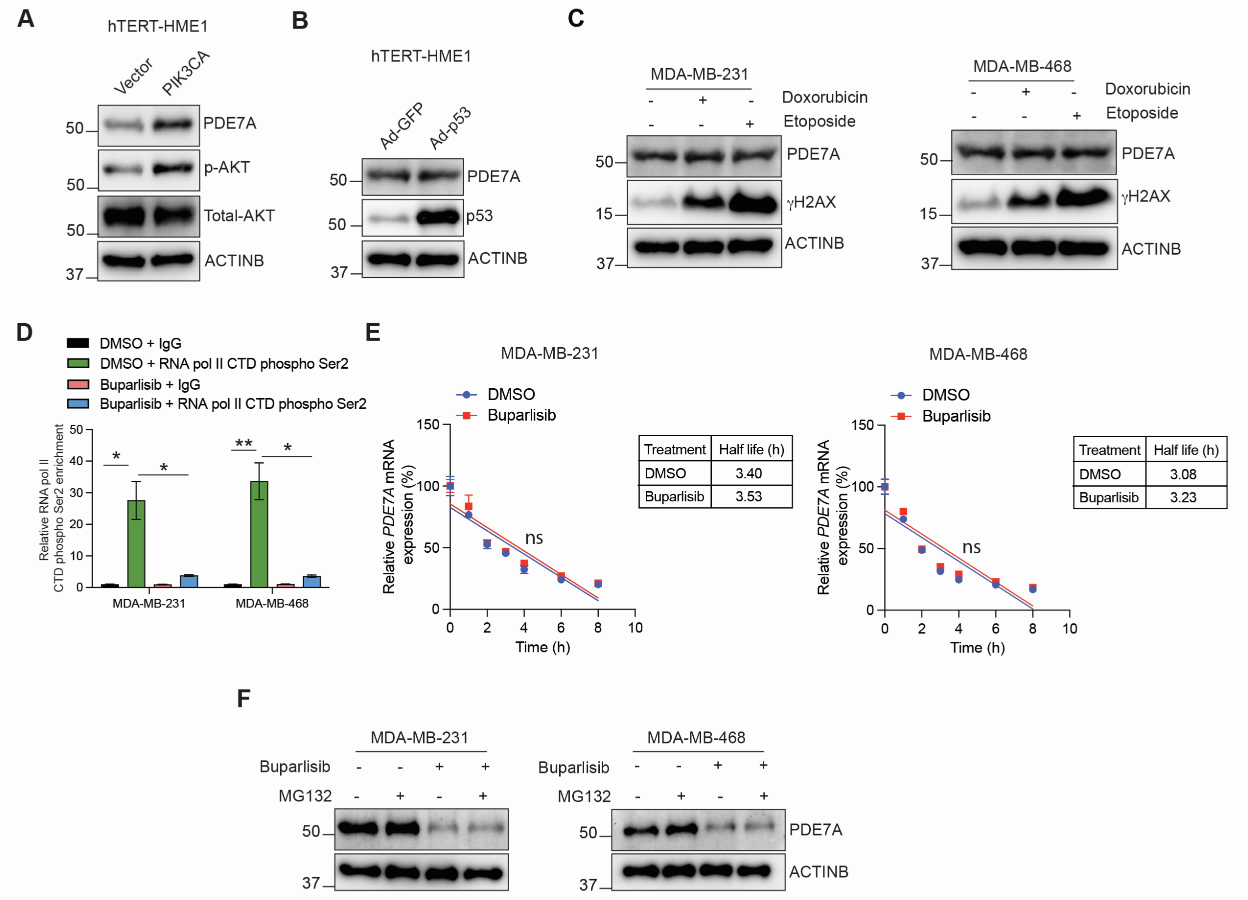

Figure S4. PI3K kinase stimulates PDE7A expression independent of mRNA stability and protein stability in TNBC cells. Related to Figure 2. A. Immunoblot analysis of the expression of the indicated proteins in PIK3CA-expressing hTERT-HME1 cells relative to vector-expressing cells. ACTINB was used as a loading control. **B.** Immunoblot analysis of the expression of the indicated proteins in adenoviral p53 (Ad-p53)-expressing hTERT-HME1 cells relative to vector (Ad-GFP)-expressing cells. ACTINB was used as a loading control. **C.** The indicated TNBC cell lines were treated with DMSO, doxorubicin (1 µM) or etoposide (25 µM) for 24 h. PDE7A protein expression was measured using immunoblot analysis under the indicated conditions. ACTINB was used as a loading control. **D.** Indicated TNBC cell lines were treated with DMSO or PI3K inhibitor buparlisib (200 nM) for 48 h and enrichment of RNA pol II CTD phospho Ser2 was measured relative to control anti-IgG antibody using chromatin immunoprecipitation (ChIP) assay. Enrichment under indicated conditions for RNA pol II CTD phospho Ser2 on PDE7A gene body relative to DMSO treated indicated cells is shown (n = 3 biological replicates/group). **E.** Indicated TNBC cell lines were treated with either DMSO or the PI3K inhibitor buparlisib (200 nM) for 48 h, followed by treatment with actinomycin D (5 µg/mL) for the indicated time points, and analyzed for *PDE7A* mRNA expression by RT-qPCR (n = 3 biological replicates/group). Relative mRNA expression levels for each condition and the half-life is shown. The half-life of *PDE7A* mRNA was calculated using simple linear regression in GraphPad Prism software (version 10.0, for Macintosh) and is shown. **F.** The indicated TNBC cell lines were treated with either DMSO or the

PI3K inhibitor buparlisib (200 nM) for 48 h, followed by treatment with the proteasome inhibitor MG132 (5 μM) for additional 16 h, and were analyzed for PDE7A protein expression by immunoblot analysis. ACTINB was used as a loading control. ACTINB was used as a loading control. All quantitative data represent the mean ± SEM. ns= not significant, *p<0.05, **p<0.01.

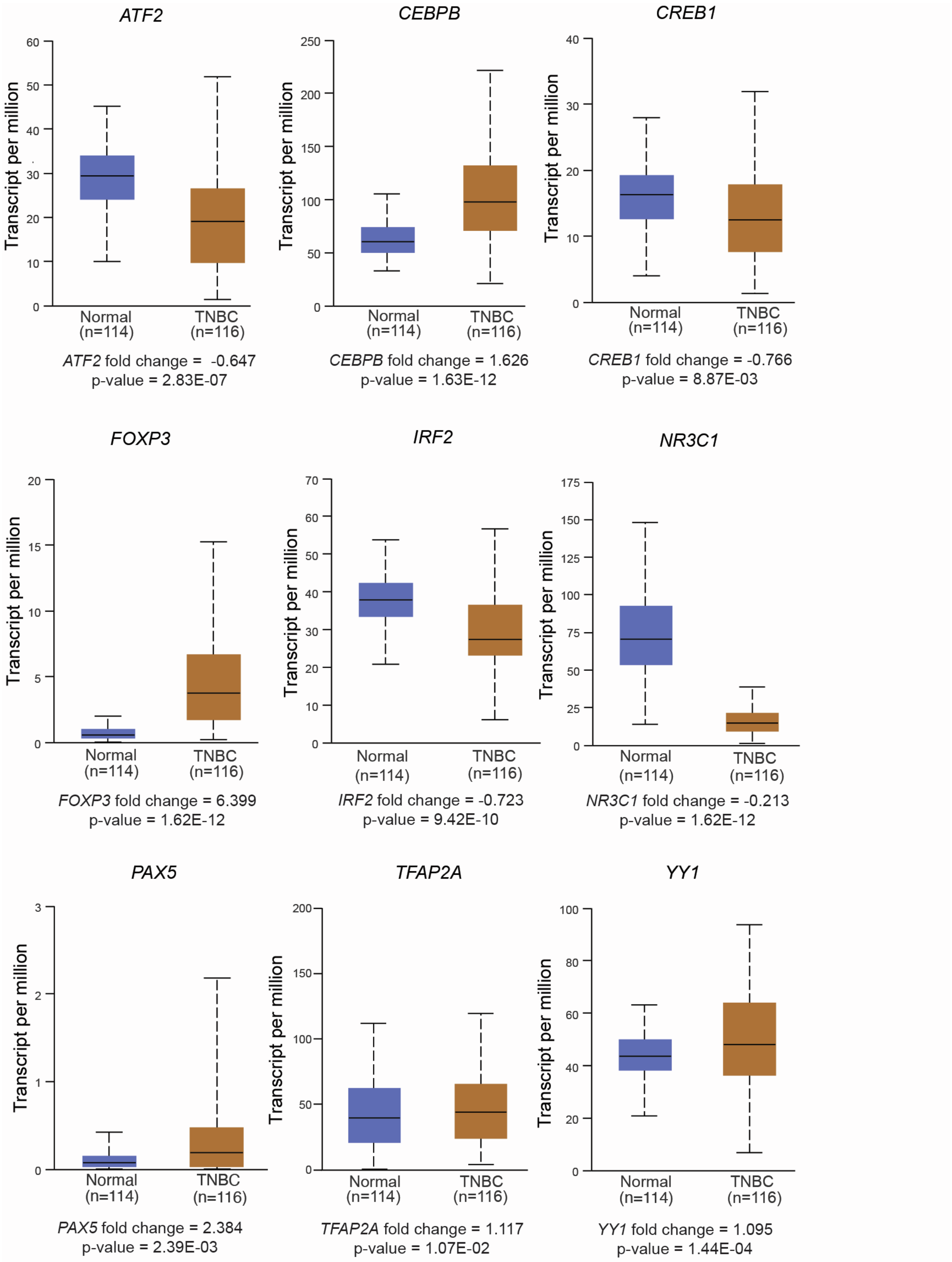

Figure S5. mRNA expression of indicated transcription factors in TNBC. Related to Figure 2. Using UALCAN, the mRNA expression of the indicated transcription factors in TNBC was examined by comparing normal breast tissue samples with those from TNBC in the TCGA dataset. Fold changes and p-values for indicated transcription factors are shown.

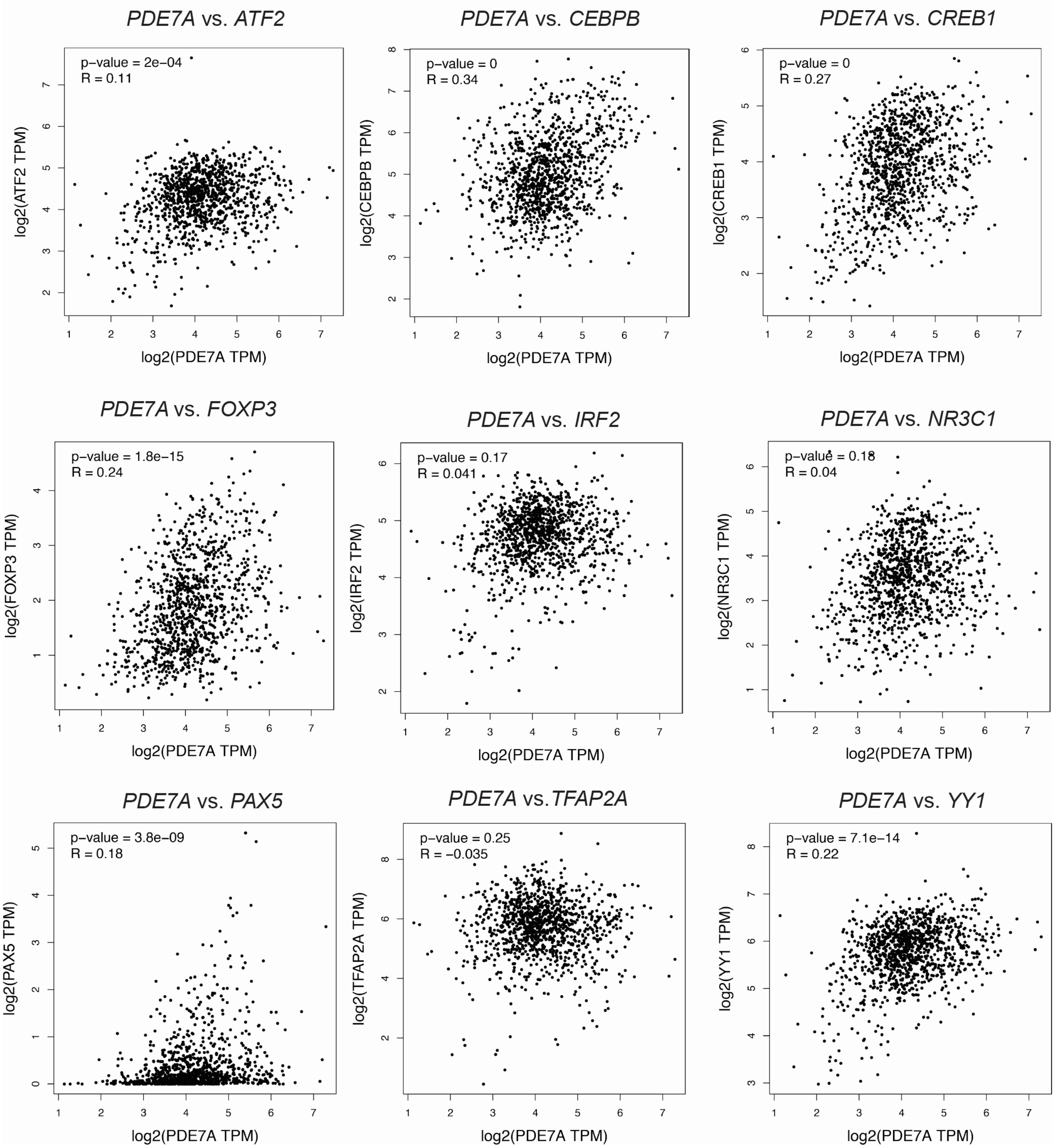

Figure S6. Correlation analysis for indicated transcription factors with a potential role in stimulating *PDE7A* transcription in TNBC. Related to Figure 2. A correlation analysis between the mRNA levels of the indicated transcription factors and *PDE7A* mRNA was performed using GEPIA on the TCGA dataset. Pearson correlation coefficient (R) and p-values for each correlation analysis are shown.

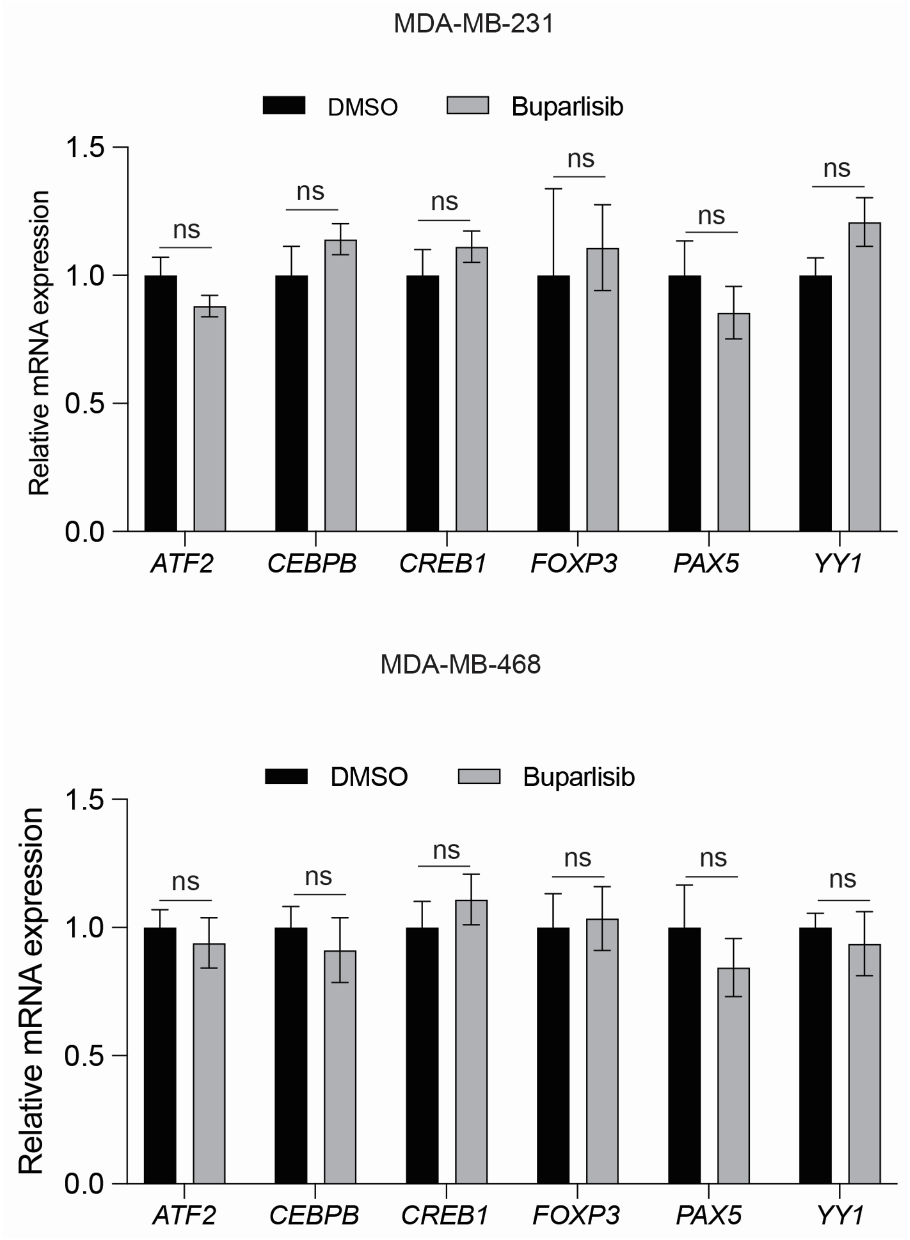

Figure S7. Measurement of indicated transcription factors following PI3K inhibitor buparlisib-treatment. Related to Figure 2. The indicated TNBC cell lines were treated with DMSO or buparlisib (200 nM) for 48 h and analyzed for the expression of indicated mRNA by RT-qPCR. Relative mRNA expression in buparlisib-treated cells relative to DMSO-treated cells is plotted (n = 3 biological replicates/group). *ACTINB* was used as a normalization control. All quantitative data represent the mean ± SEM. ns= not significant p-values.

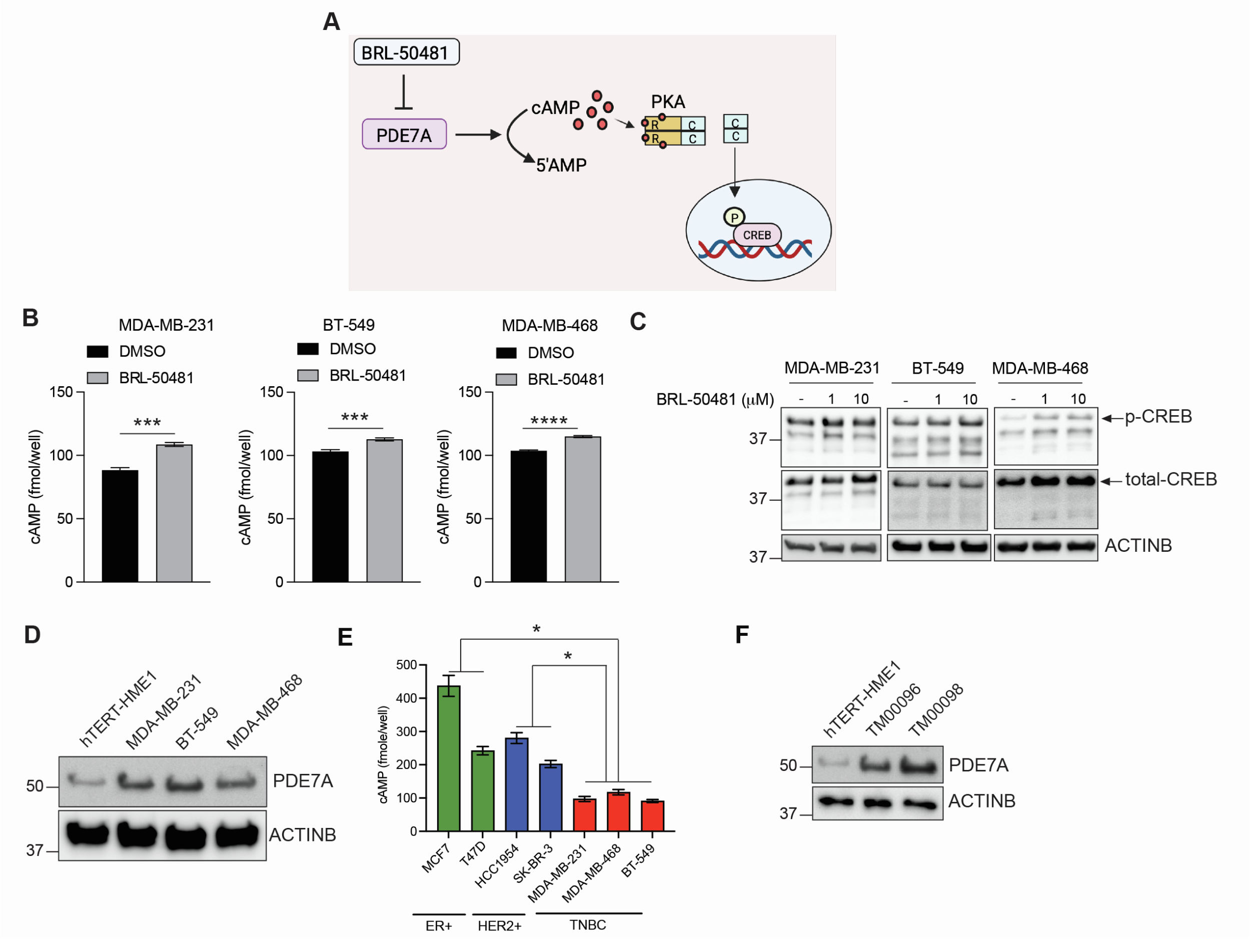

Figure S8. PDE7A is overexpressed in TNBC cells and regulates cAMP and CREB. Related to Figure 3. A. Schematic showing phosphodiesterase 7A (PDE7A) regulation of phosphorylated cyclic adenosine monophosphate (cAMP)-response element–binding protein (p-CREB) and the mechanism through which PDE7A inhibition activates CREB. **B.** The indicated TNBC cell lines were treated with DMSO or BRL-50481 (10 μM) for 72 h, and cAMP levels were measured (n = 3 biological replicates/group). **C.** The indicated TNBC cell lines were treated with DMSO or BRL-50481 at the indicated concentrations for 72 h, and p-CREB and total CREB levels were measured. ACTINB was measured as a loading control. **D.** PDE7A protein expression in the indicated TNBC cell lines compared to the non-transformed human mammary epithelial cells (hTERT-HME1). ACTINB was measured as a loading control. **E.** cAMP levels in the indicated cells lines. cAMP levels were measured in the indicated breast cancer cell lines of indicated breast cancer subtypes (n = 3 biological replicates/group). **F.** PDE7A protein expression in indicated TNBC PDXs compared to the hTERT-HME1 cells. ACTINB was measured as a loading control. All quantitative data represent the mean ± SEM. *p<0.05, ***p<0.001, ****p<0.0001.

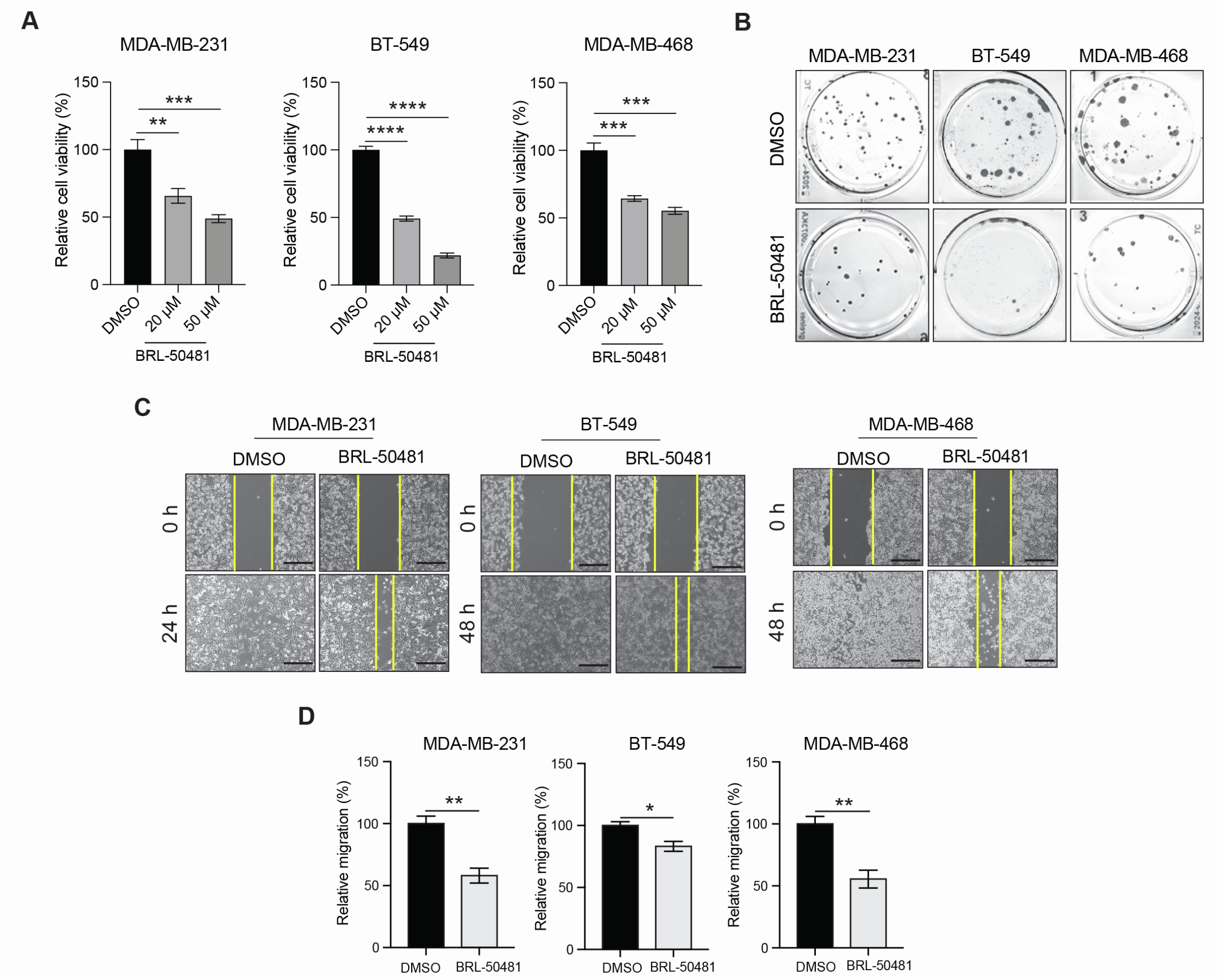

Figure S9. Pharmacological inhibition of PDE7A suppresses growth and migration of TNBC cells. Related to Figure 3. A. The indicated TNBC cell lines were treated with DMSO or BRL-50481 (20 or 50 µM) for 72 h. Cell viability was analyzed using an MTT assay and is presented relative to DMSO-treated cells (n = 4 biological replicates/group). **B.** Representative images of clonogenic assays to measure cell survival for the indicated TNBC cell lines under DMSO or BRL-50481 (50 µM) treatment conditions. **C.** The indicated TNBC cell lines were treated with DMSO or BRL-50481 (50 µM) and analyzed for migration using a wound-healing assay. Representative images are shown. Scale bar, 200 µm. **D.** Relative migration (%) is plotted relative to the DMSO-treated condition for the experiment shown in panel C (n = 3 biological replicates/group). All quantitative data represent the mean ± SEM. **p*<0.05, ***p*<0.01, ****p*<0.001, *****p*<0.0001.

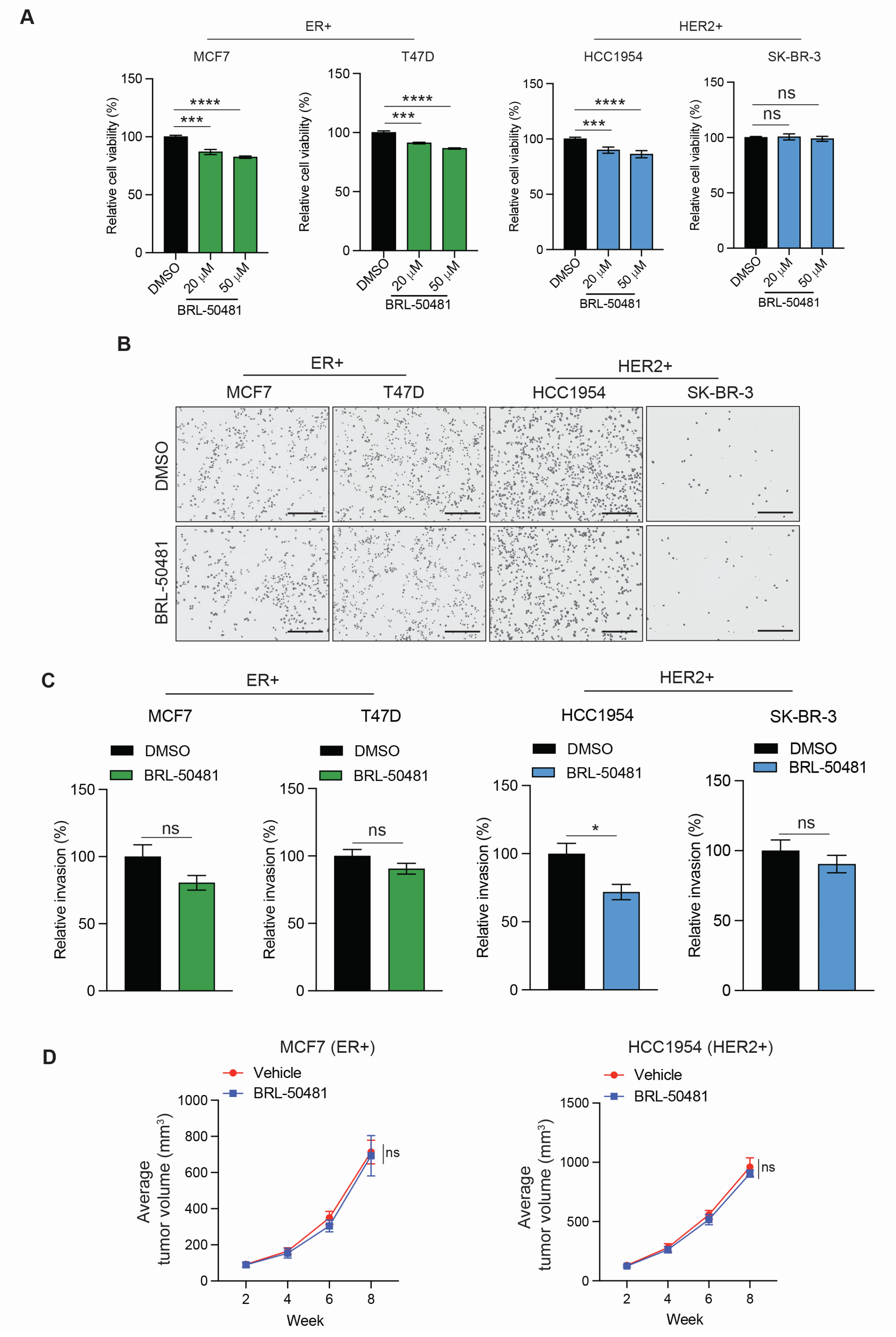
A
ER+
HER2+
MCF7
T47D
HCC1954
SK-BR-3
Relative cell viability (%)
DMSO
20 μM
50 μM
BRL-50481
****
***
ns
B
DMSO
BRL-50481
C
Relative invasion (%)
*
D
MCF7 (ER+)
HCC1954 (HER2+)
Vehicle
Average tumor volume (mm³)
Week

Figure S10. PDE7A inhibition does not impact non-TNBC growth and invasion in cell culture and tumor growth in mice. Related to Figure 3. A. The indicated breast cancer cell lines from the indicated subtypes were treated with DMSO or BRL-50481 (20 or 50 µM) for 72 h. Cell survival was measured using the MTT assay and is presented relative to that of DMSO-treated cells (n = 5 biological replicates/group). **B.** The indicated breast cancer cell lines from the indicated subtypes were treated with DMSO or BRL-50481 (50 µM) for 20 h, and analyzed for invasive capacity using a Matrigel-based invasion assay. Representative images are shown. Scale bar: 200 µm. **C.** Relative invasion (%) compared to the DMSO-treated cells is plotted for the experiment shown in panel B (n = 3 biological replicates/group). **D.** MCF7 and HCC1954 cells were injected subcutaneously into the flanks of female NSG mice (n = 5/group, each cell line). Mice were treated every alternate day with the vehicle or BRL-50481 (25 mg/kg) intraperitoneally and tumor growth was measured. Average tumor volumes at the indicated time points under the indicated conditions are plotted. All quantitative data represent the mean ± SEM. ns= not significant. All quantitative data represent the mean ± SEM. ns= not significant, *p<0.05, ***p<0.001 and ****p<0.0001.

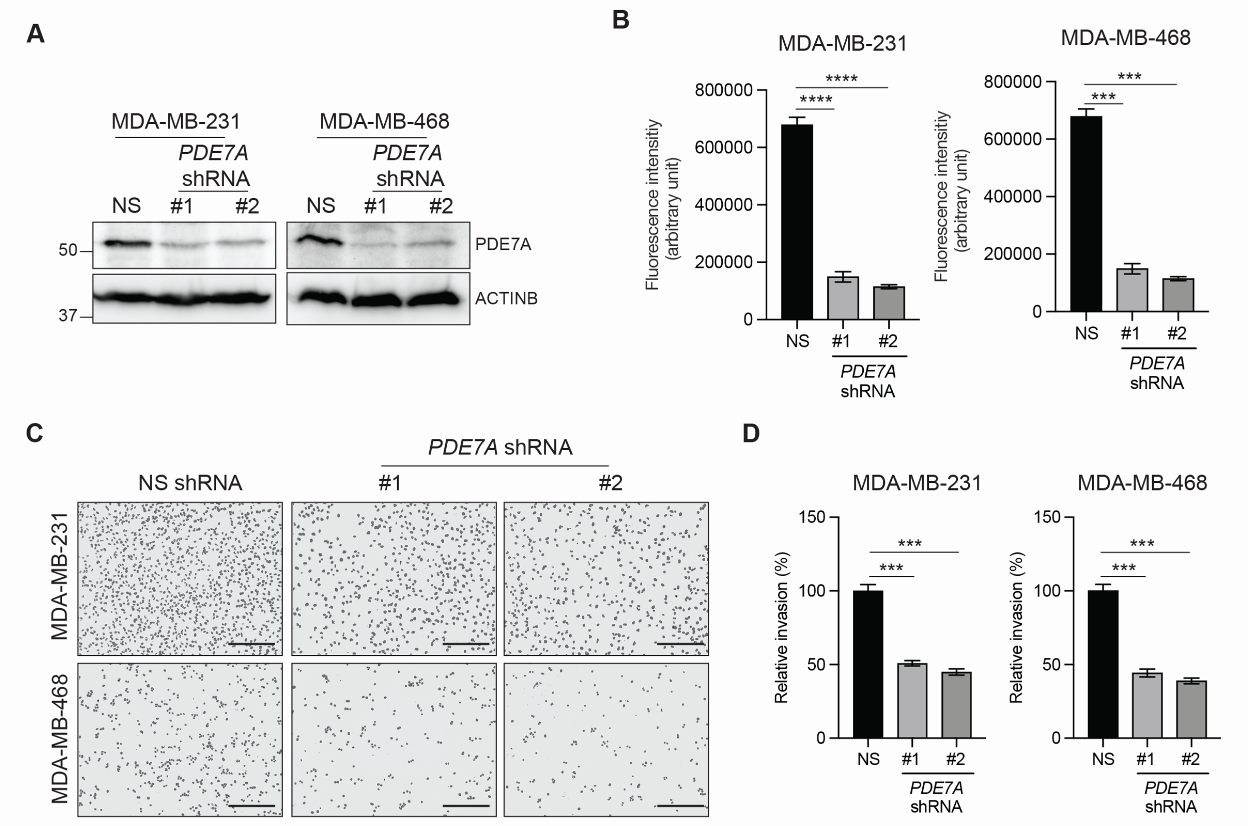

Figure S11. PDE7A knockdown inhibits TNBC growth and invasion. Related to Figure 3. A. The indicated TNBC cell lines expressing either non-specific (NS) shRNA or *PDE7A* shRNAs were analyzed for PDE7A protein expression by immunoblotting. ACTINB was used as a loading control. **B.** The indicated TNBC cell lines expressing either NS shRNA or *PDE7A* shRNAs were analyzed using the CytoSelect 96-well quantitative soft agar assay kit. Fluorescence intensities (arbitrary unit) under the indicated conditions are shown (n = 3 biological replicates/group). **C.** The indicated TNBC cell lines expressing either NS shRNA or *PDE7A* shRNAs were analyzed for invasive capacity using a Matrigel-based invasion assay. Representative images are shown. Scale bar, 200 µm. **D.** Relative invasion (%) relative to the NS shRNA-expressing cells is plotted for the experiment shown in panel C (n = 3 biological replicates/group). All quantitative data represent the mean ± SEM. ***p < 0.001, ****p < 0.0001.

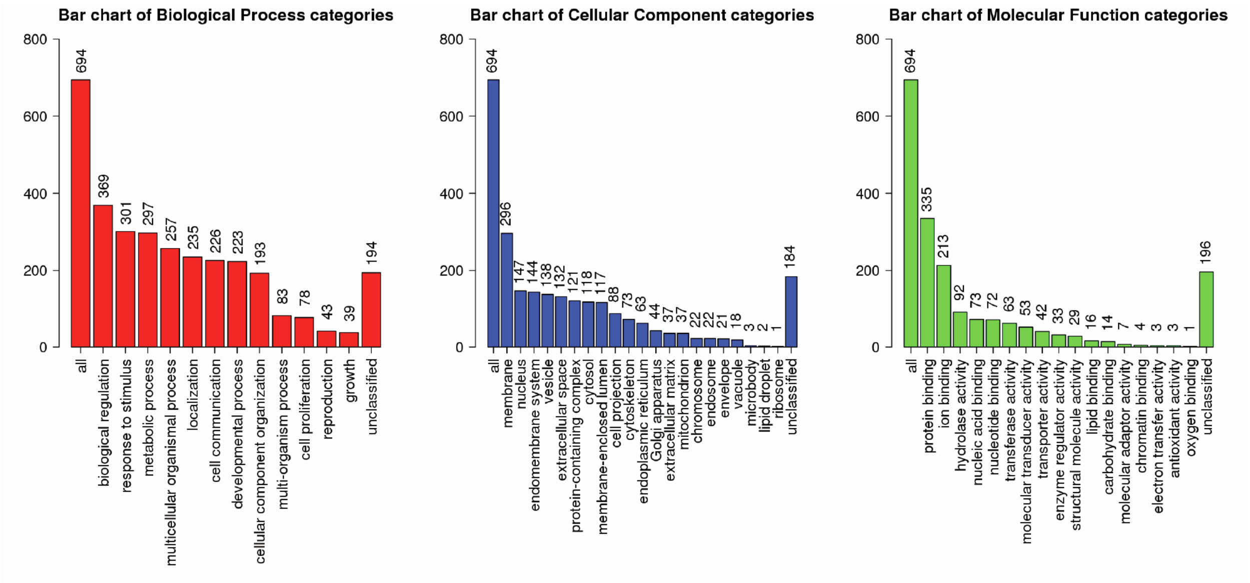

Figure S12. PDE7A inhibition suppresses TNBC cell growth. Related to Figure 4. Biological pathways that were altered in MDA-MB-231 cells treated with the phosphodiesterase 7A (PDE7A) inhibitor BRL-50481 (50 μM) for 72 h compared with DMSO-treated cells based on the mRNA expression profiles identified from RNA-sequencing results.

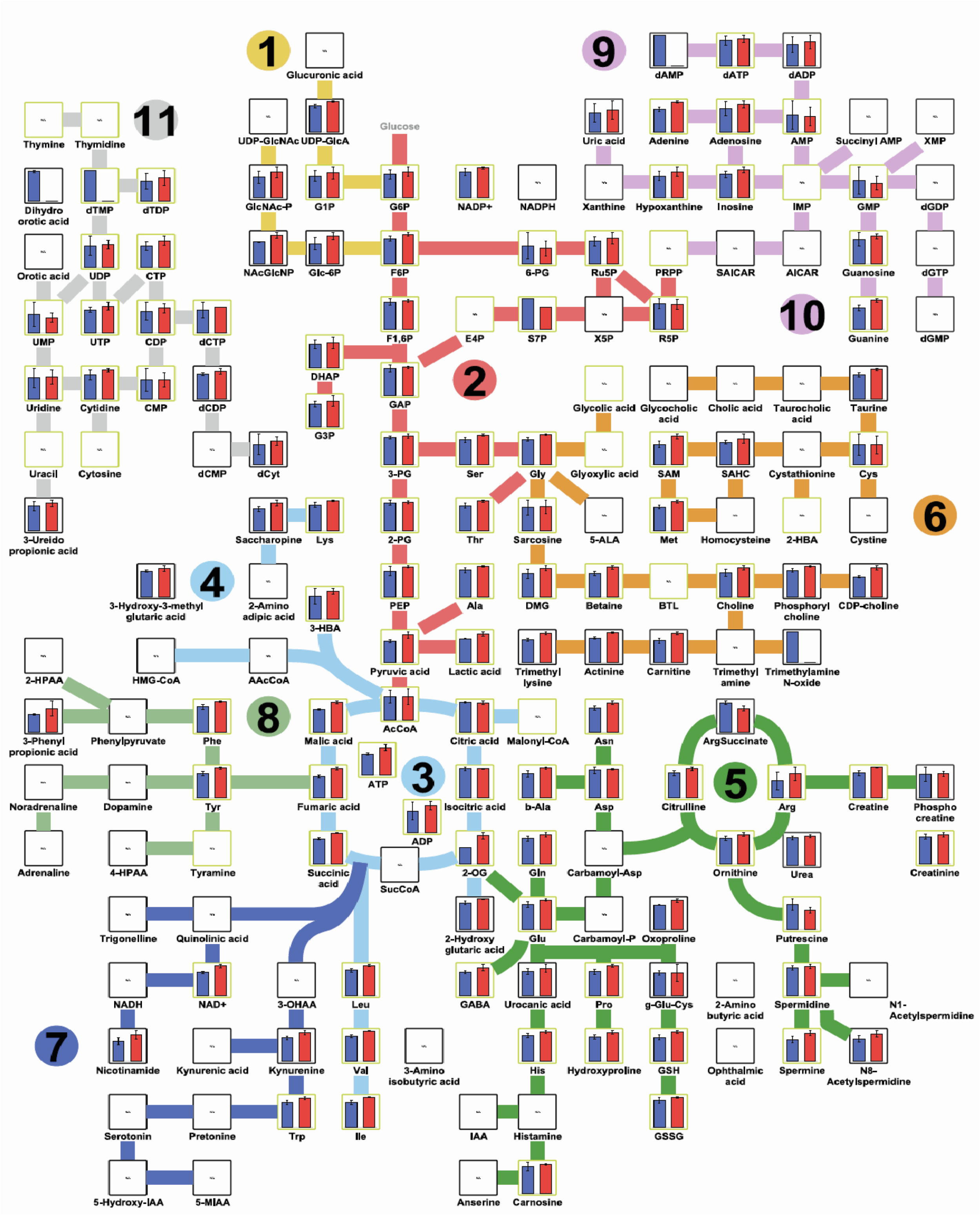

Figure S13. Pharmacological inhibition of PDE7A alters several metabolic pathways and metabolites. Related to Figure 4. Metabolic pathways and metabolites altered in MDA-MB-231 cells treated with the phosphodiesterase 7A (PDE7A) inhibitor BRL-50481 (50 µM) for 72 h

compared with control DMSO-treated cells based on the metabolite profiles from the metabolomics analysis.

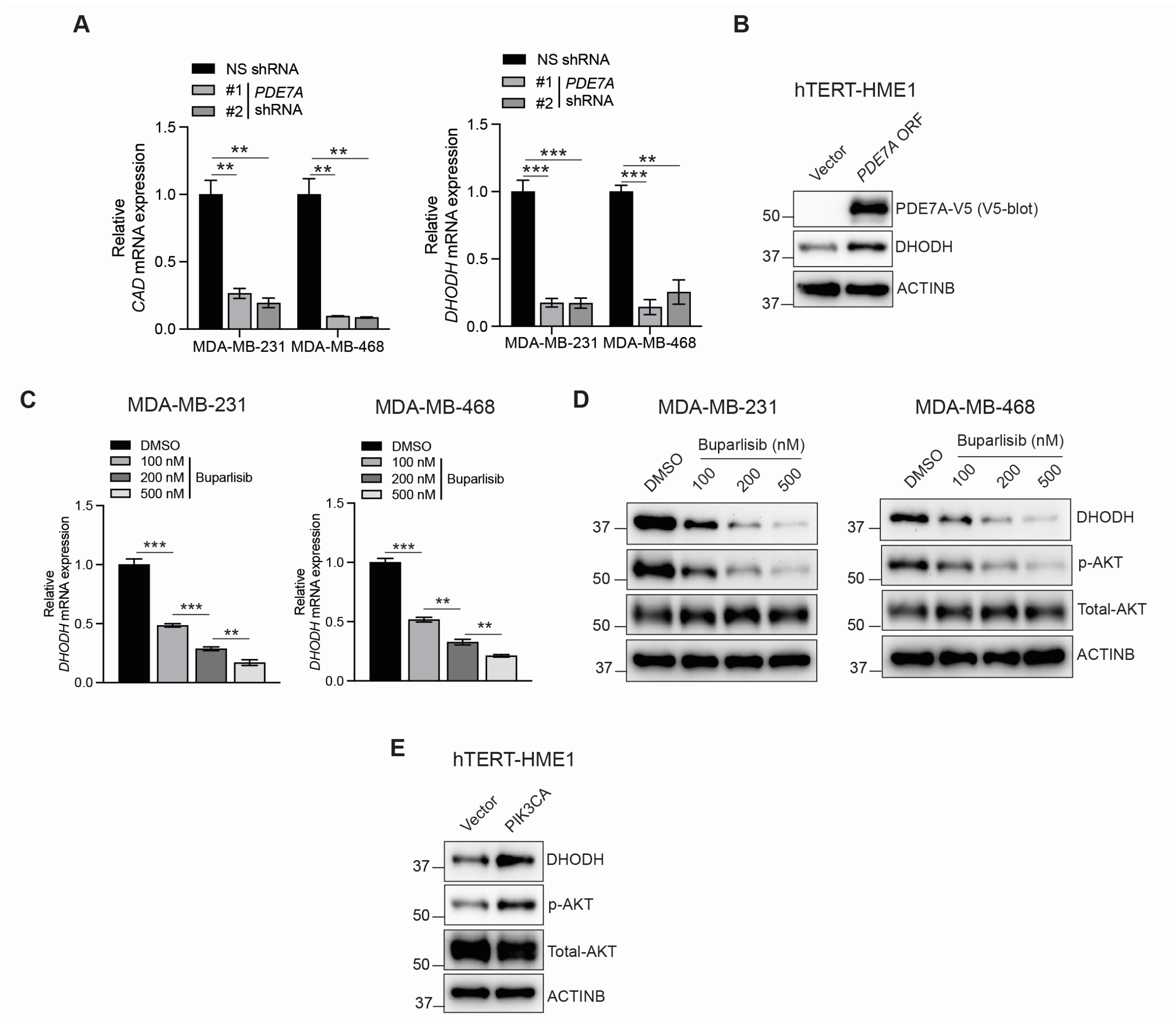

Figure S14. Impact of PDE7A knockdown on de novo pyrimidine biosynthesis genes and role of PI3K pathway in stimulating DHODH expression. Related to Figures 4 and 5. A. The indicated TNBC cell lines expressing non-specific (NS) shRNA or *PDE7A* shRNAs were analyzed for the expression of *DHODH* and *CAD* mRNA using RT-qPCR analysis (n = 3 biological replicates/group). Relative mRNA expression for *DHODH* and *CAD* in the indicated TNBC cell lines under the indicated conditions is shown. **B.** Immunoblot analysis of the expression of the indicated proteins in V5-tagged *PDE7A* ORF-expressing hTERT-HME1 cells under the indicated conditions. ACTINB was used as a loading control. **C.** The indicated TNBC cell lines were treated with either DMSO or the PI3K inhibitor buparlisib at the indicated concentrations for 48 h. *DHODH* mRNA expression was measured using RT-qPCR under the indicated conditions (n = 3 biological replicates/group). *ACTINB* was used as a normalization control. **D.** The indicated TNBC cell lines were treated with either DMSO or the PI3K inhibitor buparlisib at the indicated concentrations for 48 h. DHODH protein expression was measured using immunoblot analysis under the indicated conditions. ACTINB was used as a loading control. **E.** Immunoblot analysis of the expression of the indicated proteins in empty-vector or constitutively active PIK3CA-

expressing hTERT-HME1 cells under the indicated conditions. ACTINB was used as a loading control. All quantitative data represent the mean ± SEM. **p<0.01 and ***p<0.001.

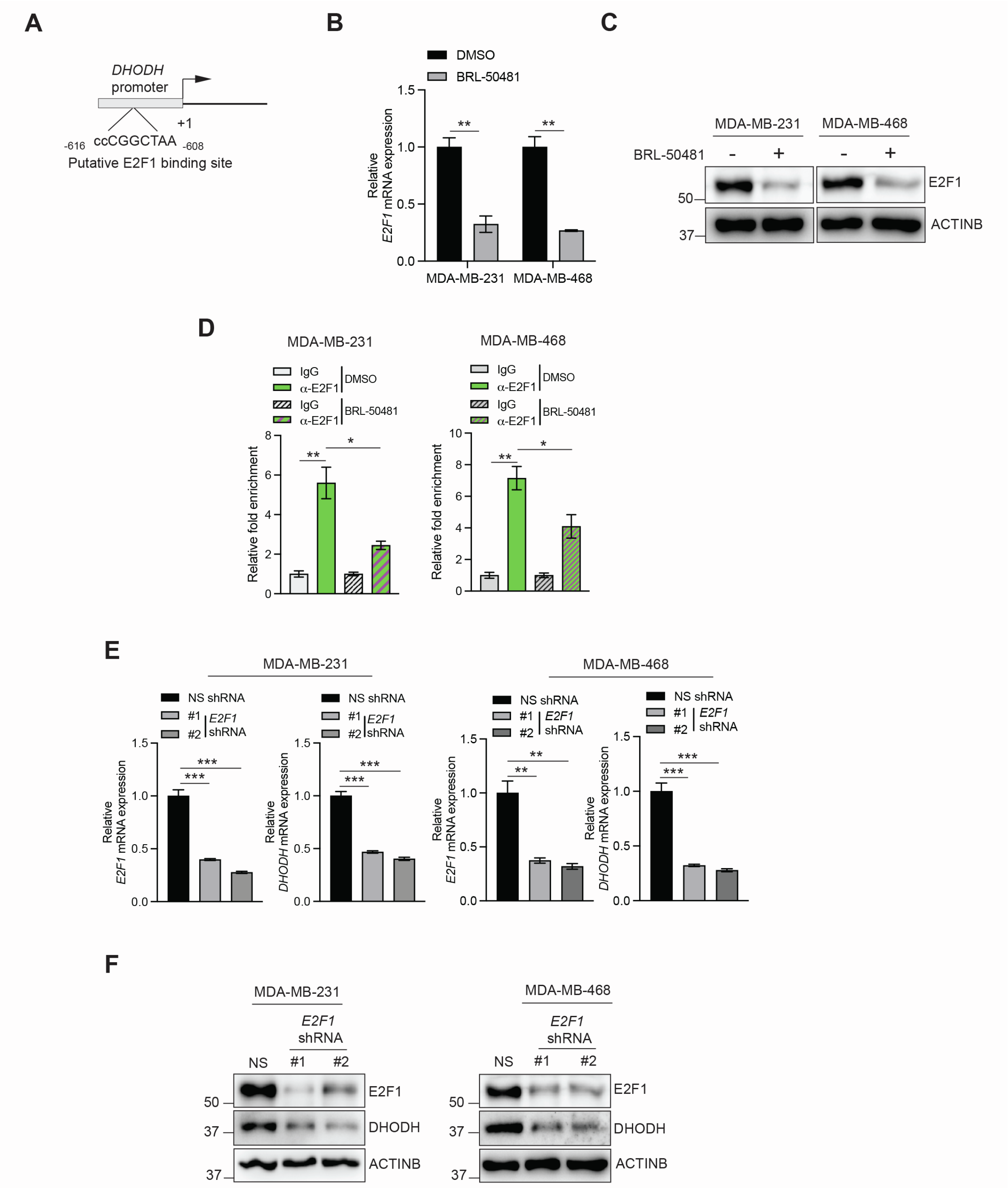

Figure S15. PDE7A via E2F1 stimulates the expression of DHODH in TNBC. Related to Figure 5. A. A schematic showing the E2F1 binding site on the *DHODH* promoter. **B.** The indicated TNBC cell lines were treated with either DMSO or BRL-50481 (50 µM) for 72 h. *E2F1* mRNA expression was measured by RT-qPCR. *E2F1* mRNA expression is plotted relative to DMSO-treated cells (n = 3 biological replicates/group). ACTINB was used as a normalization control. **C.** The indicated TNBC cell lines were treated with either DMSO or BRL-50481 (50 µM)

for 72 h. E2F1 protein expression was measured using immunoblot analysis under the indicated conditions. ACTINB was used as a loading control. **D.** The indicated TNBC cell lines were treated with either DMSO or BRL-50481 (50 μM) for 72 h, and were analyzed for assessing E2F1 binding on the *DHODH* promoter using CUT-&-RUN assay (n = 3 biological replicates/group). The ACTINB promoter was used as a control. IgG was used as a negative control for CUT-&-RUN assay, and fold-enrichment plotted relative to IgG under the indicated conditions is shown. **E.** The indicated TNBC cell lines expressing either non-specific (NS) shRNA or shRNAs targeting *E2F1* were analyzed for *E2F1* and *DHODH* mRNA expression using RT-qPCR. *E2F1* and *DHODH* mRNA expression is plotted relative to NS shRNA expressing cells (n = 3 biological replicates/group). ACTINB was used as a normalization control. **F.** The indicated TNBC cell lines expressing NS shRNA or *E2F1* shRNAs were analyzed for the expression of the indicated proteins by immunoblotting. ACTINB was used as a loading control. All quantitative data represent the mean ± SEM. *$p<0.05$, **$p<0.01$, ***$p<0.001$.

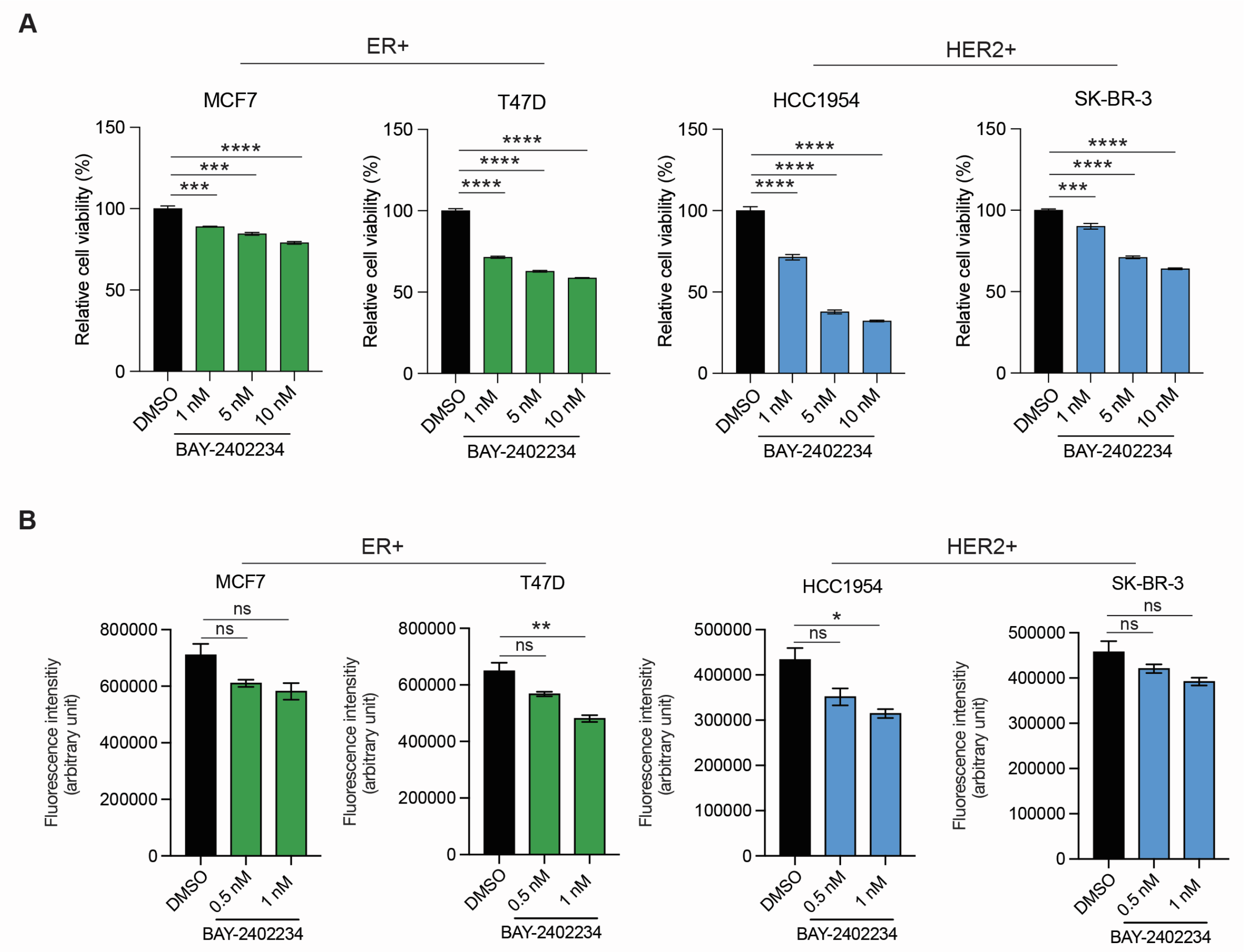

Figure S16. DHODH inhibition produces variable but largely marginal effect on the growth of non-TNBC cell lines. Related to Figure 5. A. The indicated breast cancer cell lines from the indicated subtypes were treated with DMSO or the DHODH inhibitor BAY-2402234 at the indicated concentrations for 72 h and analyzed for cell viability using the MTT assay. Relative cell viability is plotted compared to DMSO-treated cells (n = 5 biological replicates/group). **B.** The indicated breast cancer cell lines from the indicated breast cancer subtypes were treated with DMSO or BAY-2402234 at the indicated concentrations, and a quantitative soft-agar assay was performed using the CytoSelect 96-well quantitative soft agar assay. Fluorescence intensities (arbitrary unit) under the indicated conditions for the indicated breast cancer cell lines from the indicated breast cancer subtypes are shown (n = 3 biological replicates/group). All quantitative data represent the mean ± SEM. ns= not significant, *p<0.05, **p<0.01, ***p<0.001 and ****p<0.0001.

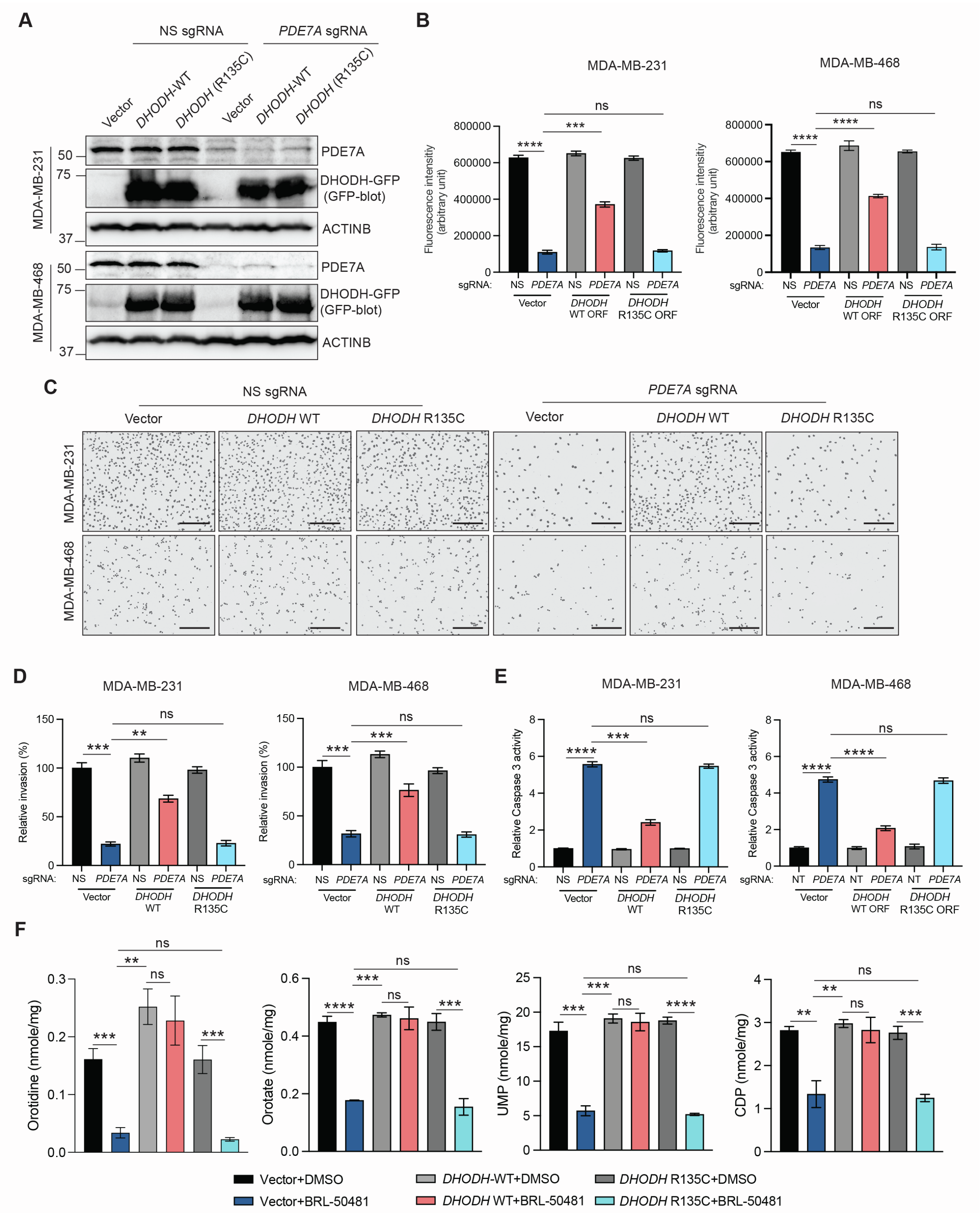

Figure S17. Enzymatic activity defective mutant DHODH (R135C), unlike enzymatic activity proficient wild-type DHODH fails to rescue PDE7A-loss driven phenotypes in TNBC cells. Related to Figure 6. A. The indicated TNBC cell lines expressing an empty vector, wild-type (WT) *DHODH* or catalytic mutant (R135C) *DHODH* ORF and simultaneously expressing non-

specific (NS) sgRNA or *PDE7A*-targeting sgRNA were analyzed for expression of the indicated proteins. ACTINB was used as the loading control. **B.** The indicated TNBC cell lines expressing an empty vector, wild-type (WT) *DHODH* or catalytic mutant (R135C) *DHODH* ORF and simultaneously expressing NS sgRNA or *PDE7A*-targeting sgRNA were analyzed using the CytoSelect 96-well quantitative soft agar assay kit. Fluorescence intensities (arbitrary unit) under the indicated conditions are shown (n = 3 biological replicates/group). **C.** The indicated TNBC cell lines expressing an empty vector, wild-type (WT) *DHODH* or catalytic mutant (R135C) *DHODH* ORF and simultaneously expressing NS sgRNA or *PDE7A*-targeting sgRNA were analyzed for invasive capacity using a Matrigel-based invasion assay. Representative images for the indicated cell lines under the indicated conditions are shown. Scale bar, 200 µm. **D.** Relative invasion (%) for the indicated cell lines under the indicated conditions is plotted for the experiment shown in panel C (n = 3 biological replicates/group). **E.** The indicated TNBC cell lines expressing an empty vector, wild-type (WT) *DHODH* or catalytic mutant (R135C) *DHODH* ORF and simultaneously expressing NS sgRNA or *PDE7A*-targeting sgRNA were analyzed for caspase-3 activity using a caspase 3-based colorimetric assay. Relative caspase-3 activity for the indicated cell lines under the indicated conditions is shown (n = 3 biological replicates/group). **F.** MDA-MB-231 cells expressing an empty vector, wild-type (WT) *DHODH* or catalytic mutant (R135C) *DHODH* ORF were treated with DMSO or BRL-50481 (50 µM) for 72 h, and were analyzed for the indicated metabolites. Indicated metabolites are presented in nmole/mg for the indicated conditions (n = 3 biological replicates/group). All quantitative data represent the mean ± SEM. ns= not significant, **p<0.01, ***p<0.001, ****p<0.0001.

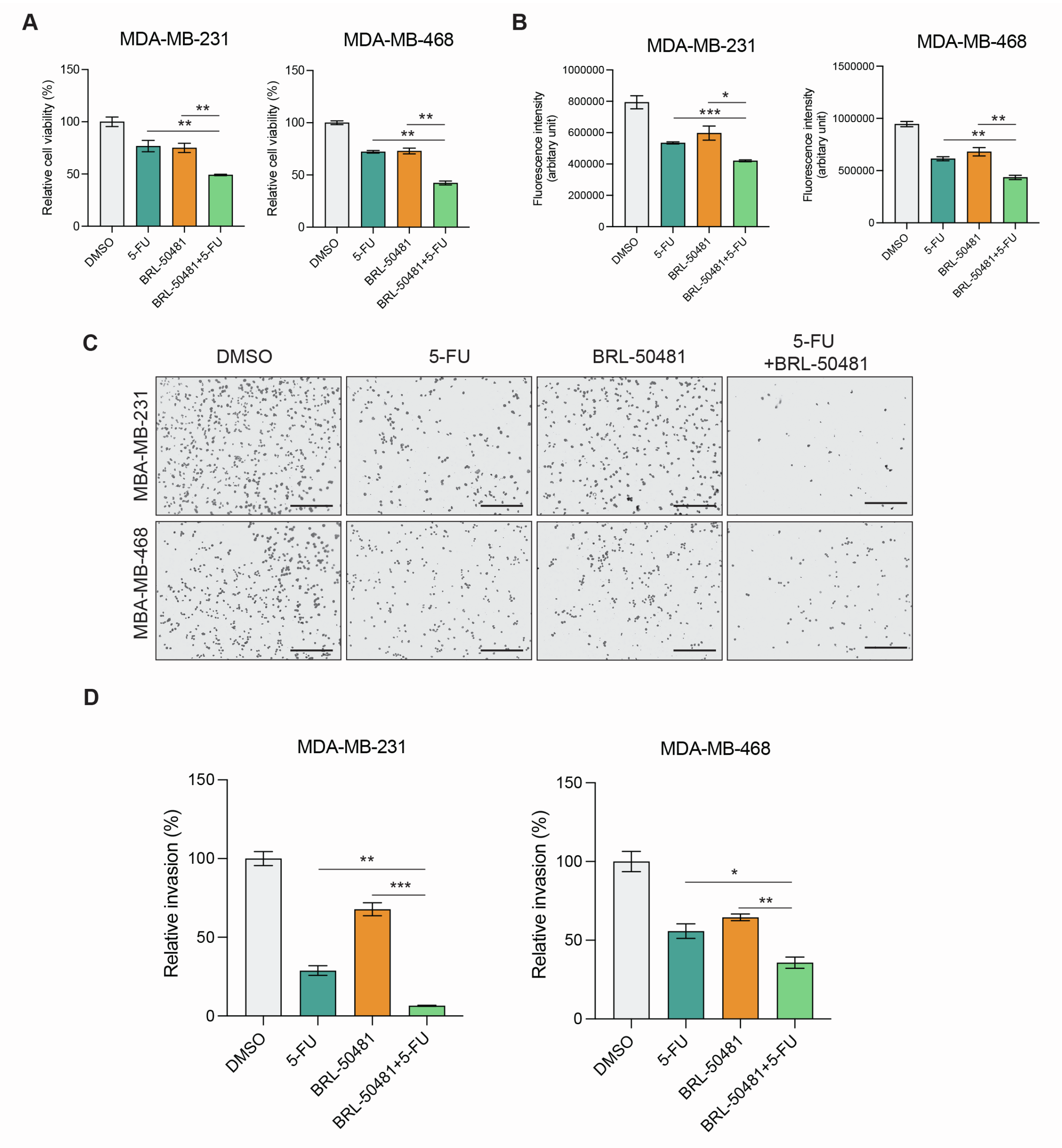

Figure S18. 5-FU enhances the growth inhibitory effect of BRL-50481 in TNBC cells. Related to Figure 6. A. The indicated TNBC cell lines were treated with DMSO, PDE7A inhibitor BRL-50481 (20 µM), 5-FU (10 µM) or with the combination of both BRL-50481 (20 µM) and 5-FU (10 µM) for 72 h, and were analyzed for cell viability using the MTT assay. Relative cell viability is plotted relative to DMSO-treated cells (n = 4 biological replicates/group). **B.** The indicated TNBC cell lines were treated with DMSO, PDE7A inhibitor BRL-50481 (20 µM), 5-FU (10 µM) or with the combination of both BRL-50481 (20 µM) and 5-FU (10 µM) and a quantitative soft-agar assay was performed using the CytoSelect 96-well quantitative soft agar assay. Fluorescence intensities (arbitrary unit) under the indicated conditions are shown (n = 3 biological

replicates/group). **C.** The indicated TNBC cell lines were treated with DMSO, PDE7A inhibitor BRL-50481 (20 μM), 5-FU (10 μM) or with the combination of both BRL-50481 (20 μM) and 5-FU (10 μM) for 20 h, and analyzed for invasive capacity using a Matrigel-based invasion assay. Representative images are shown. Scale bar, 200 μm. **D.** Relative invasion (%) relative to the DMSO-treated cells is plotted for the experiment shown in panel C (n = 3 biological replicates/group). All quantitative data represent the mean ± SEM. *p < 0.05, **p < 0.01, ***p < 0.001.
